# Toward Biomass-Based Organic Electronics: Continuous Flow Synthesis and Electropolymerization of *N*-Substituted Pyrroles

**DOI:** 10.1021/acsomega.3c08739

**Published:** 2024-03-12

**Authors:** Serena Frasca, Maxim Galkin, Maria Stro̷mme, Jonas Lindh, Johan Gising

**Affiliations:** Nanotechnology and Functional Materials, Department of Materials Science and Engineering, Ångström Laboratory, Uppsala University, 751 03Uppsala, Sweden

## Abstract

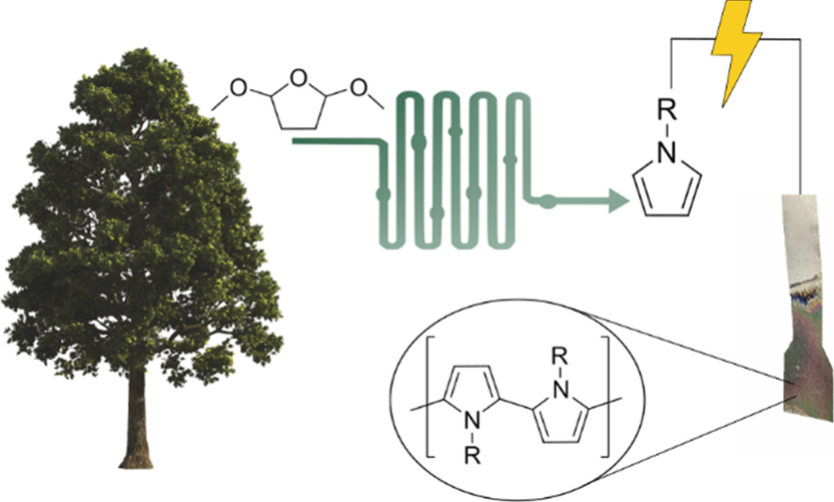

Pyrroles are foundational building blocks in a wide array of disciplines, including chemistry, pharmaceuticals, and materials science. Currently sourced from nonrenewable fossil sources, there is a strive to explore alternative and sustainable synthetic pathways to pyrroles utilizing renewable feedstocks. The utilization of biomass resources presents a compelling solution, particularly given that several key bulk and fine chemicals already originate from biomass. For instance, 2,5-dimethoxytetrahydrofuran and aniline are promising candidates for biomass-based chemical production. In this study, we present an innovative approach for synthesizing *N*-substituted pyrroles by modifying the Clauson-Kaas protocol, starting from 2,5-dimethoxytetrahydrofuran as the precursor. The developed methodology offers the advantage of producing pyrroles under mild reaction conditions with the potential for catalyst-free reactions depending upon the structural features of the substrate. We devised protocols suitable for both continuous flow and batch reactions, enabling the conversion of a wide range of anilines and sulfonamides into their respective *N*-substituted pyrroles with good to excellent yields. Moreover, we demonstrate the feasibility of depositing thin films of the corresponding polymers onto electrodes through in situ electropolymerization. This innovative application showcases the potential for sustainable, biomass-based organic electronics, thus, paving the way for environmentally friendly advancements in this field.

## Introduction

Pyrroles are widespread in the field of chemistry and continue to generate considerable interest due to their favorable properties. The motif is encountered in pharmaceutically active compounds and natural products, as well as in polymer chemistry, catalysis, and materials chemistry.^1−3^ Conductive materials based on polypyrrole (PPy) composites have received substantial attention for their applications in organic semiconductors (OSCs).^4−7^ OSCs offer a highly versatile alternative to their inorganic counterparts, primarily because of their potential for solution processing. They are increasingly recognized as indispensable components in the development of flexible, printable, and scalable electronics.^8^

There are several reported procedures^9^ to synthesize *N*-substituted pyrroles, including the Hantzsch,^10,11^ Paul–Knorr,^12^ and Clauson–Kaas methods.^13^ The Clauson-Kaas reaction serves as the primary synthetic pathway for 1,4-unsubstituted pyrroles. Different protocols for performing this reaction have been reported and recently summarized in a review by Kumar et al.^14^ The literature contains instances of the Clauson-Kaas protocol being executed without the need for a catalyst. Furthermore, reactions conducted under neat conditions or in water are documented. Additionally, modern energy transfer methods, such as microwave or acoustic radiation, have been reported as sustainable and environmentally friendly alternatives.^15−17^ Simultaneously, the advancement of flow chemistry plays a pivotal role in enhancing both small molecule synthesis^18^ and polymer production.^19^ Continuous flow synthesis maximizes process efficiency by minimizing both chemical and energy waste.^20^ The integration of monomer synthesis within a continuous flow reactor, followed by the polymerization step, presents an appealing strategy.^21^ Hence, a flow-chemistry-based approach for pyrrole synthesis using the Clauson-Kaas method becomes highly attractive.

Even though carbon-based conjugated polymers like PPy possess a lower environmental impact compared to their inorganic counterparts,^22^ their synthesis still heavily relies on petrochemicals.^23^ To enhance the sustainability of PPy, these polymers can be synthesized using renewable sources, such as biomass-derived materials.^24,25^ The common Clauson–Kaas reaction reagents 2,5-dimethoxytetrahydrofuran and anilines can be derived from lignocellulosic biomass. For instance, furan, obtained directly from biomass or with furfural as an intermediate,^26−28^ can be transformed into 2,5-dimethoxytetrahydrofuran.^29,30^ Additionally, a pathway from the lignin fraction of biomass to aniline has been outlined, yielding up to 13%, thereby enabling the execution of the Clauson-Kaas reaction using solely biomass-derived materials.^31,32^

In this study, we report a rapid and efficient method for synthesizing a set of *N*-substituted pyrroles through a Clauson–Kaas reaction procedure utilizing *para*-toluensulfonic acid (*p*TsOH) as a catalyst. Two distinct methods have been developed: a continuous flow process suitable for scaling out and a convenient, rapid batch method under milder conditions than the original procedure. These methods have demonstrated success in the synthesis of a wide range of anilines and sulphonamides. Furthermore, selected pyrrole monomer products were subjected to electropolymerization and were subsequently assessed as electrode coatings.

## Results and Discussion

The study was initiated in a flow reactor, operating on the principles of controlled resistive heating of a stainless-steel capillary with an inner diameter of 1 mm and a length of 1 m. Temperature control was achieved using a thermocouple located near the outlet of the capillary, which regulated the heating power (see SI Figure S1, for a detailed description). The screening commenced with a model reaction using aniline (**1,** 1 equiv, 0.08 M) as the substrate, reacting with 2,5-dimethoxytetrahydrofuran (**2**, 1.25 equiv) in the presence of acetic acid (pH 4.5–5.5) as the catalyst. To identify a suitable solvent, the study considered water, ethanol, and 1,4-dioxane as reaction media at various temperatures. The quantity of catalyst (30 mol % acetic acid) and the flow rate were held constant at 1 mL min^–1^, which equates to a 47 s residual time. The use of water as a solvent posed challenges due to the limited water solubility of the product and the formation of byproducts through in situ polymerization, consequently leading to the clogging of the flow reactor (Table 1, entry 1). While side reactions were less pronounced in ethanol, no product formation was observed even at higher temperatures (up to 180 °C, entry 2). However, when 1,4-dioxane was employed as a solvent at 120 °C, trace amounts of product formation were observed (entry 3). These initial findings prompted further exploration of 1,4-dioxane, accompanied by an extension of the reaction residual time (*t*_r_) and various temperatures. The flow was subsequently reduced to 0.25 mL min^–1^ (*t*_r_ = 3.14 min), and the temperature was incrementally raised to 220 °C (entries 4–8). The adjustments generated increasingly higher yields at each temperature step, resulting in a 43% crude ^1^H NMR yield at 220 °C (see SI for the ^1^H NMR yield protocol).

**Table 1:**
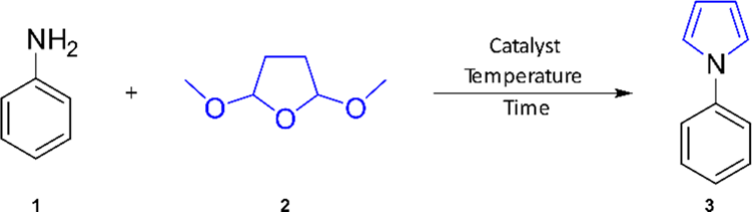
Optimization of the Reaction Conditions

entry	solvent	*T* [°C]	acid [mol %]	yield^a^ [%]	flow [mL min^–1^]
1	water	100	30 (AcOH)		1.00
2	ethanol	100 to 180	30 (AcOH)		1.00
3	1,4-dioxane	120	30 (AcOH)		1.00
4	1,4-dioxane	140	30 (AcOH)		0.25
5	1,4-dioxane	160	30 (AcOH)	5	0.25
6	1,4-dioxane	180	30 (AcOH)	9	0.25
7	1,4-dioxane	200	30 (AcOH)	22	0.25
8	1,4-dioxane	220	30 (AcOH)	43	0.25
9	1,4-dioxane	100	10 (H_2_SO_4_)	60	0.25
10	water/ethanol 1:1	100	10 (H_2_SO_4_)		0.25
11	1,4-dioxane	180	10 (H_2_SO_4_)	95	0.25
12	1,4-dioxane	180	10 (H_2_SO_4_)		0.10
13	1,4-dioxane	180	10 (H_2_SO_4_)	40	0.50
14	1,4-dioxane	180	25 (H_2_SO_4_)		0.25
15	1,4-dioxane	160	10 *p*TsOH	87	0.25
16	1,4-dioxane	180		16	0.25

a Yields were determined using internal standard and ^1^H NMR.

Next, we explored the impact of the catalysts. Given sulfuric acid’s stronger acidity in comparison to acetic acid, the catalyst loading was reduced. The use of 10 mol % sulfuric acid already increased the yield to 60% (entry 9) at a modest 100 °C temperature. Encouragingly, when the reaction temperature was raised to 180 °C (entry 11) in 1,4-dioxane with sulfuric acid, we achieved an excellent 95% yield. Subsequent investigations examined suitable flow rates in entries 11–13. Reducing the flow from 0.25 to 0.10 mL min^–1^ (*t*_r_ = 7.85 min) led to the formation of byproducts, likely related to polymerization, as evidenced by black residues clogging the instrument tubing. On the other hand, doubling the flow rate to 0.50 mL min^–1^ (*t*_r_ = 1.57 min) resulted in unstable pressure and a more than halved product yield (40%, entry 13). The earlier polymerization issue resurfaced when the sulfuric acid loading was increased to 25 mol % (entry 14). Maintaining a flow rate of 0.25 mL min^–1^ with 10 mol % sulfuric acid at 180 °C provided an excellent yield of 95% (entry 11). Consequently, we continued our investigation using the reaction conditions from entry 11 to assess the performance of another acid catalyst, *p*TsOH at 10 mol %. A very good yield of 87% was achieved using organic acid, even at a lower temperature of 160 °C (entry 15). An acidic environment is essential for pyrrole formation in the Clauson-Kaas protocol,^33^ as confirmed by the trace amount of product **3** obtained in the reaction without an acidic catalyst (entry 16). Both sulfuric acid and *p*TsOH proved to be superior catalysts to acetic acid in 1,4-dioxane, affording higher product yields at lower temperatures. Hence, we chose to continue exploring the reaction’s scope using *p*TsOH due to its superior performance at lower temperatures and reduced tendency for clogging the flow reactor compared to when sulfuric acid was employed.

With the optimal conditions in hand (Table 1, entry 15), we proceeded to explore the scope and limitations of the reaction with the results presented in Table 2. Anilines with substituents in the *para*-position provided the corresponding *N-*substituted pyrroles in varying yields, indicating that the reaction is affected by electronic effects (Table 2, compounds **4**–**10**). The less nucleophilic *p-*fluoroaniline (**4**) and *p-*nitroaniline (**7**) exhibited lower conversion to their corresponding substituted pyrrole products (27% and 19% isolated yield, respectively), whereas the electron-withdrawing effect of the *p*-trifluoro group had a less pronounced impact on the reaction outcome (51% yield, compound **10**). Likewise, *p-*chloro- and *p*-bromoanilines (**5** and **6**) resulted in moderate product formation (66% and 59% NMR yield), with lower isolated yields (35% and 46%, respectively). The more nucleophilic *para* methoxyaniline allowed for the isolation of **8** in 67% yield. When 4-aminobenzoic acid was used as a substrate, it was possible to synthesize the corresponding *N*-substituted pyrrole without the addition of an acid catalyst, leading to the synthesis of 4-pyrrole-1-ylbenzoic acid (**9**) with excellent product formation (91% NMR yield) and decent isolated yield (67%). Turning to aliphatic substituted anilines, it was generally observed that electron-donating groups provided higher product yields than anilines bearing electron-withdrawing groups. An *o*-methyl substituent was well accepted, affording the corresponding product **11** in 81% yield. However, with more steric bulk in the ortho position, the yield decreased for the corresponding isopropyl (**12**, 73% yield) and *t*-butyl (**13**, 65% NMR yield). The more sterically constrained compound **14** (1-(5,6,7,8-tetrahydronaphthalen-1-yl)pyrrole) was isolated in a similar yield (65%). Next, we explored the potential of nonaniline substrates under the same reaction conditions. The considerably stronger nucleophile benzylamine did not produce any product (results not shown), and the trial with benzamide (**15**) was not very successful, resulting in a 38% NMR yield. In contrast, benzenesulfonamide (**16**) was well accepted under the reaction conditions, and the product was isolated in 87% yield. These results imply that for the reaction to be successful under these conditions a sp^2^ hybridized atom in the α-position to the pyrrole nitrogen is preferable.

**Table 2:**
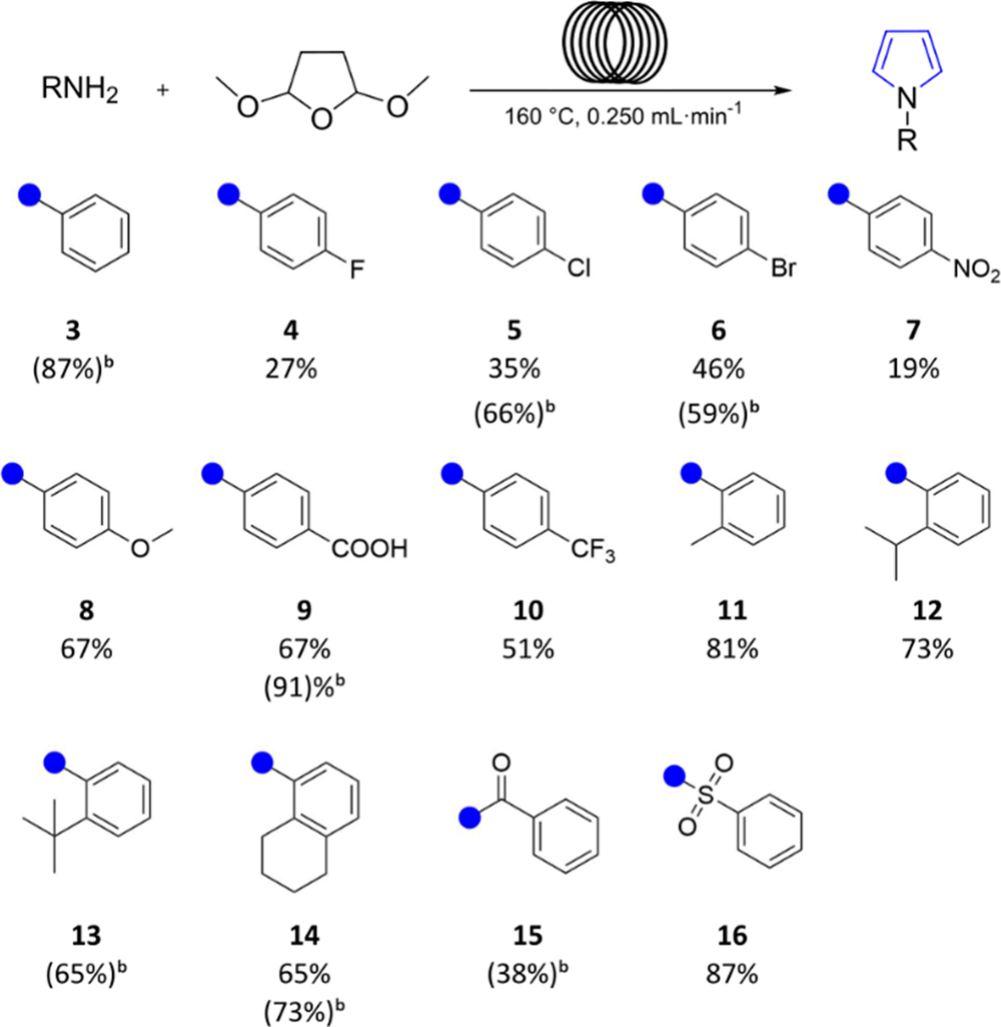
Scope and Limitation of the Approach in Continuous Flow^a^

a Isolated yields.

b NMR yields. Reaction conditions: amine (0.08 M, 1 equiv), 2,5-dimethoxytetrahydrofuran (0.10 M, 1.25 equiv), *p*-toluenesulfonic acid (10 mol %), 1,4-dioxane, 160 °C, 0.250 mL min^–1^.

Initial attempts to vary anilines under continuous flow conditions proved to be challenging for some compounds, resulting in poor pressure control and long downtimes for cleaning. Instead of forgoing interesting products, we opted to optimize a batch protocol by using a newly developed batch inductive heater. The heater is built around a resistive heating coil that is in close contact with an aluminum tube to enable rapid heat transfer to the reaction vial (see Figure S2, for a detailed description). Trial reactions were conducted under reaction conditions similar to those used in the flow reaction protocol. In a dedicated glass vial, aniline (**1**, 1.00 mmol, 1 equiv), 2,5-dimethoxytetrahydrofuran (**2**, 1.25 equiv), and *p*TsOH (10 mol %) in 1,4-dioxane (6 mL) were subjected to controlled inductive heating (0.17 M of aniline). The temperature screening commenced at 120 °C for 10 min, resulting in a crude ^1^H NMR yield of 52% (Table 3, entry 1). As the temperature was raised, the yield increased, with full consumption of the starting material at 160 °C, yielding 97% of *N-*phenylpyrrole **3** (entry 3).

**Table 3:**
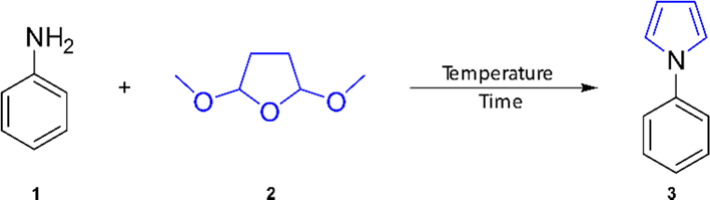
Optimization of Batch Reaction Conditions

entry	temperature [°C]	time [min]	yield^a^ [%]
1	120	10	52
2	140	10	91
3	160	10	97

a Yields are determined using internal standard and ^1^H NMR.

With appropriate reaction conditions established for the batch protocol, we investigated the scope and limitations of the approach (Table 4). Notably, it was found that a liquid–liquid extraction process was often sufficient for product isolation. In general, the batch protocol yielded the desired products in good to excellent yields, displaying less selectivity among the various substituents tested compared to the flow protocol. *Para-*substituted anilines, bearing electron-withdrawing or electron-donating groups, produced the desired isolated products at approximately 70% yield, demonstrating good acceptance for hydroxyl, ester, and sulfonamide (Table 4, compounds **17**–**19**). The reaction exhibited chemoselectivity in the formation of product **19,** proceeding exclusively at the aniline group over the benzosulfonamide.

**Table 4:**
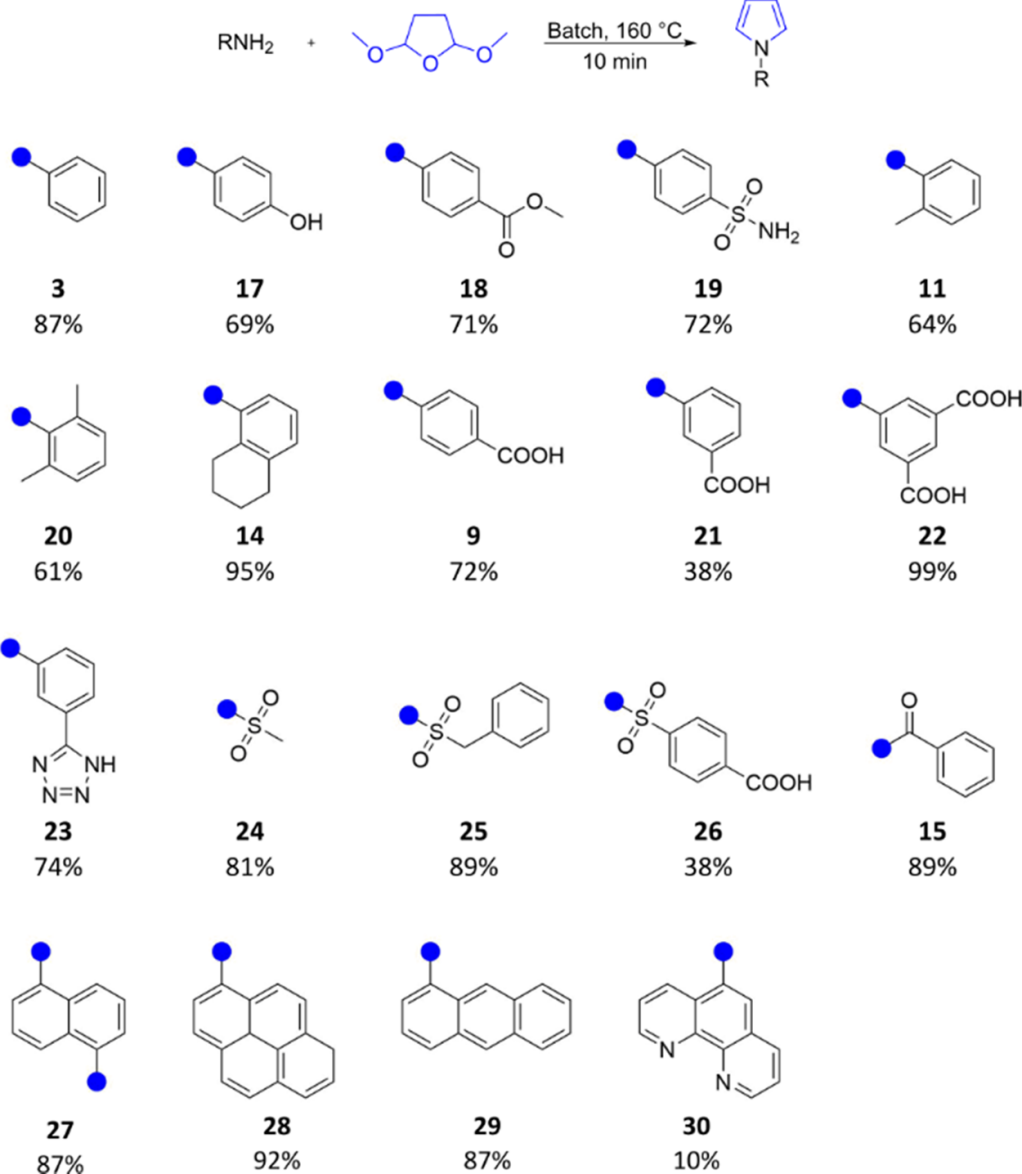
Scope and Limitation of the Approach in Batch^a^

a Isolated yields reaction conditions: amine (1.0 mmol, 1 equiv), 2,5-dimethoxytetrahydrofuran (1.25 equiv), 1,4-dioxane (6 mL), *p*-toluenesulfonic acid (10 mol %), 160 °C, 10 min.

*Ortho*-substituted anilines were obtained in moderate to excellent yields (Table 4, compounds **11**, **20,** and **14**), with the best outcome observed for bicycle **14** (95% yield). Encouraged by the results obtained in flow with 4-aminobenzoic acid, we performed the synthesis with anilines bearing one or two acidic moieties without a catalyst (compounds **9**, **21**, **22**, and **26**). While the reaction outcome was great for compound **22** (99% yield) and acceptable for *para* carboxylic acid **9** (72% yield), only moderate conversion was achieved for compound **21** (38% yield). Methyl esters were observed in the reaction mixtures for compound **21**, decreasing the amount of available catalyst and requiring thorough purification. The isosteric replacement of the carboxyl group at the *m*-position of the benzene ring with a tetrazolyl resulted in a doubled yield (**21** vs **23**). Both aliphatic and benzylic sulfonamides were well accepted and converted to the desired products in very good yields (**24** and **25**, > 80% yield). The acidic substrate 4-sulfamoylbenzoic acid was used under catalyst-free conditions, but the reaction outcome was not as good as expected, yielding only 38% of compound **26**. Benzamide (**15**) was previously troublesome and had a low yield under flow conditions (38% yield), but it was successfully isolated here in a high yield (89%). Polyaromatic substrates were also explored, and even sterically hindered amines yielded products in very good or excellent yields (**27**–**29**, 87–92% yield). However, structurally similar 5-amino-1,10-phenanthroline provided a yield of only 10%, likely due to the deactivation of the catalyst through pyridinium salt formation (**30**).^34^

Next, the newly synthesized monomers were utilized as substrates for polymerization. Electropolymerization was favored over chemical polymerization due to its superior ability to control film thickness and morphology.^35^ Previously reported conditions for electropolymerization were employed.^36^ Several *N*-substituted pyrroles with various properties, *N*-phenylpyrrole **3**, 1-phenylsulfonylpyrrole **16**, 1-(benzylsulfonyl)-1*H*-pyrrole **25**, and 1-anthracen-2-ylpyrrole **29** were selected for the polymerization study. Electropolymerization was performed in a divided cell, using flexible graphite paper as the working electrode in acetonitrile at a substrate concentration of 5 mM. Potentials were reported versus an Ag/AgCl reference electrode, with a Pt wire as the counter electrode, and 0.1 M tetrabutylammonium hexafluorophosphate (TBAPF_6_) served as the supporting electrolyte. The electropolymerization involved repetitive potential scanning (x20) between −1.8 and +1.8 V at a scanning rate of 50 mV s^–1^. As the scanning progressed, a noticeable color change occurred in the solution near the surface of the working electrode. This solution transformed into a deeper shade of brown due to the oxidation of some monomers into oligomers, which then dispersed in the solution. A prominent oxidation current wall was observed at potentials exceeding 1.0 V, leading to the formation of a brownish film on the working electrode’s surface while in solution. This aligns with previously reported values for the electropolymerization of *N*-substituted pyrroles.^37−40^

**Figure 1:**
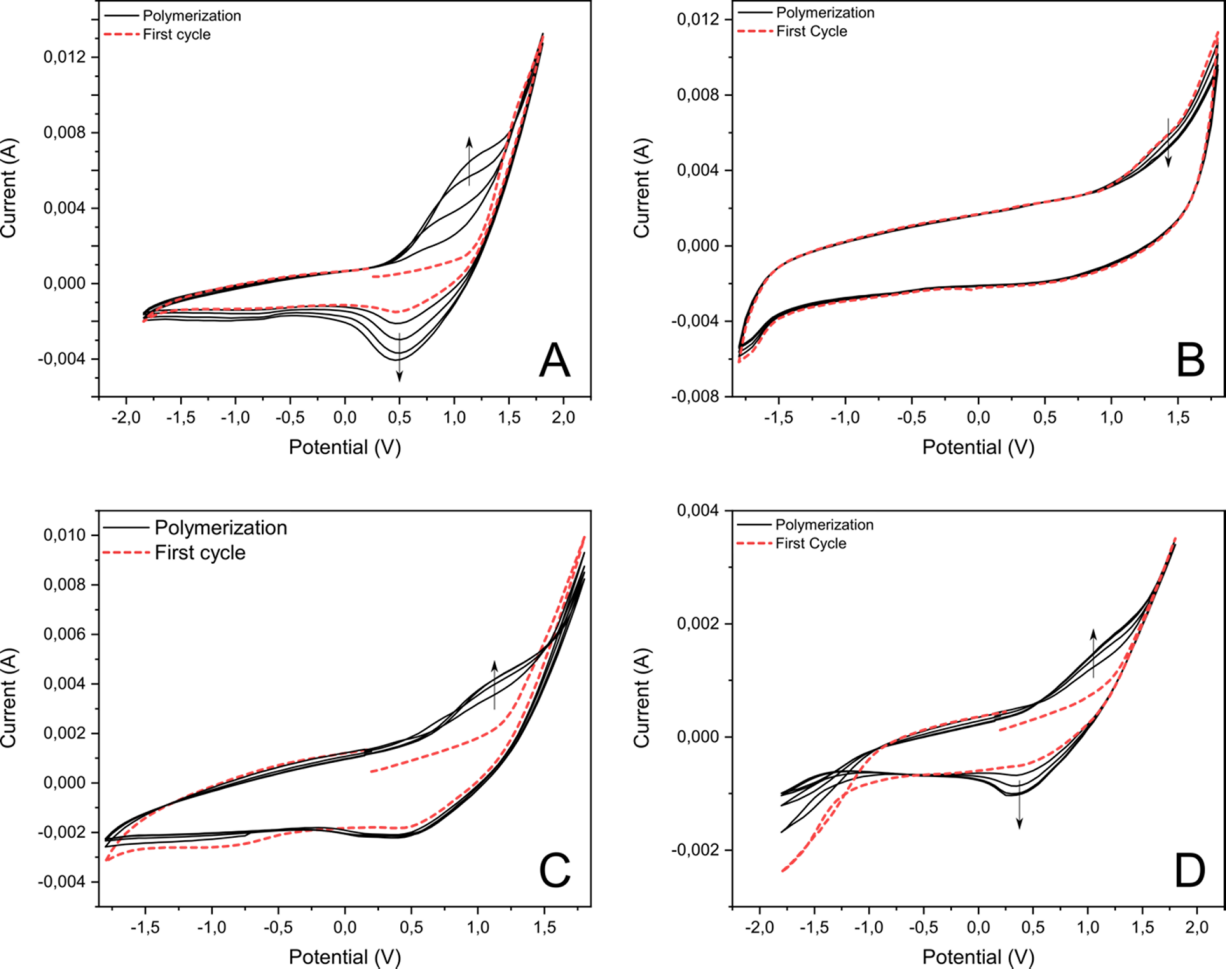
Cyclic voltammogram recorded in a potential window of −1.8 to 1.8 V vs Ag/AgCl with a scan rate of 50 mV s^−1^ in 0.1 M TBAPF_6_ during electropolymerization of compounds: *N*-phenylpyrrole **3** (A), 1-phenylsulfonylpyrrole **16** (B), 1-(benzylsulfonyl)-1*H*-pyrrole **25** (C), and 1-anthracen-2-ylpyrrole **29** (D).

The entire CV peak current increases gradually with the number of CV cycles performed, indicating the growth of a conducting film. Reversible oxidation peaks were observed after the first cycle in the +0.2 to +0.75 V potential range for film forming with monomers **3**, **25**, and **29** (Figure 1). Furthermore, these peaks increased with the number of potential cycles, suggesting that the observed oxidation and reduction processes are associated with the formed polypyrrole. On the other hand, compound **16** did not exhibit any oxidation or reduction peaks. This suggests that the partially formed oligomers may have been soluble in the reaction medium, leading to their continuous removal from the electrode surface during the process. Subsequently, each electrode with deposited films of compounds **3**, **25**, and **29** was rinsed with acetonitrile to eliminate any unreacted starting material. During this process, a brownish coating was visibly detected on the electrode’s surface. The obtained electrodes were used as working electrodes for CV experiments (see SI Figure S3). The CV curves of the deposited polymers were recorded at a scanning rate of 20 mV s^–1^ (H_2_SO_4_ = 0.5 M), in a three-electrode electrochemical cell, using a Pt wire as the counter electrode and Ag/AgCl as the reference electrode in a monomer-free solution. The examination of CV curves revealed that the electrodeposited films for compounds **3**, **25**, and **29** remained stable under the scanning conditions and showed a reduced specific capacitance compared with the uncoated electrode. The insulating effect arising from the polymerized films can be attributed to the hydrophobic nature of the N-substituents.

**Figure 2:**
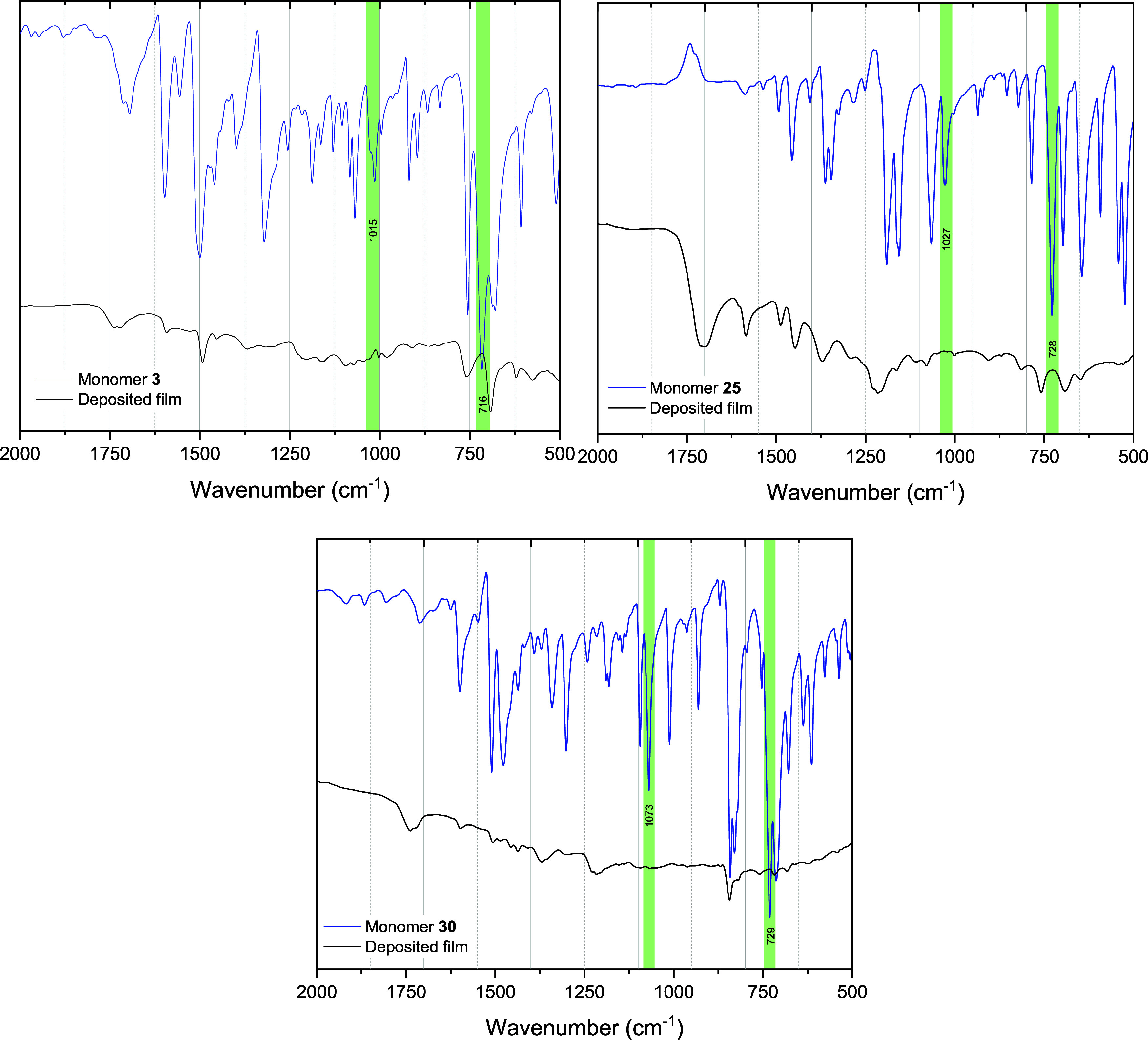
FTIR spectra of compounds **3**, **25**, and **29** and their corresponding deposited films.

To further characterize the obtained materials, the FTIR spectra of the monomers were compared with the spectra of their corresponding deposited films on graphite foil (Figure 2). The spectra of the monomers were in accordance with previously reported data.^41,42^ The absorption lines at 3144–3046 cm^–1^ (aromatic C–H stretching) and 1600–1320 cm^–1^ (C=C, C–N stretching of aromatic units) were observed. In the monomer spectra, the signals at 1015 and 715 cm^–1^ are characteristic of out-of-plane vibrations of the C–H bond in the unsubstituted pyrroles. The disappearance of these signals in the corresponding polymer spectrum confirmed that the deposited films resulted from polymerization.^41,43^

## Conclusions

In summary, a Clauson–Kaas reaction method was developed for synthesizing *N*-substituted pyrroles, under both continuous flow and batch conditions. This approach involved the reaction of a diverse range of aromatic amines and sulfonamides with 2,5-dimethoxytetrahydrofuran, resulting in the production of *N*-functionalized pyrroles in high yields. Importantly, the method simplifies product isolation by utilizing a liquid–liquid extraction process. Furthermore, electropolymerization was carried out on functionalized pyrroles, namely *N*-phenylpyrrole, 1-(benzylsulfonyl)-1*H*-pyrrole, and 1-anthracen-2-ylpyrrole, to coat graphite foil electrodes with polymeric films. These films were characterized using CV and FTIR. The synthetic protocols provide access to a wide array of *N*-substituted pyrroles using starting materials that can be derived from biobased sources. The initial findings from this work lay the foundation for developing strategies to tailor the physicochemical properties of polypyrrole beyond its intrinsic characteristics. This can be achieved through the introduction of functional groups, potentially enhancing its sensing capabilities for various analytes, modulating electrode kinetics, or incorporating photochromic groups to make the polymer photoresponsive.

## Materials and Methods

All chemicals and solvents are commercially available and were used as received without further purification. The chromatographic column separations were performed by flash chromatography (silica gel, fumed powder, (0.2–0.3 μm avg. part. size). Thin layer chromatography (TLC) was performed on TLC silica gel aluminum foils and visualized under UV light (wavelength 254 nm). ^1^H and ^13^C NMR spectra were recorded on a JEOL (400YH magnet) Resonance 400 MHz spectrometer. Chemical shifts δ are reported in parts per million relative to solvents CDCl_3_ (^1^H: δ = 7.27; ^13^C: δ = 77.16) or DMSO-*D*_*6*_ (^1^H: δ = 2.50; ^13^C: δ = 39.52). Coupling constants *J* are reported in Hz. High-resolution mass spectra (HRMS) were recorded on a mass spectrometer equipped with an ESI source and a 7-T hybrid linear ion trap. FT-IR measurements were recorded on a Tensor 27 spectrometer (Bruker, Billerica, MA, USA) by using a platinum-attenuated total reflectance (ATR) accessory.

### General Procedure 1. Continuous Flow Reactor Synthesis of *N*-Substituted Pyrroles

Aromatic amine (4 mmol) and 2,5-dimethoxytetrahydrofuran (5 mmol) were added to 50 mL of 1,4-dioxane to give a 0.08:0.10 M solution of aromatic amine:2,5-dimethoxytetrahydrofuran. The solution was sonicated for 10 min before the addition of *p*-TsOH (0.4 mmol, 10 mol %). The pump was then turned on and kept at a flow rate of 0.250 mL min^–1^ with a corresponding residence time of 3.14 min in the stainless-steel capillary (inner diameter of 1 mm and length of 1 m). The sample was collected for 1 h and concentrated under reduced pressure. The residue was diluted with water and extracted with diethyl ether (3 × 20 mL). The organic phase was dried with Na_2_SO_4_ and the solvent was removed under reduced pressure. Unless otherwise stated, the crude material was purified by flash column chromatography over silica gel.

### General Procedure 2. Batch Synthesis of *N*-Substituted Pyrroles

A vial was charged with aromatic amine (1 mmol, 1 equiv), 2,5-dimethoxytetrahydrofuran (1.25 equiv), *p*-TsOH (0.1 mmol), and 1,4-dioxane (6 mL). The reaction vessel was sealed and heated in an inductive heater at 160 °C for 10 min under stirring. After cooling, the reaction mixture was concentrated under reduced pressure. The residue was diluted with water and extracted with diethyl ether (3 × 20 mL). The organic phase was dried with Na_2_SO_4_ and the solvent was removed under reduced pressure.

### General Procedure for Electrochemical Measurements of *N*-Substituted Pyrroles

Electrochemical polymerization and conductance measurements were performed by using an Autolab PGSTAT032N potentiostat from Metrohm AG, equipped with a bipotentiostat module. Each measurement was performed at room temperature and under N_2_ atmosphere. Polymerization was performed in a divided cell utilizing graphite paper as a working electrode; a Pt wire was used as the counter electrode, and potentials were reported versus an Ag/AgCl reference electrode. Acetonitrile was used as a solvent for the polymerization and tetrabutylammonium hexafluorophosphate (0.1 M, TBAPF_6_) was used as the supporting electrolyte. Monomers **3**, **16**, **25**, and **29** were dissolved to 5 mM in 0.1 M TBAPF_6_/MeCN and polymerized for 20 cycles between −1.8 and 1.8 V vs Ag/AgCl at a scan rate of 0.05 V/s. The obtained films on graphite paper were then characterized in a three-electrode electrochemical cell utilizing a Pt wire as the counter electrode in 0.5 M H_2_SO_4_. CV curves were recorded between −0.5 and +0.9 V vs Ag/AgCl at a scan rate of 0.02 V/s.

## Supporting Information Available

Additional experimental details, analytical data, and ^1^H and ^13^C NMR spectra for all compounds (PDF)
