# Supplementary material for: Toward Biomass-Based Organic Electronics: Continuous Flow Synthesis and Electropolymerization of N-Substituted Pyrroles
Source: ACS Omega. 2024 Mar 12;9(12):13852–9. doi: 10.1021/acsomega.3c08739 (PMC10975589; doi:10.1021/acsomega.3c08739)
Supplement: Supplementary file 1 — ao3c08739_si_001.pdf [file ao3c08739_si_001.pdf]

Supporting information

Toward biomass-based organic electronics: continuous flow synthesis and electropolymerization of *N*-substituted pyrroles

Serena Frasca, Maxim Galkin, Maria Strømme, Jonas Lindh, Johan Gising*

Nanotechnology and Functional Materials, Department of Materials Science and Engineering, Ångström Laboratory, Uppsala University, 751 03, Uppsala, Sweden

*johan.gising@angstrom.uu.se

Table of Contents

GENERAL PROCEDURE 1. CONTINUOUS FLOW REACTOR SYNTHESIS OF *N*-SUBSTITUTED PYRROLES S2

GENERAL PROCEDURE 2. BATCH SYNTHESIS OF *N*-SUBSTITUTED PYRROLES. S3

SCHEME OF THE BATCH HEATER S4

N-PHENYLPYRROLE (3) S5

1-(4-FLUOROPHENYL)-1*H*-PYRROLE (4) S6

1-(4-CHLOROPHENYL)-1*H*-PYRROLE (5) S6

1-(4-BROMOPHENYL)-1*H*-PYRROLE (6) S6

N-(4-NITROPHENYL)PYRROLE (7) S6

1-(*P*-METHOXYPHENYL)PYRROLE (8) S7

4-(1*H*-PYRROLE-1-YL)BENZOIC ACID (9) S7

1-(4-(TRIFLUOROMETHYL)PHENYL)-1*H*-PYRROLE (10) S7

1-*O*-TOLYL-1*H*-PYRROLE (11) S7

1-(2-ISOPROPYLPHENYL)PYRROLE (12) S8

1-(2-*TERT*-BUTYLPHENYL)PYRROLE (13) S8

1-(5,6,7,8-TETRAHYDRONAPHTHALEN-1-YL)PYRROLE (14) S8

N-BENZOYLPYRROLE (15) S9

N-PHENYLSULFONYLPYRROLE (16) S9

4-(1*H*-PYRROL-1-YL)PHENOL (17) S9

METHYL 4-(1*H*-PYRROL-1-YL)BENZOATE (18) S9

(4-PYRROL-1-YLPHENYL)SULFONAMIDE (19) S10

1-(2,6-DIMETHYLPHENYL)-1*H*-PYRROLE (20) S10

3-(1-PYRROLYL)BENZOIC ACID (21) S10

5-(1*H*-PYRROL-1-YL)BENZENE-1,3-DICARBOXYLIC ACID (22) S10
N-(3-(1H-TETRAZOL-5-YL)PHENYL)PYRROLE (23) S11
1-METHYLSULFONYLPYRROLE (24) S11
1-(BENZYLSULFONYL)-1*H*-PYRROLE (25) S11
1-(4-CARBOXYPHENYLSULFONYL)-1*H*-PYRROLE (26) S12
1,5-BIS-(PYRROL-1'-YL)-NAPHTHALIN (27) S12
1-(PYREN-1-YL)-1*H*-PYRROLE (28) S12
1-ANTHRACEN-2-YLPYRROLE (29) S12
5-(1*H*-PYRROL-1-YL)-1,10-PHENANTHROLINE (30) S13
CYCLIC VOLTAMMOGRAMS CURVES OF THE DEPOSITED FILMS S14
FTIR SPECTRA S15
1H NMR AND 13C NMR SPECTRA OF PRODUCTS S17
REFERENCES S49

General procedure 1. Continuous flow reactor synthesis of N-substituted pyrroles

Aromatic amine (4 mmol) and 2,5-dimethoxytetrahydrofuran (5 mmol) were added to 50 mL of 1,4-dioxane to give a 0.08:0.10 M solution of aromatic amine: 2,5-dimethoxytetrahydrofuran. The solution was sonicated for 10 minutes before the addition of *p*-TsOH (0.4 mmol). The pump was then turned on and kept at a flow rate of 0.250 mL min-1 with a corresponding residence time of 3.14 min in the pre-heater unit (1) (figure S1, stainless steel capillary, inner diameter 1 mm, length 1 m, cartridge (8) was not used). Sample was collected for one hour and concentrated under reduced pressure. The residue was diluted with water and extracted with diethyl ether (3 x 20 mL). The organic phase was dried with Na_2SO_4 and the solvent removed under reduced pressure. Unless otherwise stated, the crude material was purified by flash column chromatography over silica gel.

Scheme of the flow reactor

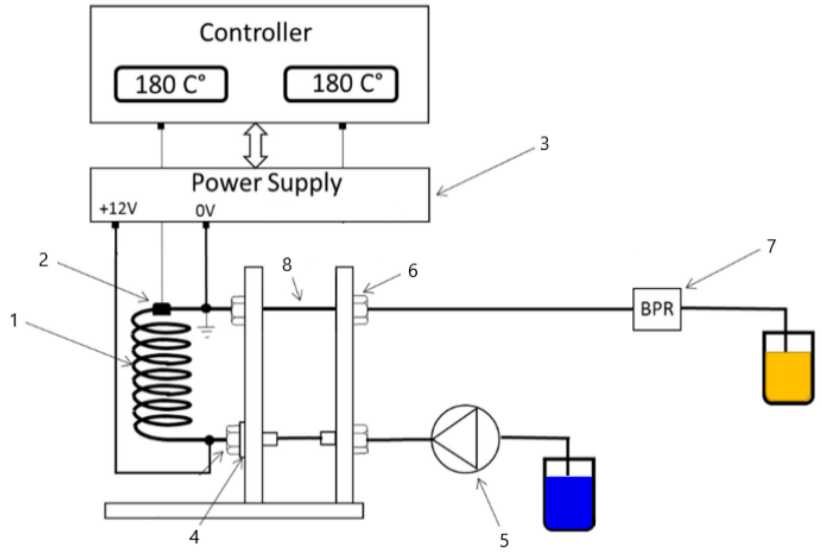

Figure S1. Schematic reactor representation. BPR, back-pressure regulator.

The pre-heater (1) consists of a coil reactor made of a stainless-steel capillary. The pre-heater is heated by resistive heating by passing a current through the capillary. The volume of the pre-heater can be easily changed by varying the inner diameter and the length of the capillary. The capillary will be the heating element and the heat transfer to the liquid will be through convection. The temperature is controlled by having a thermocouple (2) close to the outlet and connected to the controller and thereby regulating the temperature at the outlet to the desired value. The regulator is a PID type regulator with a switched solid-state relay. The power supply (3) providing the power has its 12V connected to the inlet side of the coil and it is galvanically isolated from the rest of the instrument by an isolator (4). The inlet of the system is connected to the pump (5) by a piece of PEEK tubing to further galvanically isolate the inlet from the +12 V. The outlet (6) end piece has a spring-loaded sealing mechanism to compensate for a thermal movement of the components and secure a leak tight connection over the whole temperature and pressure range. To maintain the necessary backpressure in the system a back-pressure regulator (7) is mounted on the outlet (6) of the system. The back-pressure regulator could generate a variable back pressure of 1-150 bar. The system can be run with a cartridge, in our case the cartridge was replaced with a bypass capillary (8). Retention times (t_r) were calculated accounting only for the volume of the pre-heater (1) coil reactor (0.785 mL).

General procedure 2. Batch synthesis of *N*-substituted pyrroles

A dedicated vial (Biotage, reaction vial, 351521) was charged with aromatic amine (1 mmol), 2,5-dimethoxytetrahydrofuran (1.25 mmol), p-TsOH (0.1 mmol) and 1,4-dioxane (6 mL). The reaction

vessel was sealed and heated in the batch inductive heater at 160 °C for 10 minutes with stirring (fig S2). After cooling, the reaction mixture was concentrated under reduced pressure. The residue was diluted with water and extracted with diethyl ether (3 x 20 mL). The organic phase was dried with Na_2SO_4 and the solvent removed under reduced pressure. Unless otherwise stated, the crude material was purified only by liquid-liquid extraction.

Scheme of the Batch heater

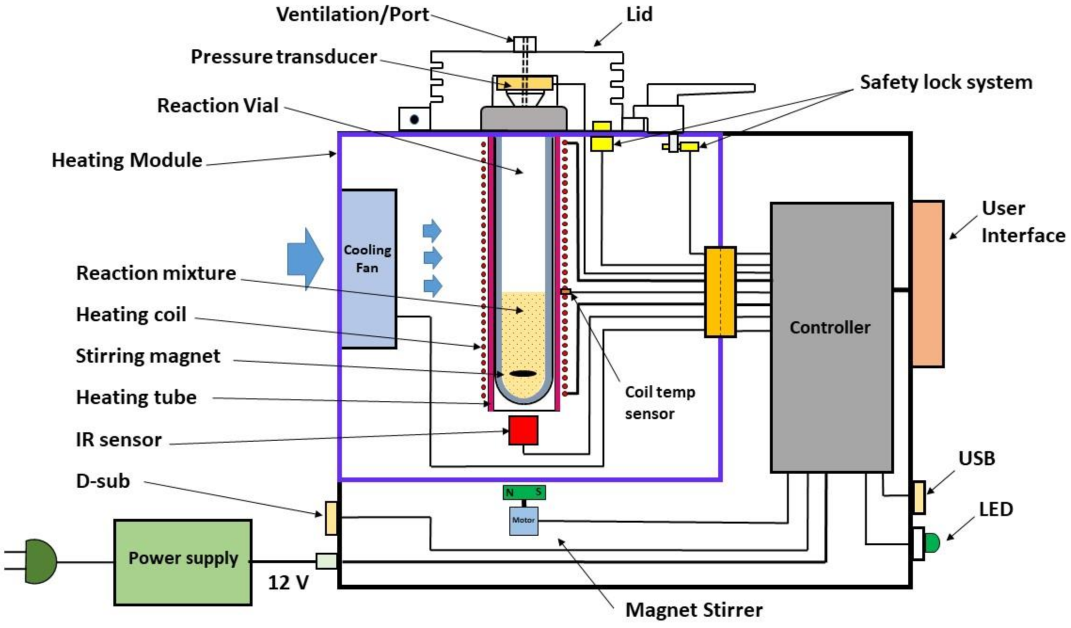

Figure S2. Schematic reactor representation.

The batch heater is a newly developed product (from Radeq Nordic AB, Uppsala) in late prototype stage aiming at a product for chemistry using high temperature and pressure that is safe and easy to use. The heating device is based on a heating coil made of a resistive material, in this case a Kanthal material. The heat is generated by driving a current through the coil. The coil is tightly wounded around a thin aluminum tube, enabling fast heat transfer to the reaction mixture. The instrument has a safety lock system ensuring that the instrument cannot be started or opened if the vial is pressurized or has a temperature over 60 °C. The heating module is a shielded compartment preventing spillage or glass debris from spreading if an overpressure situation should occur. The compartment and the heating module are easy to clean and/or service. Temperature is measured by using an IR-sensor monitoring the bottom of the reaction vial. A pressure transducer, placed in the lid, measures the internal pressure in the vial through a flexible septum at the top of the reaction vial. The lid has a ventilation port which can be used for releasing any remaining overpressure in the vial before opening

the lid. The port can also be used as an inlet port for adding gas or other reagents. Another use of the port is to insert a thermo-element for exact temperature measurements or calibration purposes. A speed adjustable steering magnet device is placed at the bottom of the heating module. The reaction mixture will automatically be cooled down to a pre-set level (less than 60 °C) at the end of the heating cycle. The run parameters can be set in the user interface (time, temperature and pressure) and the software has an integrated algorithm for detecting the heating characteristics of the reaction mixture and thereby avoiding thermal overshot and/or unnecessary long heating time. System status will be indicated on the three LEDs at the front of the instrument. The instrument is powered by an external power supply delivering 12 V DC. The low voltage ensures high electric safety. The instruments footprint is 130x180x260 mm.

Determination of ^{1}H NMR yields

NMR yields were determined using 1,3,5-trimethylbenzene as internal standard (^{1}H, 6.83 ppm). After collection of the reaction crude from flow and solvent evaporation, weighted amounts of the internal standard were added to weighted amounts of the resulting crude in a NMR tube. The ^{1}H NMR yields were determined by following equation (2).

$$N_2 = N_1 \cdot \frac{I_2/n_2}{I_1/n_1} \qquad (1)$$

$$Yield\ (\%) = \frac{N_2}{N_{pyrrole}} \cdot 100\% \qquad (2)$$

Where N_1 and N_2 are mole quantities of 1,3,5-trimethylbenzene and product, respectively; I_1 and I_2 are NMR integral values of 1,3,5-trimethylbenzene and product, respectively: n_1 and n_2 are number of nuclei responsible for that particular resonance of 1,3,5-trimethylbenzene and product, respectively.

N-Phenylpyrrole (**3**)

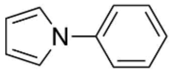

Prepared according to general procedure 1 (not isolated, ^{1}H NMR yield 81%). Prepared according to general procedure 2 and purified by liquid-liquid extraction. The product was obtained in isolated yield as yellow solid (0.87 mmol, 0.1244 g, 87%). Spectral data are consistent with previously reported data in literature[1]: ^{1}H NMR (400 MHz, $CDCl_3$) δ 7.47 – 7.35 (m, 4H), 7.31 – 7.19 (m, 1H), 7.13 – 7.07 (m, 2H), 6.40 – 6.33 (m, 2H). ^{13}C NMR (101 MHz, $CDCl_3$) δ 140.9, 129.7, 125.8, 120.7, 119.5, 110.5.

1-(4-fluorophenyl)-1*H*-pyrrole (**4**)

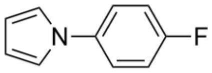

Prepared according to general procedure 1 and purified by flash chromatography (10-50% ethyl acetate in petroleum ether). The product was obtained in isolated yield as yellow solid (0.55 mmol, 0.089 g, 27% from 25 mL of 81 mM solution). Spectral data are consistent with previously reported data in literature[2]: ^{1}H NMR (400 MHz, $CDCl_3$) δ 7.40 – 7.30 (m, 2H), 7.17 – 7.07 (m, 2H), 7.04 – 6.99 (m, 2H), 6.37 – 6.32 (m, 2H). ^{13}C NMR (101 MHz, $CDCl_3$) δ 160.8 (d, J = 244.9 Hz), 137.3, 122.4 (d, J = 8.2 Hz), 119.8, 116.4 (d, J = 22.8 Hz), 110.6. ^{19}F NMR (376 MHz, $CDCl_3$) δ -117.01.

1-(4-chlorophenyl)-1*H*-pyrrole (**5**)

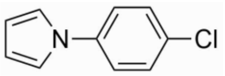

Prepared according to general procedure 1 and purified by flash chromatography (10-50% ethyl acetate in petroleum ether). The product was obtained in isolated yield as brown solid (1.38 mmol, 0.244 g, 35% from 25 mL of 80mM solution). Spectral data are consistent with previously reported data in literature[2]: ^{1}H NMR (400 MHz, $CDCl_3$) δ 7.39 (ddd, J = 8.9, 2.8, 2.8 Hz, 2H), 7.33 (ddd, J = 8.9, 2.8, 2.3 Hz, 2H), 7.08 – 7.02 (m, 2H), 6.38 – 6.33 (m, 2H).^{13}C NMR (101 MHz, $CDCl_3$) δ 139.5, 131.2, 129.8, 121.8, 119.4, 111.0.

1-(4-bromophenyl)-1*H*-pyrrole (**6**)

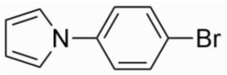

Prepared according to general procedure 1 and purified by liquid-liquid extraction (diethyl ether – water). The product was obtained in isolated yield as yellow solid (1.38 mmol, 0.244 g, 46% from 25 mL of 80mM solution). Spectral data are consistent with previously reported data in literature[2]: ^{1}H NMR (400 MHz, $CDCl_3$) δ 7.54 (ddd, J = 8.9, 3.0, 2.2 Hz, 2H), 7.27 (ddd, J = 9.0, 3.0, 2.2 Hz, 2H), 7.07 – 7.04 (m, 2H), 6.37 – 6.34 (m, 2H). ^{13}C NMR (101 MHz, $CDCl_3$) δ 139.9, 132.7, 122.1, 119.3, 118.8, 111.0.

N-(4-nitrophenyl)pyrrole (**7**)

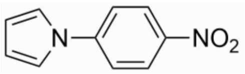

Prepared according to general procedure 1 and purified by flash chromatography (10-50% ethyl acetate in petroleum ether). The product was obtained in isolated yield as yellow solid (0.821 mmol, 0.155 g, 19% from 25 mL of 80mM solution). Spectral data are consistent with previously reported data in literature[3]: ^{1}H NMR (400 MHz, $CDCl_3$) δ 8.31 (ddd, *J* = 9.1, 3.0, 2.1 Hz, 2H), 7.52 (ddd, *J* = 9.1, 3.0, 2.1 Hz, 2H), 7.21 – 7.15 (m, 2H), 6.46 – 6.40 (m, 2H). ^{13}C NMR (101 MHz, $CDCl_3$) δ 145.4, 144.8, 125.7, 119.6, 119.2, 112.7.

1-(*p*-methoxyphenyl)pyrrole (**8**)

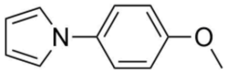

Prepared according to general procedure 1 and purified by flash chromatography (10-50% ethyl acetate in petroleum ether). The product was obtained in isolated yield as white solid (1.33 mmol, 0.231 g, 67% from 25 mL of 80mM solution). Spectral data are consistent with previously reported data in literature[2]: ^{1}H (400 MHz, $CDCl_3$) δ 7.31 (ddd, *J* = 8.9, 3.5, 2.2 Hz, 2H), 7.02 – 6.98 (m, 2H), 6.95 (ddd, *J* = 8.9, 3.5, 2.2 Hz, 2H), 6.34 – 6.30 (m, 2H), 3.84 (s, 3H). ^{13}C NMR (101 MHz, $CDCl_3$) δ 157.8, 134.6, 122.4, 119.8, 114.8, 110.0, 55.7.

4-(1*H*-pyrrole-1-yl)benzoic acid (**9**)

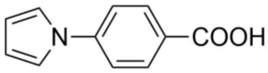

Prepared according to general procedure 1 and purified by liquid-liquid extraction (diethyl ether - water). The product was obtained in isolated yield as yellow solid (1.33 mmol, 0.231 g, 67% from 25 mL of 80mM solution). Prepared according to general procedure 2 (yield: 0.72 mmol, 0.134 g, 72%). Spectral data are consistent with previously reported data in literature [4]: ^{1}H NMR (400 MHz, $(CD_3)_2SO$) δ 7.99 (dd, *J* = 8.7, 1.7 Hz, 2H), 7.72 (dd, *J* = 8.7, 1.7 Hz, 2H), 7.53 – 7.47 (m, 2H), 6.33 – 6.29 (m, 2H). ^{13}C NMR (101 MHz, $(CD_3)_2SO$) δ 166.8, 143.1, 131.1, 127.1, 119.1, 118.6, 111.4.

1-(4-(trifluoromethyl)phenyl)-1*H*-pyrrole (**10**)

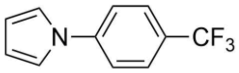

Prepared according to general procedure 1 and purified by flash chromatography (10-50% ethyl acetate in petroleum ether). The product was obtained in isolated yield as brown solid (0.468 mmol, 0.0988 g, 51% from 25 mL of 80mM solution). Spectral data are consistent with previously reported data in literature[5]: ^{1}H NMR (400 MHz, $CDCl_3$) δ 7.73 – 7.63 (m, 2H), 7.54 – 7.46 (m, 2H), 7.14 (dd, J = 2.2, 2.2 Hz, 2H), 6.40 (dd, J = 2.2, 2.2 Hz, 2H). ^{13}C NMR (101 MHz, $CDCl_3$) δ 143.3, 127.6 (d, J = 32.8 Hz), 127.0 (q, J = 3.8 Hz), 124.1 (d, J = 271.7 Hz), 120.1, 119.3, 111.6. ^{19}F NMR (376 MHz, $CDCl_3$) δ -62.09.

1-*o*-Tolyl-1*H*-pyrrole (**11**)

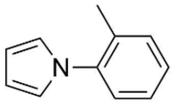

Prepared according to general procedure 1 and purified by liquid-liquid extraction (diethyl ether - water). The product was obtained in isolated yield as yellow oil (1.71 mmol, 0.268 g, 81% from 25 mL of 85mM solution). Prepared according to general procedure 2 (yield: 0.64 mmol, 0.100 g, 64%). Spectral data are consistent with previously reported data in literature[6]: ^{1}H NMR (400 MHz, $CDCl_3$) δ

7.31 – 7.26 (m, 2H), 7.25 (ddd, *J* = 4.5, 2.2, 1.0 Hz, 2H), 6.82 – 6.76 (m, 2H), 6.34 – 6.28 (m, 2H), 2.21 (s, 3H). ^{13}C NMR (101 MHz, $CDCl_3$) δ 140.7, 134.0, 131.2, 127.6, 126.8, 126.7, 122.2, 108.8, 18.0.

1-(2-isopropylphenyl)pyrrole (**12**)

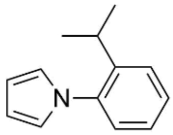

Prepared according to general procedure 1 and purified by liquid-liquid extraction (diethyl ether - water). The product was obtained in isolated yield as yellow oil (1.46 mmol, 0.270 g, 73% from 25 mL of 80 mM solution). ^{1}H NMR (400 MHz, $CDCl_3$) δ 7.44 – 7.34 (m, 2H), 7.24 – 7.21 (m, 2H), 6.79 – 6.76 (m, 2H), 6.33 – 6.30 (m, 2H), 2.84 (hept, *J* = 6.9 Hz, 1H), 1.18 (s, 3H), 1.16 (s, 3H). ^{13}C NMR (101 MHz, $CDCl_3$) δ 145.4, 139.4, 128.4, 127.5, 126.6, 126.2, 122.9, 108.7, 27.6, 24.4. HRMS m/z calculated for C13H16N [M$^+$] found 186.1284 calc. 186.1283.

1-(2-*tert*-butylphenyl)pyrrole (**13**)

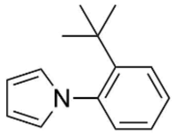

Prepared according to general procedure 1 (not isolated, ^{1}H NMR yield 65%). ^{1}H NMR (400 MHz, $CDCl_3$) δ 7.57 – 7.50 (m, 1H), 7.38 – 7.31 (m, 1H), 7.23 – 7.15 (m, 1H), 7.09 – 7.04 (m, 1H), 6.77 – 6.72 (m, 2H), 6.25 – 6.19 (m, 2H), 1.21 – 1.14 (m, 9H). ^{13}C NMR (101 MHz, $CDCl_3$) δ 140.2, 131.2, 128.6, 127.9, 126.3, 124.6, 107.9, 31.7. HRMS m/z calculated for C14H17N [M$^+$] found 199.1356 calc. 199.1348.

1-(5,6,7,8-tetrahydronaphthalen-1-yl)pyrrole (**14**)

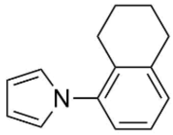

Prepared according to general procedure 1 and purified by liquid-liquid extraction (diethyl ether - water). The product was obtained in isolated yield as brown oil (1.31 mmol, 0.261 g, 65% from 25 mL of 80mM solution). Prepared according to general procedure 2 (yield: 0.95 mmol, 0.188 g, 95%). ^{1}H NMR (400 MHz, $CDCl_3$) δ 7.19 – 7.12 (m, 2H), 7.12 – 7.04 (m, 1H), 6.79 – 6.73 (m, 2H), 6.33 – 6.28 (m, 2H), 2.86 (dd, *J* = 6.3, 6.3 Hz, 2H), 2.51 (dd, *J* = 6.3, 6.3 Hz, 2H), 1.85 – 1.76 (m, 2H), 1.72 (dtt, *J* = 9.6, 6.2, 2.6 Hz, 2H). ^{13}C NMR (101 MHz, $CDCl_3$) δ 140.6, 138.8, 133.8, 128.9, 125.8, 124.2, 122.3, 108.5, 29.7, 25.2, 22.9, 22.9. HRMS m/z calculated for C14H15N [M$^+$] found 197.1197 calc. 197.1199.

N-benzoylpyrrole (**15**)

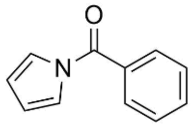

Prepared according to general procedure 1 (not isolated, ^{1}H NMR yield 38%). Prepared according to general procedure 2 and obtained in isolated yield as brown oil (0.90 mmol, 0.154 g, 89% yield). Spectral data are consistent with previously reported data in literature[7]: ^{1}H NMR (400 MHz, $(CD_3)_2SO$) δ 7.79 – 7.70 (m, 2H), 7.72 – 7.66 (m, 1H), 7.63 – 7.54 (m, 2H), 7.32 – 7.26 (m, 2H), 6.43 – 6.37 (m, 2H). ^{13}C NMR (101 MHz, $(CD_3)_2SO$) δ 167.2, 132.7, 132.5, 129.3, 128.7, 121.2, 113.4.

N-phenylsulfonylpyrrole (**16**)

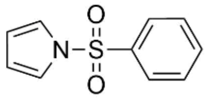

Prepared according to general procedure 1 and purified by liquid-liquid extraction (diethyl ether – water). The product was obtained in isolated yield as a dark solid (0.90 mmol, 0.287 g, 87% yield). Spectral data are consistent with previously reported data in literature[8]: ^{1}H NMR (400 MHz, $CDCl_3$) δ 7.88 – 7.83 (m, 2H), 7.63 – 7.57 (m, 1H), 7.53 – 7.47 (m, 2H), 7.19 – 7.14 (m, 2H), 6.33 – 6.27 (m, 2H). ^{13}C NMR (101 MHz, $CDCl_3$) δ 138.2, 134.6, 129.9, 126.7, 121.1, 114.0.

4-(1*H*-pyrrol-1-yl)phenol (**17**)

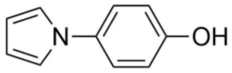

Prepared according to general procedure 2 and purified by liquid-liquid extraction (diethyl ether – water). The product was obtained in isolated yield as a white solid (0.69 mmol, 0.11 g, 69% yield). Spectral data are consistent with previously reported data in literature[9]: ^{1}H NMR (400 MHz, $(CD_3)_2SO$) δ 9.49 (s, 1H), 7.37 – 7.28 (m, 2H), 7.16 (dd, *J* = 2.2, 2.2 Hz, 2H), 6.86 – 6.78 (m, 2H), 6.19 (dd, *J* = 2.1, 2.1 Hz, 2H). ^{13}C NMR (101 MHz, $(CD_3)_2SO$) δ 157.3, 129.7, 125.3, 119.3, 118.9, 110.5.

methyl 4-(1*H*-pyrrol-1-yl)benzoate (**18**)

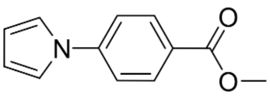

Prepared according to general procedure 2 and purified by liquid-liquid extraction (diethyl ether – water). The product was obtained in isolated yield as yellow solid (0.71 mmol, 0.14 g, 71%). Spectral data are consistent with previously reported data in literature[10]: ^{1}H NMR (400 MHz, $(CD_3)_2SO$) δ 8.01 (d, J = 8.9 Hz, 2H), 7.75 (d, J = 8.9 Hz, 2H), 7.52 (dd, J = 2.2, 2.2 Hz, 2H), 6.33 (dd, J = 2.2, 2.2 Hz, 2H), 3.86 (s, 3H). ^{13}C NMR (101 MHz, $(CD_3)_2SO$) δ 165.7, 143.3, 130.9, 125.9, 119.1, 118.6, 111.5, 52.1.

(4-pyrrol-1-ylphenyl)sulfonamide (**19**)

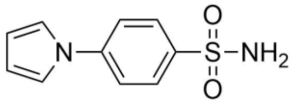

Prepared according to general procedure 2 and purified by liquid-liquid extraction (diethyl ether – water). The product was obtained in isolated yield as a white solid (0.72 mmol, 0.16 g, 72% yield). Spectral data are consistent with previously reported data in literature[11]: ^{1}H NMR (400 MHz, $(CD_3)_2SO$) δ 7.87 (AA', J = 9.1 Hz, 2H), 7.79 (BB', J = 9.1 Hz, 2H), 7.49 (dd, J = 2.2, 2.2 Hz, 2H), 7.39 (s, 2H), 6.32 (dd, J = 2.2, 2.2 Hz, 2H). ^{13}C NMR (101 MHz, $(CD_3)_2SO$) δ 142.1, 140.4, 127.4, 119.2, 119.0, 111.4.

1-(2,6-dimethylphenyl)-1*H*-pyrrole (**20**)

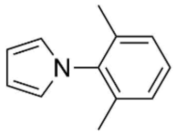

Prepared according to general procedure 2 and purified by liquid-liquid extraction (diethyl ether – water). The product was obtained in isolated yield as a clear liquid (0.61 mmol, 0.10 g, 61% yield). Spectral data are consistent with previously reported data in literature[12]: ^{1}H NMR (400 MHz, $(CD_3)_2SO$) δ 7.24 (ddd, J = 8.1, 6.4, 1.5 Hz, 1H), 7.17 (d, J = 8.1 Hz, 2H), 6.71 (dd, J = 4.3, 2.0 Hz, 2H), 6.23 (dd, J = 4.3, 2.0 Hz, 2H), 1.98 – 1.94 (m, 6H). ^{13}C NMR (101 MHz, $(CD_3)_2SO$) δ 139.6, 135.4, 128.0, 128.0, 121.4, 108.5, 17.0.

3-(1-pyrrolyl)benzoic acid (**21**)

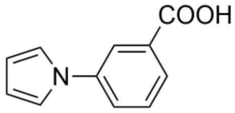

Prepared according to general procedure 2 and purified by liquid-liquid extraction (diethyl ether – water). The product was obtained in isolated yield as a white solid (0.38 mmol, 0.07 g, 38% yield). Spectral data are consistent with previously reported data in literature[13]: ^{1}H NMR (400 MHz, $(CD_3)_2SO$) δ 8.02 (ddd, J = 2.6, 1.5, 0.6 Hz, 1H), 7.84 (dddd, J = 8.1, 2.5, 1.1, 0.6 Hz, 1H), 7.82 – 7.77 (m, 1H), 7.59 (dd, J = 7.9, 7.9 Hz, 1H), 7.43 (dd, J = 2.2, 2.2 Hz, 2H), 6.29 (dd, J = 2.2, 2.2 Hz, 2H). ^{13}C NMR (101 MHz, $(CD_3)_2SO$) δ 166.8, 140.1, 132.4, 130.1, 126.0, 123.7, 119.6, 119.1, 110.9.

5-(1*H*-pyrrol-1-yl)benzene-1,3-dicarboxylic acid (**22**)

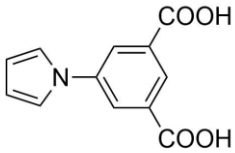

Prepared according to general procedure 2 and purified by liquid-liquid extraction (diethyl ether – water). The product was obtained in isolated yield as a white solid (0.99 mmol, 0.23 g, 99% yield).

Spectral data are consistent with previously reported data in literature:[14] ^{1}H NMR (400 MHz, $(CD_3)_2SO$) δ 8.31 (s, 1H), 8.23 (dd, *J* = 1.4, 1.4 Hz, 2H), 7.51 (dd, *J* = 2.2, 2.2 Hz, 2H), 6.32 (dd, *J* = 2.2, 2.2 Hz, 2H). ^{13}C NMR (101 MHz, $(CD_3)_2SO$) δ 166.2, 140.5, 133.0, 126.4, 123.6, 119.4, 111.4.

N-(3-(1H-tetrazol-5-yl)phenyl)pyrrole (**23**)

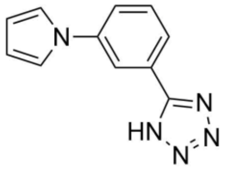

Prepared according to general procedure 2 and purified by liquid-liquid extraction (diethyl ether – water). The product was obtained in isolated yield as a white solid (0.74mmol, 0.15 g, 74% yield). Spectral data are consistent with previously reported data in literature[13]: ^{1}H NMR (400 MHz, $(CD_3)_2SO$) δ 8.19 (dd, *J* = 2.0, 2.0 Hz, 1H), 7.90 (ddd, *J* = 7.9, 1.6, 1.1 Hz, 1H), 7.82 (ddd, *J* = 7.9, 2.4, 1.1 Hz, 1H), 7.69 (dd, *J* = 7.9, 7.9 Hz, 1H), 7.46 (dd, *J* = 2.2, 2.2 Hz, 2H), 6.33 (dd, *J* = 2.2, 2.2 Hz, 2H). ^{13}C NMR (101 MHz, $(CD_3)_2SO$) δ 154.9, 140.6, 131.0, 125.6, 123.7, 121.9, 119.1, 117.6, 111.1.

1-methylsulfonylpyrrole (**24**)

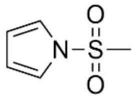

Prepared according to general procedure 2 and purified by liquid-liquid extraction (diethyl ether – water). The product was obtained in isolated yield as a brown liquid (0.81 mmol, 0.12 g, 81% yield). Spectral data are consistent with previously reported data in literature[15]: ^{1}H NMR (400 MHz, $(CD_3)_2SO$) δ 7.23 (dd, *J* = 2.3, 2.3 Hz, 2H), 6.38 (dd, *J* = 2.3, 2.3 Hz, 2H), 3.44 (s, 3H). ^{13}C NMR (101 MHz, $(CD_3)_2SO$) δ 120.5, 112.7, 42.3.

1-(benzylsulfonyl)-1*H*-pyrrole (**25**)

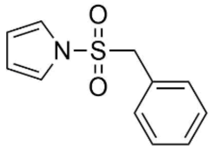

Prepared according to general procedure 2 and purified by liquid-liquid extraction (diethyl ether – water). The product was obtained in isolated yield as a brown solid (0.89 mmol, 0.20 g, 89% yield).Spectral data are consistent with previously reported data in literature[16]: ^{1}H NMR (400 MHz, $(CD_3)_2SO$) δ 7.39 – 7.26 (m, 3H), 7.10 – 7.03 (m, 2H), 6.95 (dd, *J* = 2.3, 2.3 Hz, 2H), 6.29 (dd, *J* = 2.3, 2.3 Hz, 2H), 4.95 (s, 2H). ^{13}C NMR (101 MHz, $(CD_3)_2SO$) δ 130.5, 128.9, 128.5, 127.9, 121.1, 112.4, 59.6. HRMS expected ion not found.

1-(4-carboxyphenylsulfonyl)-1*H*-pyrrole (**26**)

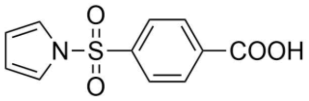

Prepared according to general procedure 2 and purified by liquid-liquid extraction (diethyl ether – water). The product was obtained in isolated yield as a yellow solid (0.38 mmol, 0.10 g, 38% yield).Spectral data are consistent with previously reported data in literature[17]: ^{1}H NMR (400 MHz, $(CD_3)_2SO$) δ 8.15 – 8.11 (m, 2H), 8.07 (dp, *J* = 8.7, 2.1 Hz, 2H), 7.37 (dd, *J* = 2.3, 2.3 Hz, 2H), 6.39 (dd, *J* = 2.3, 2.3 Hz, 2H). ^{13}C NMR (101 MHz, $(CD_3)_2SO$) δ 165.8, 141.5, 136.0, 130.6, 127.1, 121.3, 114.4.

1,5-Bis-(pyrrol-1'-yl)-naphthalin (**27**)

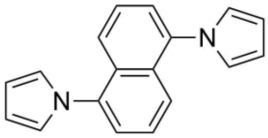

Prepared according to general procedure 2 and purified by liquid-liquid extraction (diethyl ether – water). The product was obtained in isolated yield as a yellow solid (0.87 mmol, 0.22 g, 87% yield).Spectral data are consistent with previously reported data in literature[18]: ^{1}H NMR (400 MHz, $(CD_3)_2SO$) δ 7.66 – 7.62 (m, 4H), 7.61 (dd, *J* = 5.4, 3.3 Hz, 2H), 7.13 (dd, *J* = 2.1, 2.1 Hz, 4H), 6.36 (dd, *J* = 2.1, 2.1 Hz, 4H). ^{13}C NMR (101 MHz, $(CD_3)_2SO$) δ 135.6, 130.1, 126.9, 124.1, 123.3, 122.5, 109.3.

1-(pyren-1-yl)-1*H*-pyrrole (**28**)

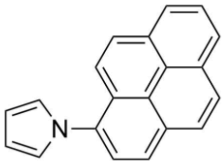

Prepared according to general procedure 2 and purified by liquid-liquid extraction (diethyl ether – water). The product was obtained in isolated yield as a red liquid (0.92 mmol, 0.25 g, 92% yield). Spectral data are consistent with previously reported data in literature[19]: ^{1}H NMR (400 MHz, $(CD_3)_2SO$) δ 8.45 – 8.30 (m, 3H), 8.29 – 8.20 (m, 3H), 8.13 (dd, *J* = 7.6, 7.6 Hz, 1H), 8.07 (d, *J* = 8.1 Hz, 1H), 7.90 (d, *J* = 9.3 Hz, 1H), 7.26 (dd, *J* = 2.1, 2.1 Hz, 2H), 6.43 (dd, *J* = 2.1, 2.1 Hz, 2H). ^{13}C NMR (101 MHz, $(CD_3)_2SO$) δ 134.9, 130.9, 130.4, 130.0, 128.7, 127.8, 127.2, 126.9, 125.9, 125.6, 125.5, 125.3, 124.4, 124.3, 123.7, 121.7, 109.5.

1-anthracen-2-ylpyrrole (**29**)

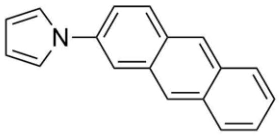

Prepared according to general procedure 2 and purified by liquid-liquid extraction (diethyl ether – water). The product was obtained in isolated yield as a black solid (0.87 mmol, 0.21 g, 87% yield). ^{1}H NMR (400 MHz, $(CD_3)_2SO$) δ 8.61 (s, 1H), 8.56 (s, 1H), 8.22 (dd, *J* = 5.8, 3.4 Hz, 2H), 8.12 – 8.04 (m, 2H), 7.87 (dd, *J* = 9.2, 2.2 Hz, 1H), 7.58 (dd, *J* = 2.2, 2.2 Hz, 2H), 7.56 – 7.48 (m, 2H), 6.35 (dd, *J* = 2.2, 2.2 Hz, 2H). ^{13}C NMR (101 MHz, $(CD_3)_2SO$) δ 136.8, 131.9, 131.4, 130.9, 130.1, 129.2, 128.2, 127.8, 126.3, 126.1, 125.6, 125.5, 120.0, 119.3, 114.8, 110.9.

5-(1*H*-pyrrol-1-yl)-1,10-phenanthroline (**30**)

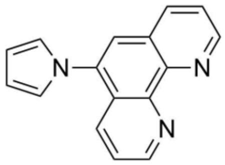

Prepared according to general procedure 2 and purified by liquid-liquid extraction (diethyl ether – water). The product was and purified by liquid-liquid extraction (diethyl ether – water). The product was and purified by liquid-liquid extraction (diethyl ether – water). The product was obtained in isolated yield as a yellow solid (0.10 mmol, 0.02 g, 10% yield). Spectral data are consistent with previously reported data in literature[20]: ^{1}H NMR (400 MHz, DMSO-D_6) δ 9.19 – 9.11 (m, 2H), 8.54 (dd, *J* = 8.4, 1.7 Hz, 1H), 8.08 (s, 1H), 8.06 (dd, *J* = 8.4, 1.7 Hz, 1H), 7.86 – 7.77 (m, 2H), 7.23 (dd, *J* = 2.1, 2.1 Hz, 2H), 6.40 (dd, *J* = 2.1, 2.1 Hz, 2H).

Cyclic voltammograms curves of the deposited films

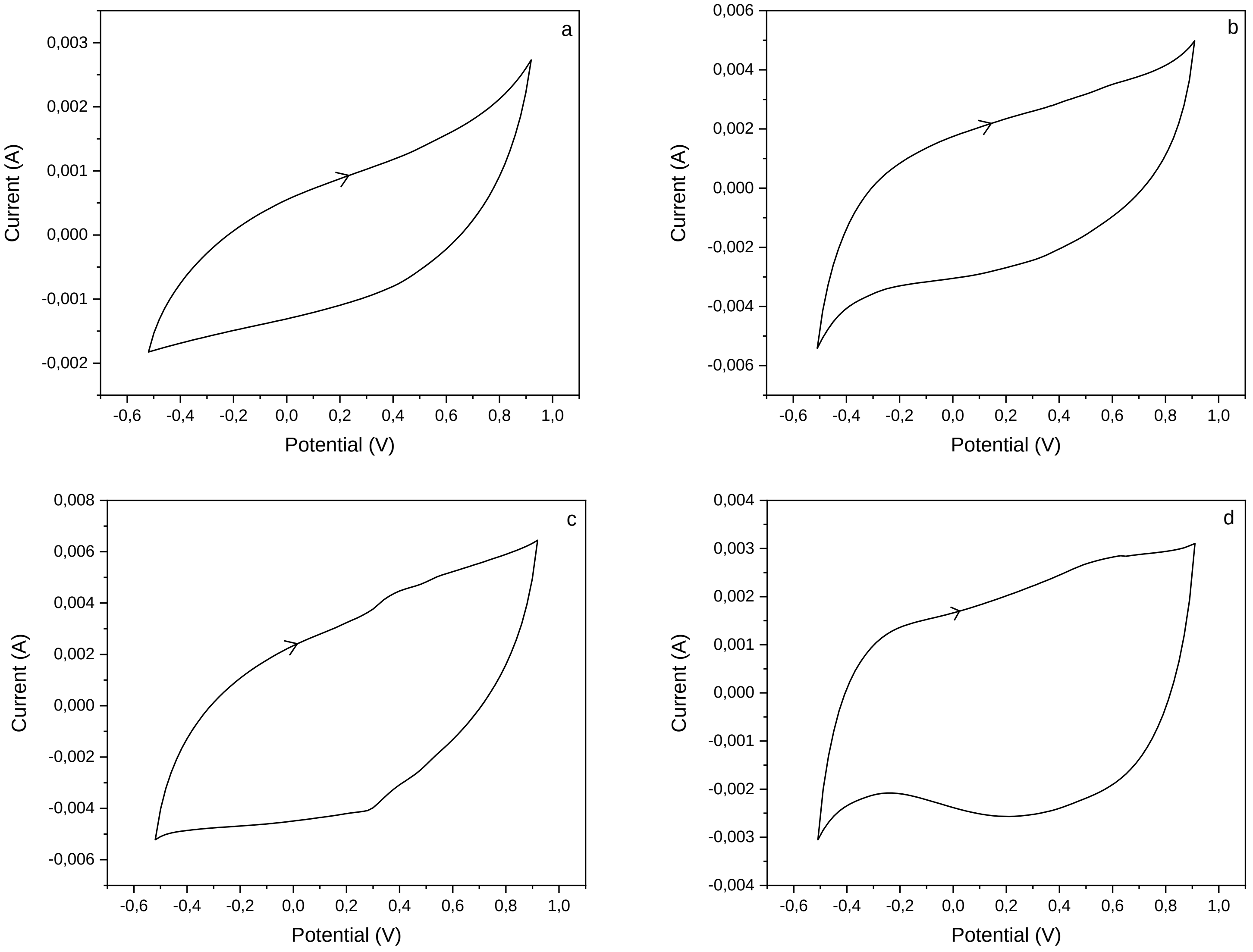

Figure S3. Cyclic voltammetry curves of the PPy/graphite paper in 0.5 M H_2SO_4 at a scan rate of 20 mV s^{-1}. Compound **3** (a), compound **25** (b), compound **29** (c), graphite paper (d).

FTIR spectra

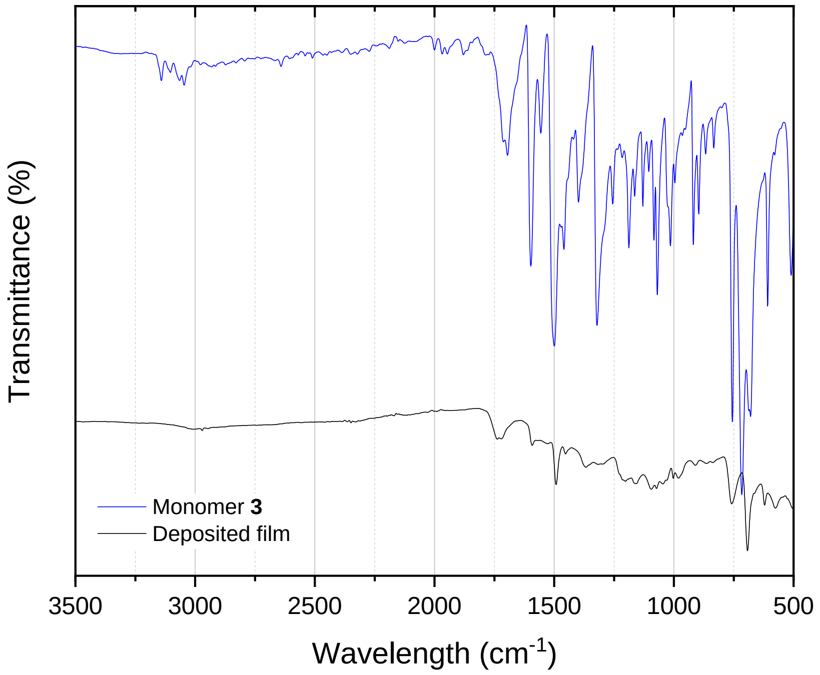

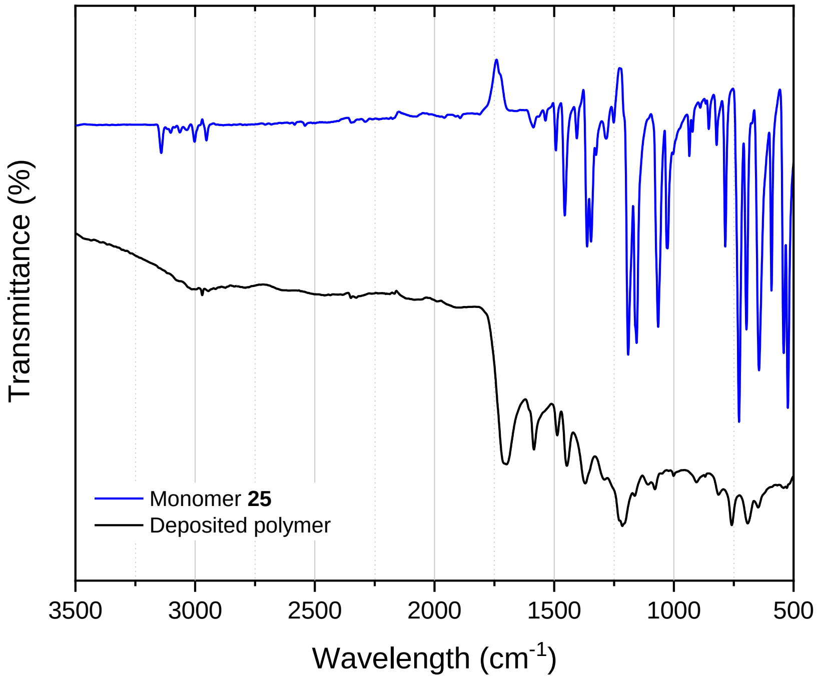

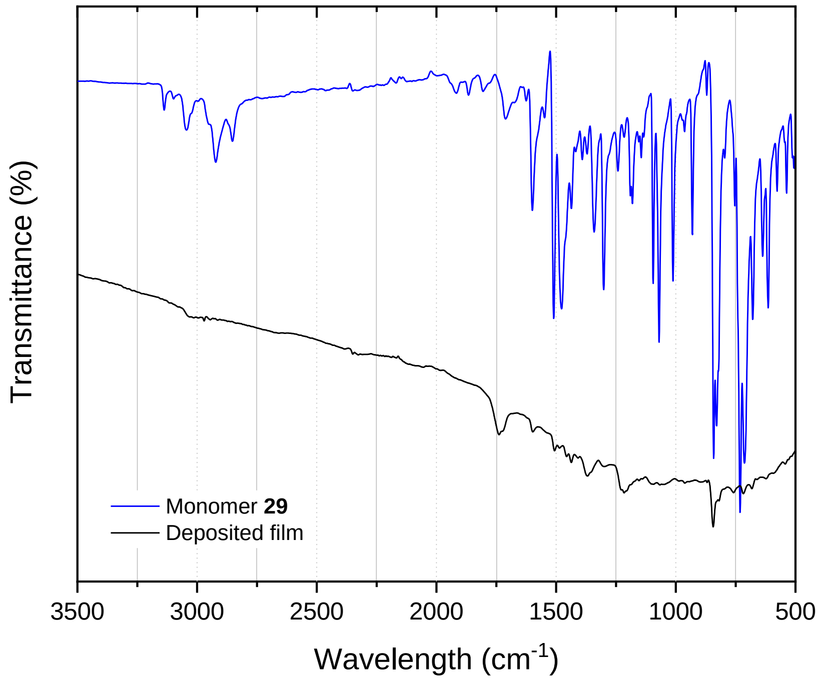

Figure S4. Full FT-IR spectra of compounds **3**, **25**, **29** and corresponding deposited films on graphite paper.

^{1}H NMR and ^{13}C NMR Spectra of products

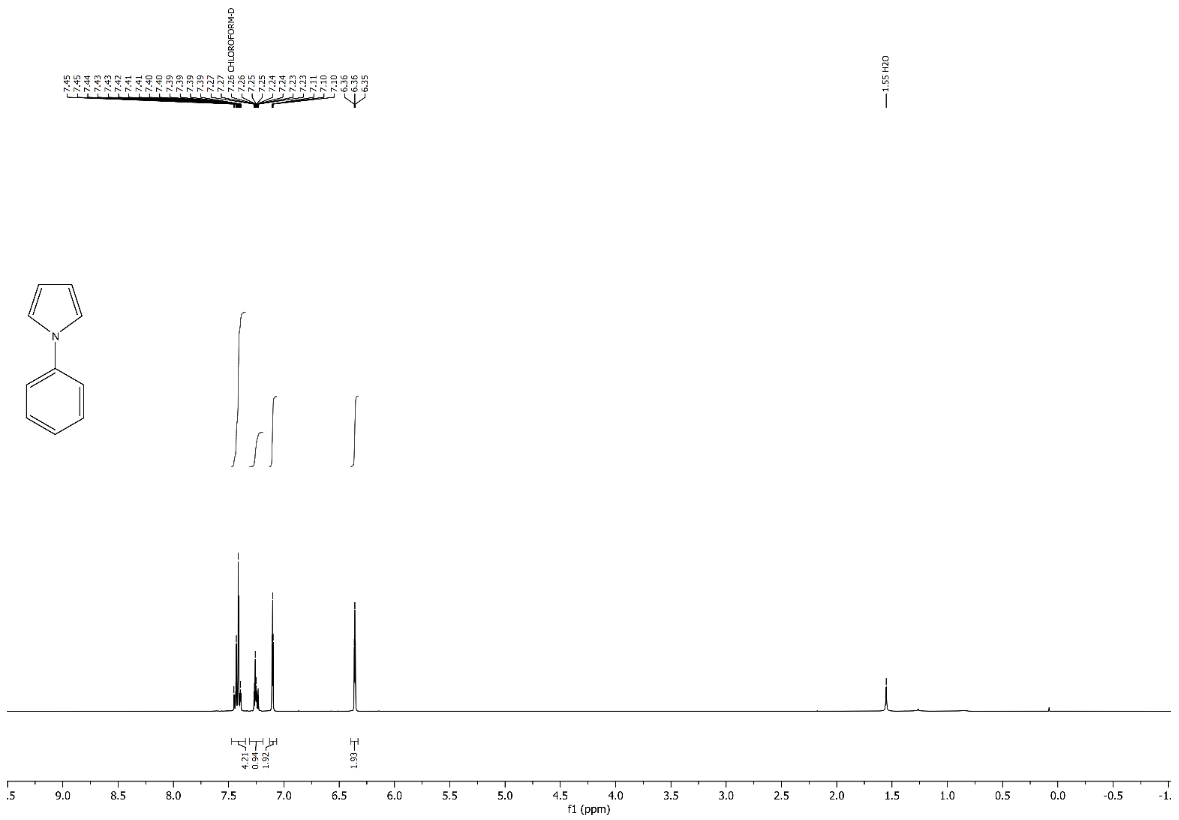

Figure S5. ^{1}H NMR spectrum of compound **3.**

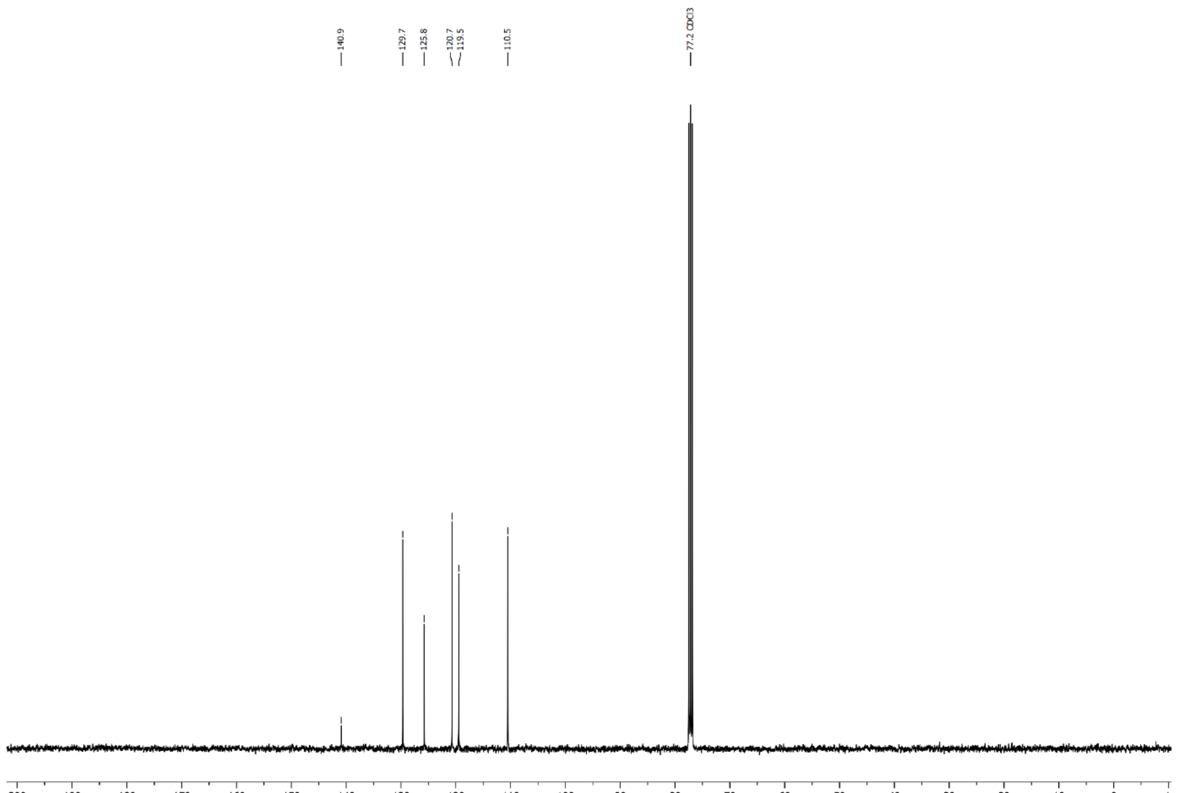

Figure S6. ^{13}C NMR spectrum of compound **3.**

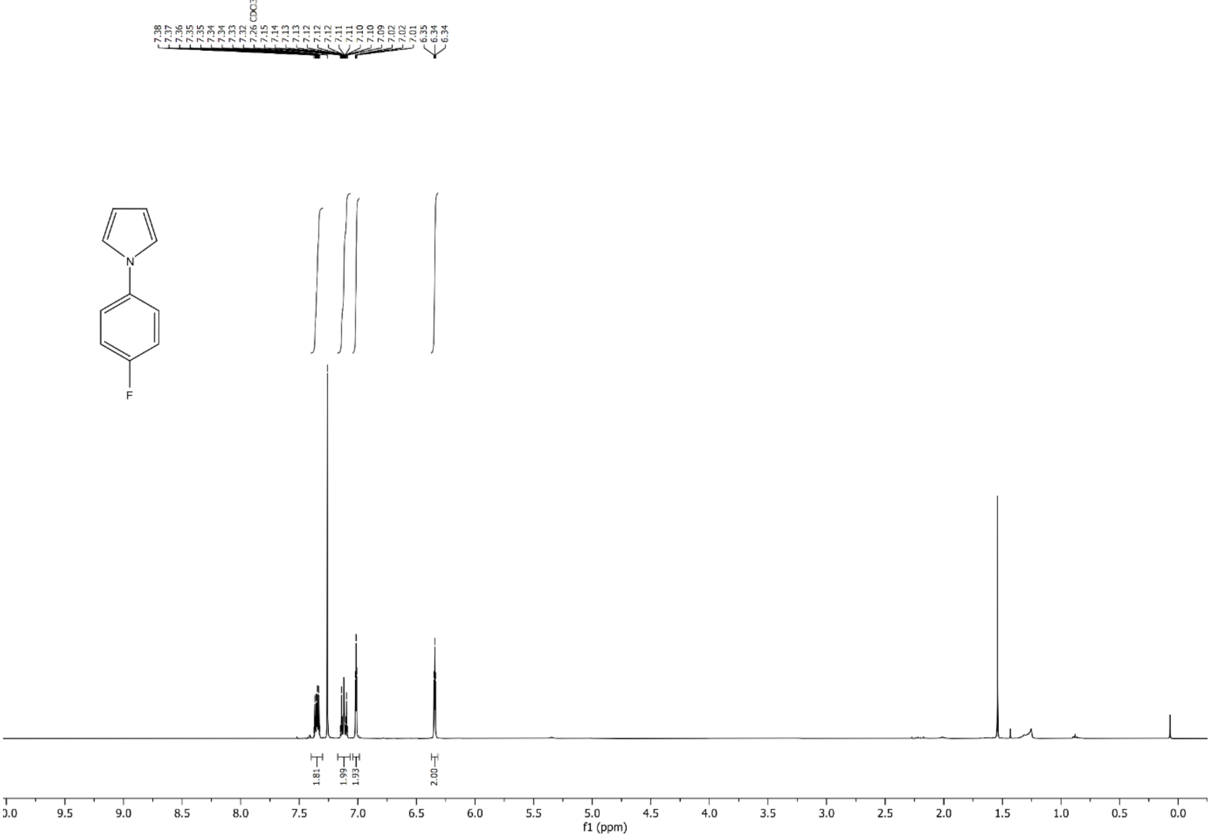

Figure S7. ^{1}H NMR spectrum of compound **4.**

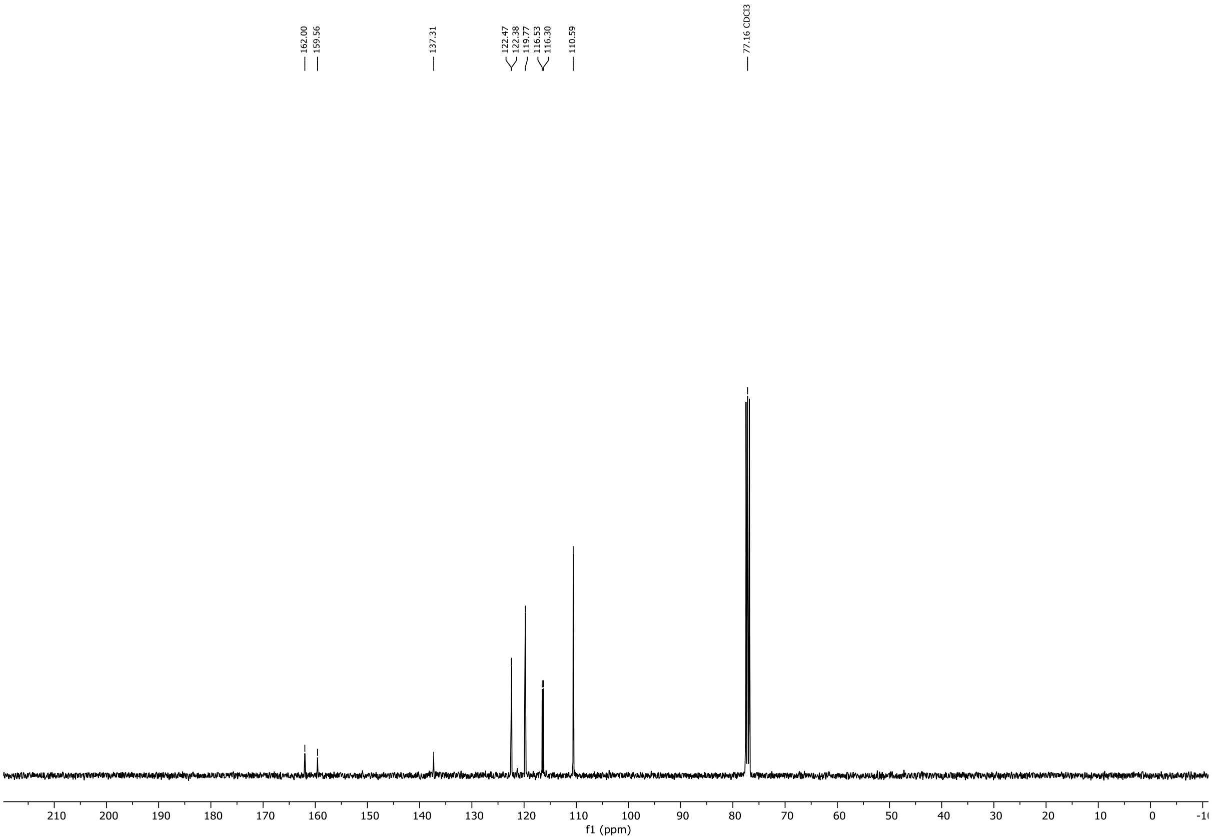

Figure S8. ^{13}C NMR spectrum of compound **4.**

C

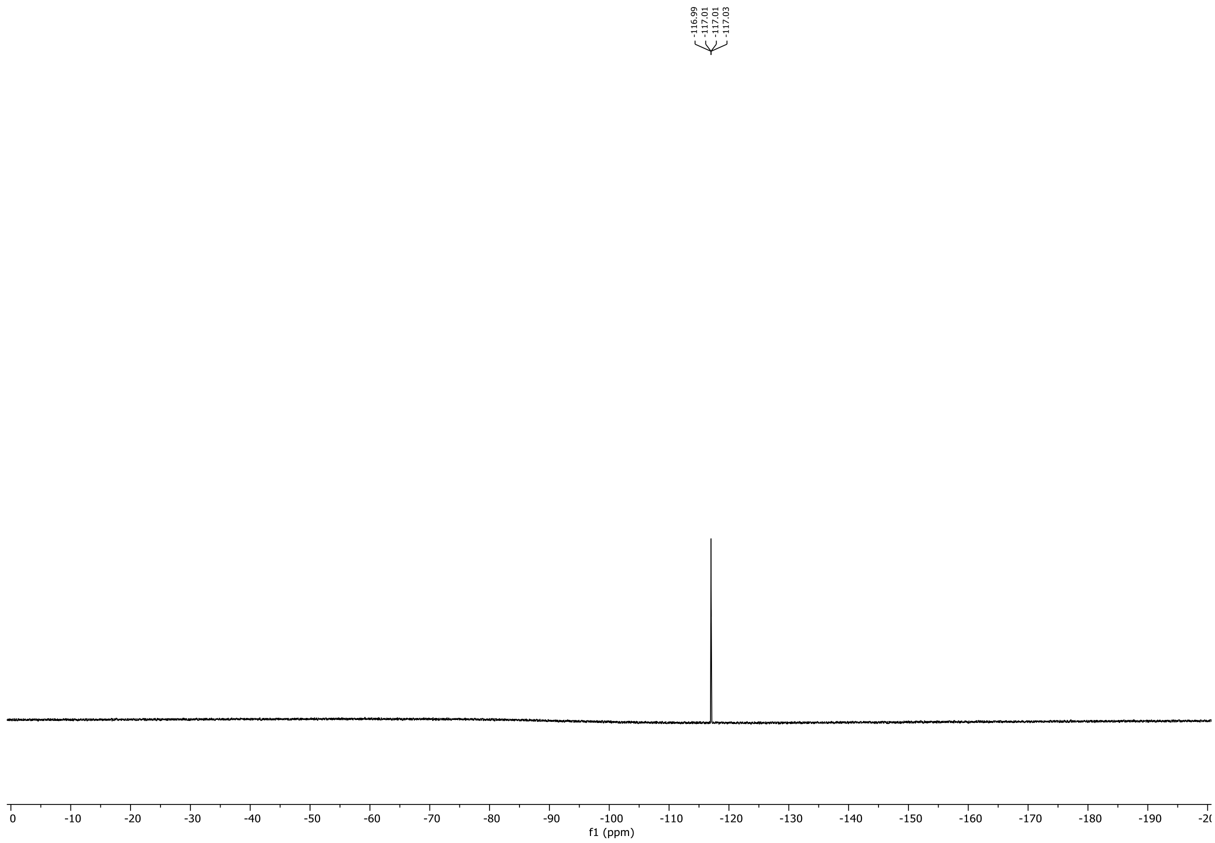

Figure S9. ^{19}F NMR spectrum of compound **4**.

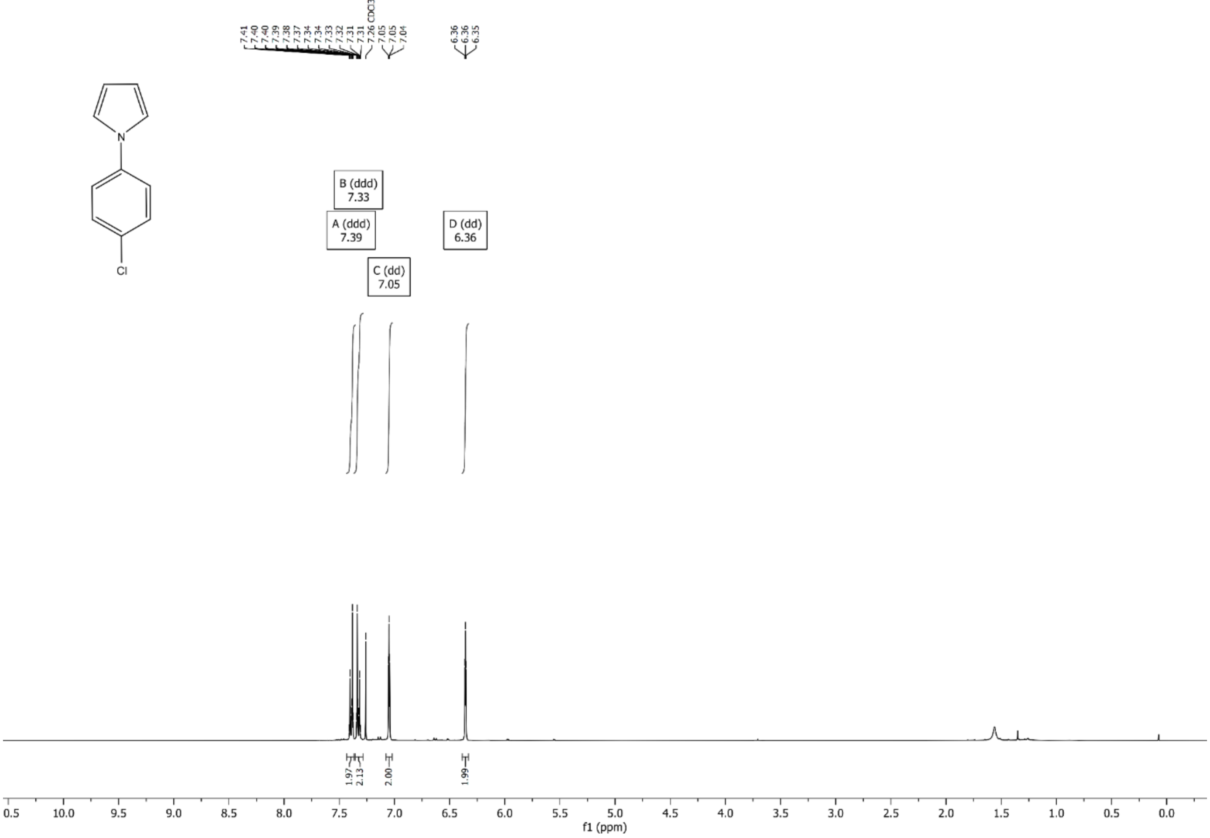

Figure S10. ^{1}H NMR spectrum of compound **5.**

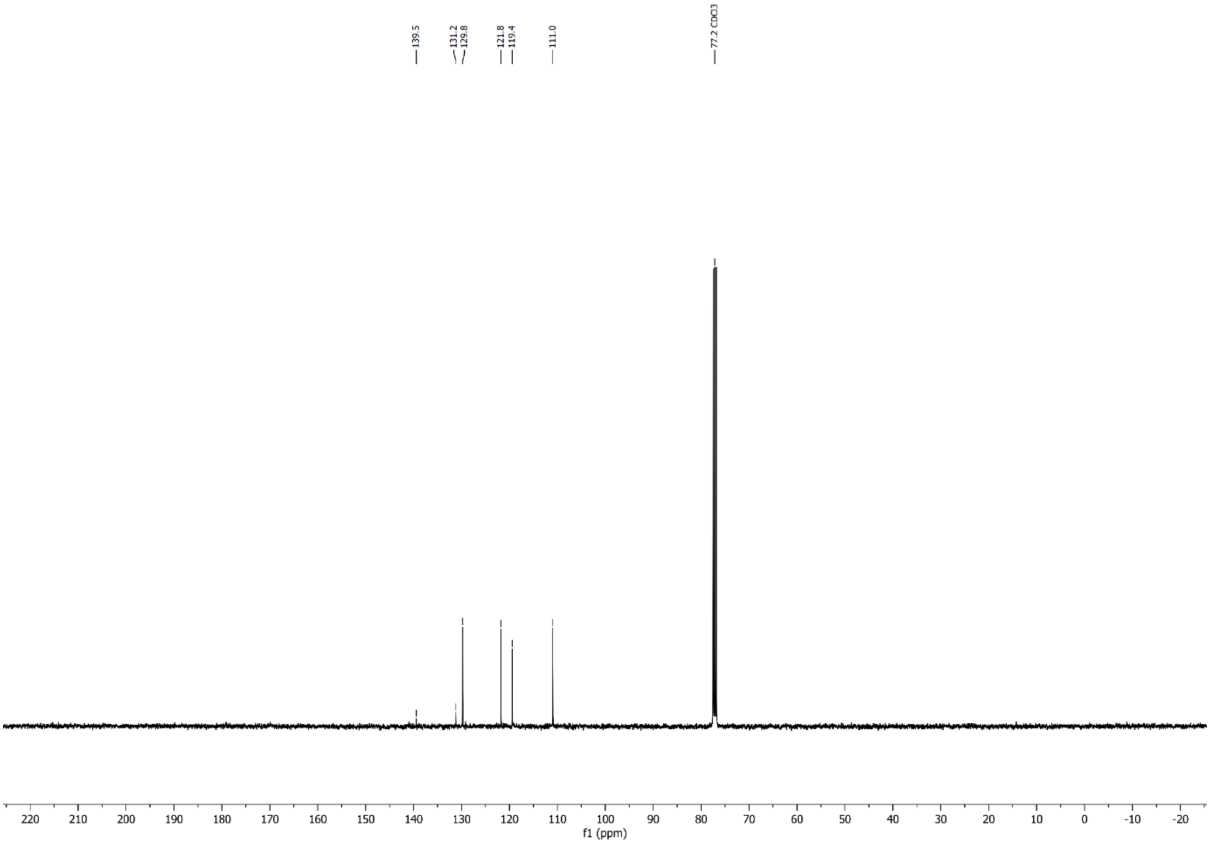

Figure S11. ^{13}C NMR spectrum of compound **5.**

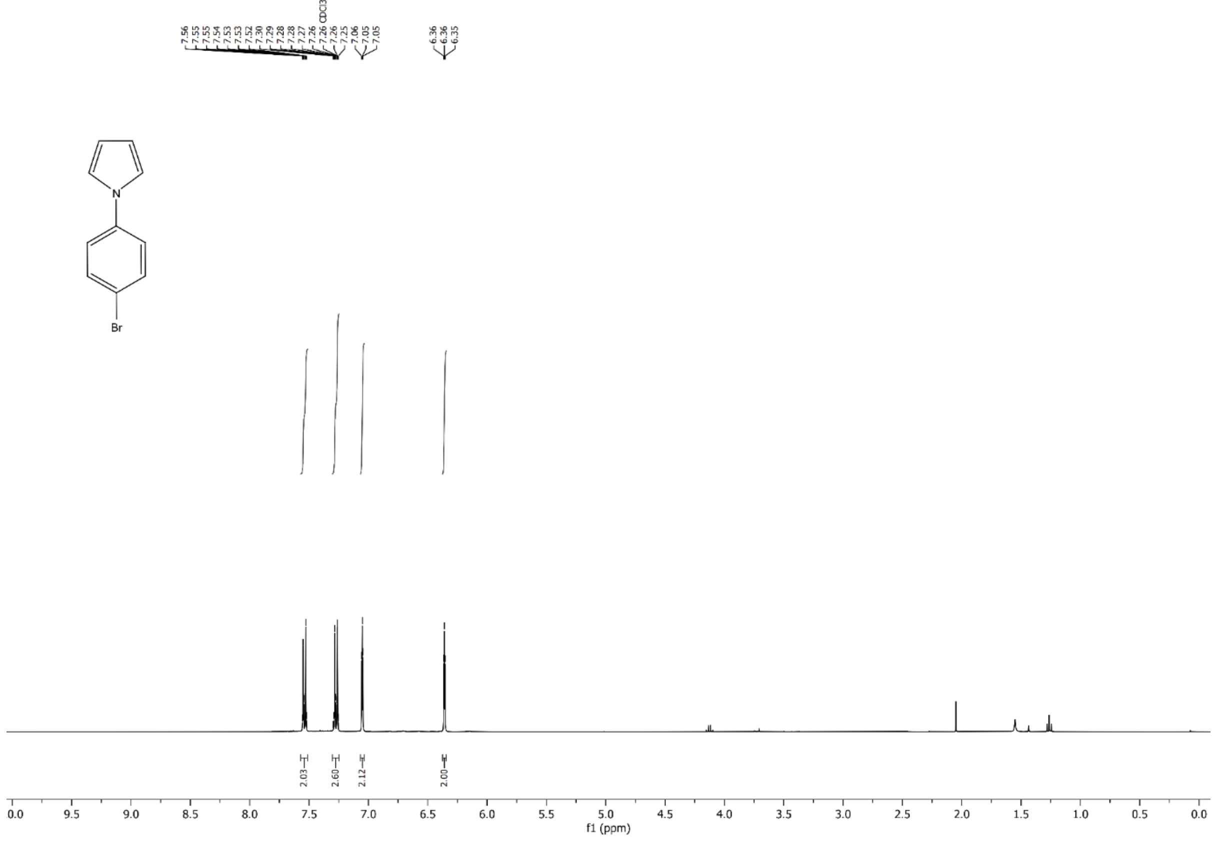

Figure S12. ^{1}H NMR spectrum of compound **6.**

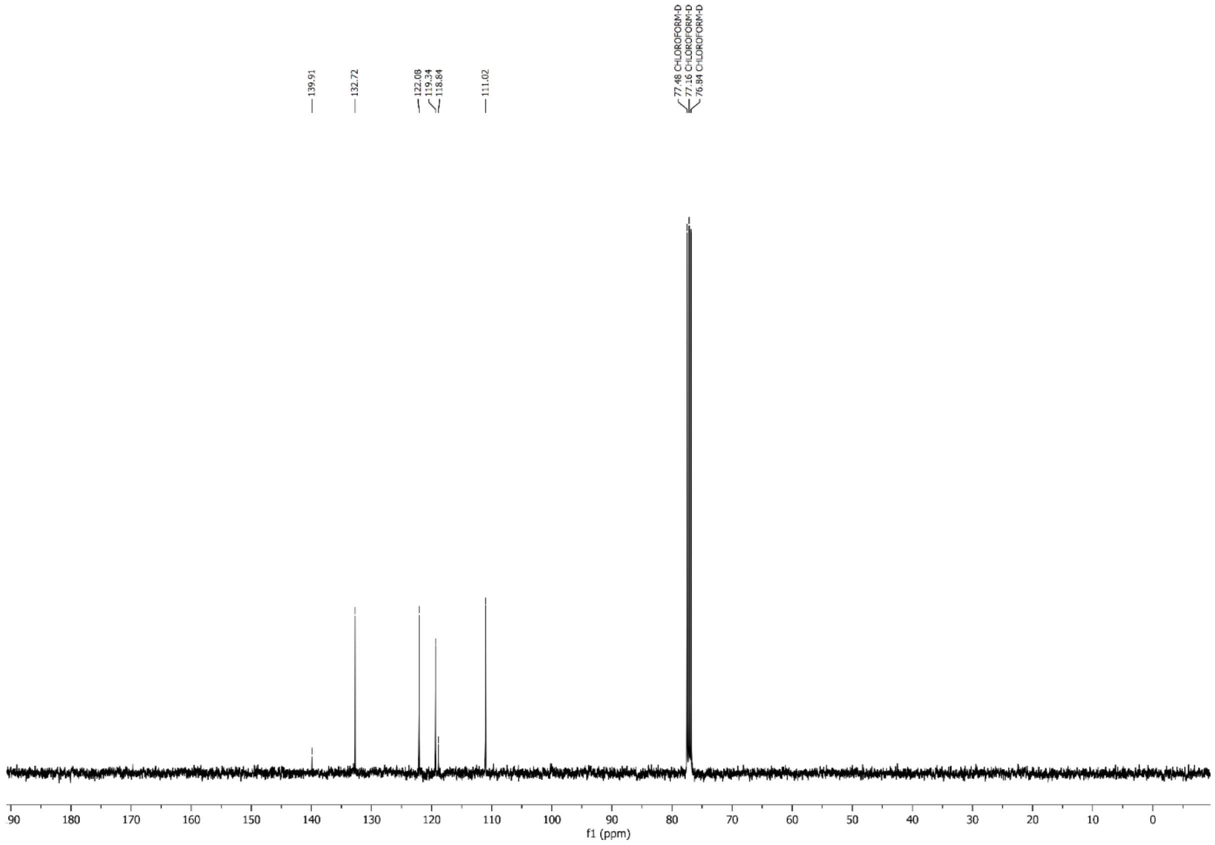

Figure S13. ^{13}C NMR spectrum of compound **6**.

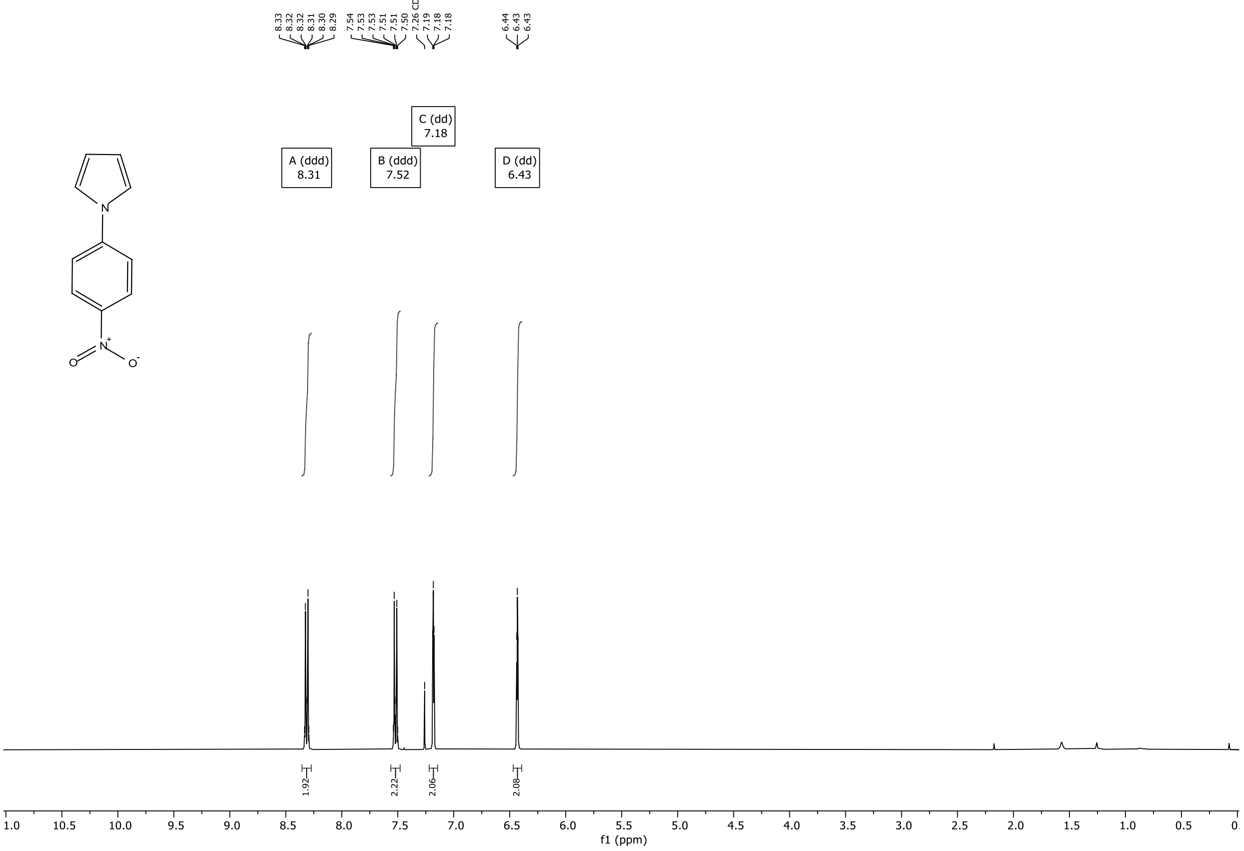

Figure S14. ^{1}H NMR spectrum of compound **7**.

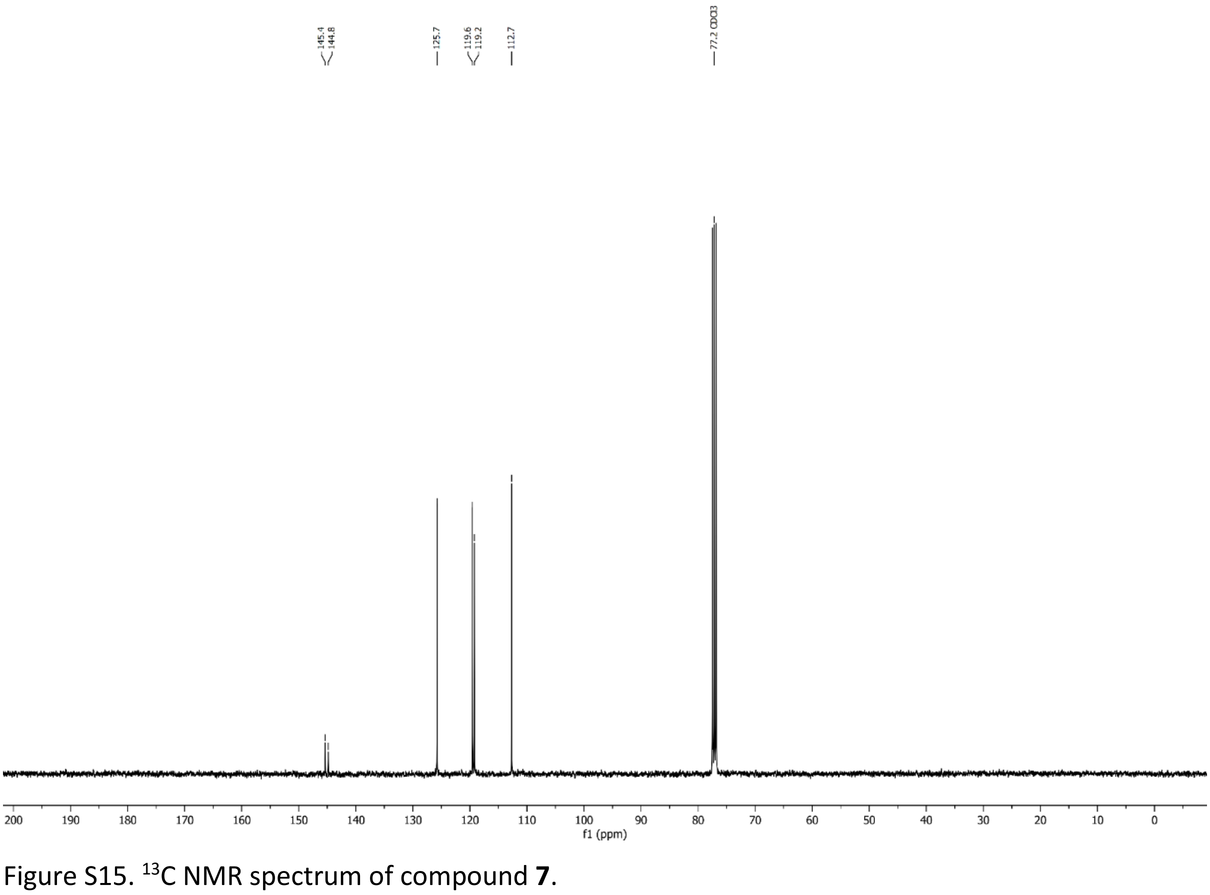

Figure S15. ^{13}C NMR spectrum of compound **7**.

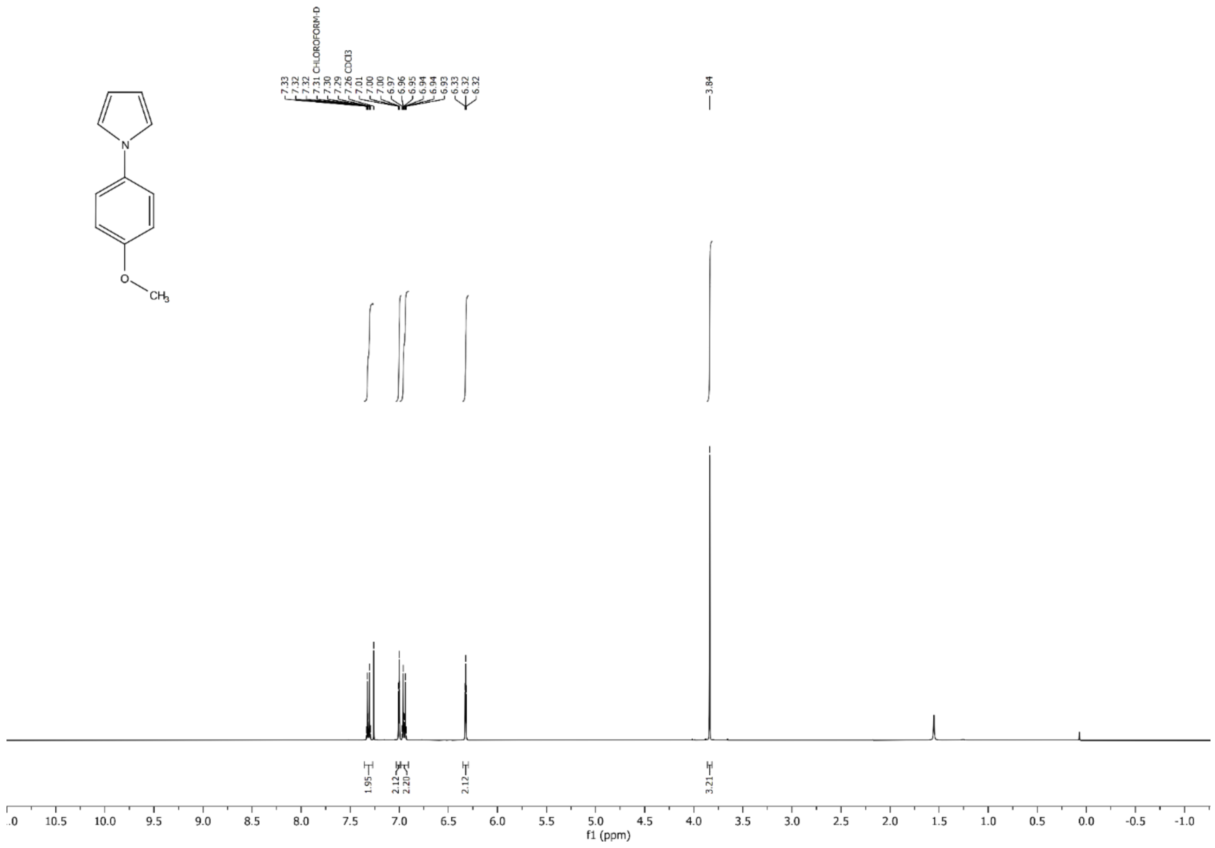

Figure S16. ^{1}H NMR spectrum of compound **8**.

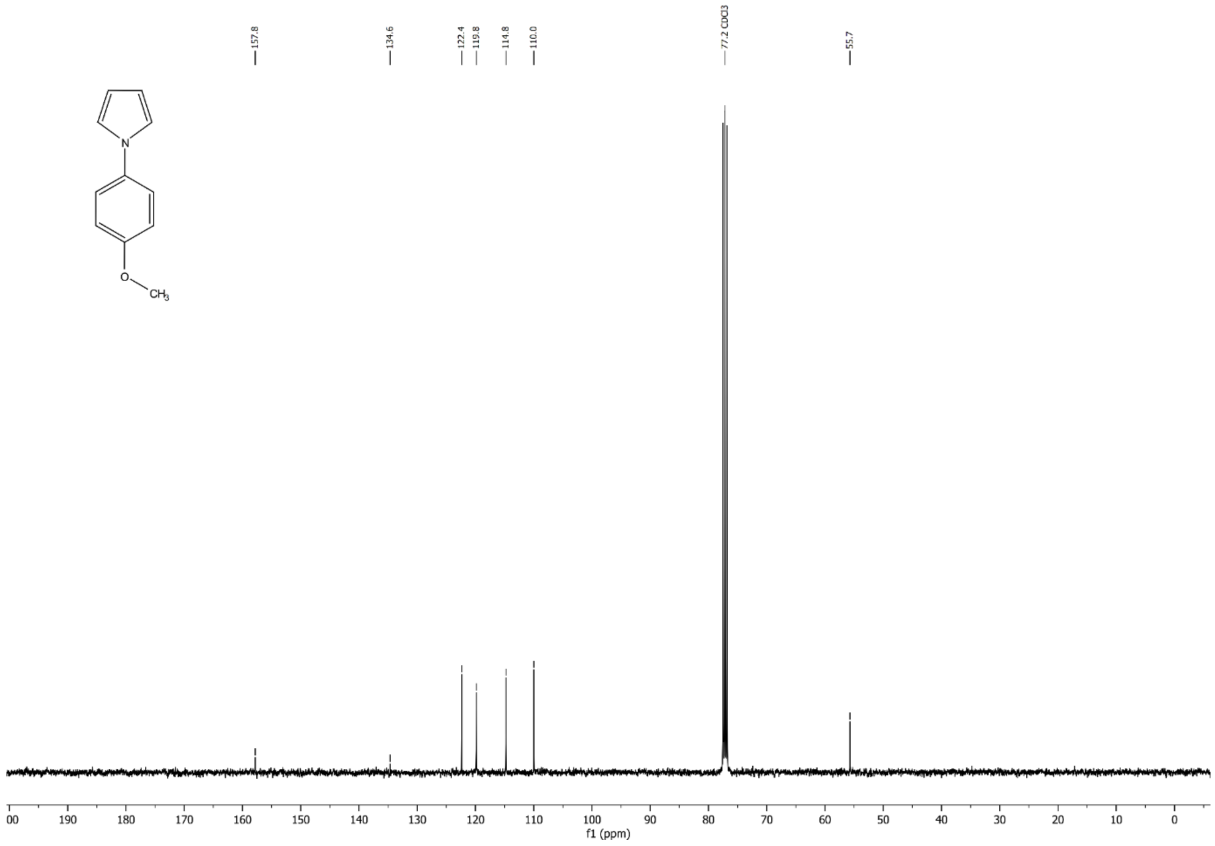

Figure S17. ^{13}C NMR spectrum of compound **8**.

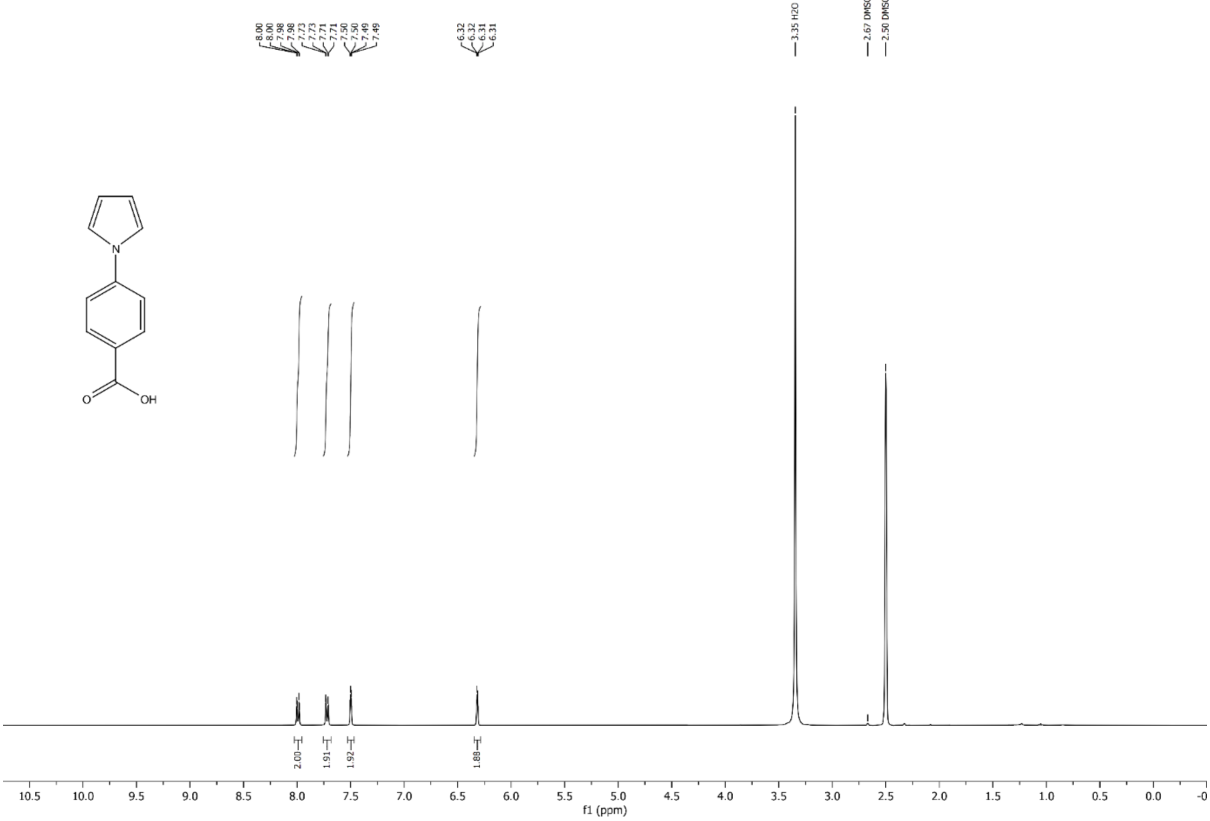

Figure S18. ^{1}H NMR spectrum of compound **9**.

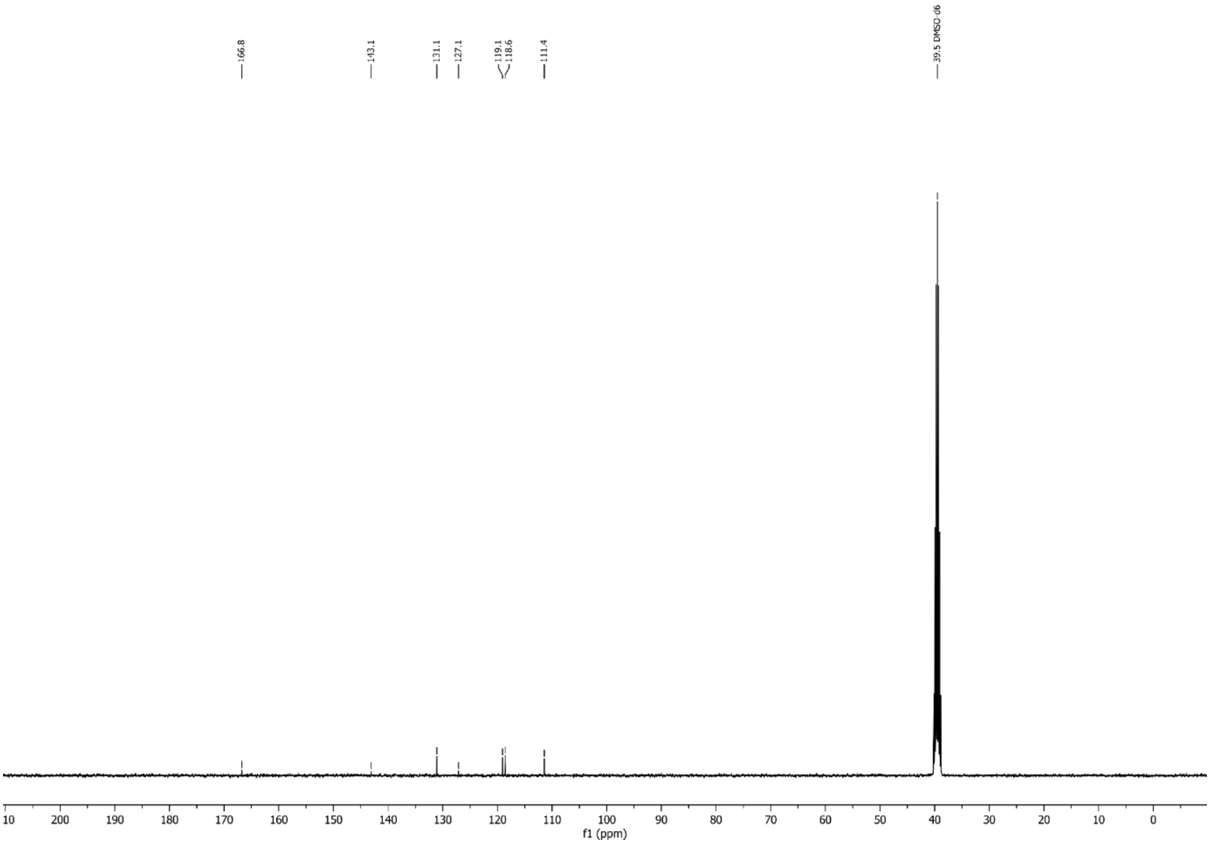

Figure S19. ^{13}C NMR spectrum of compound **9**.

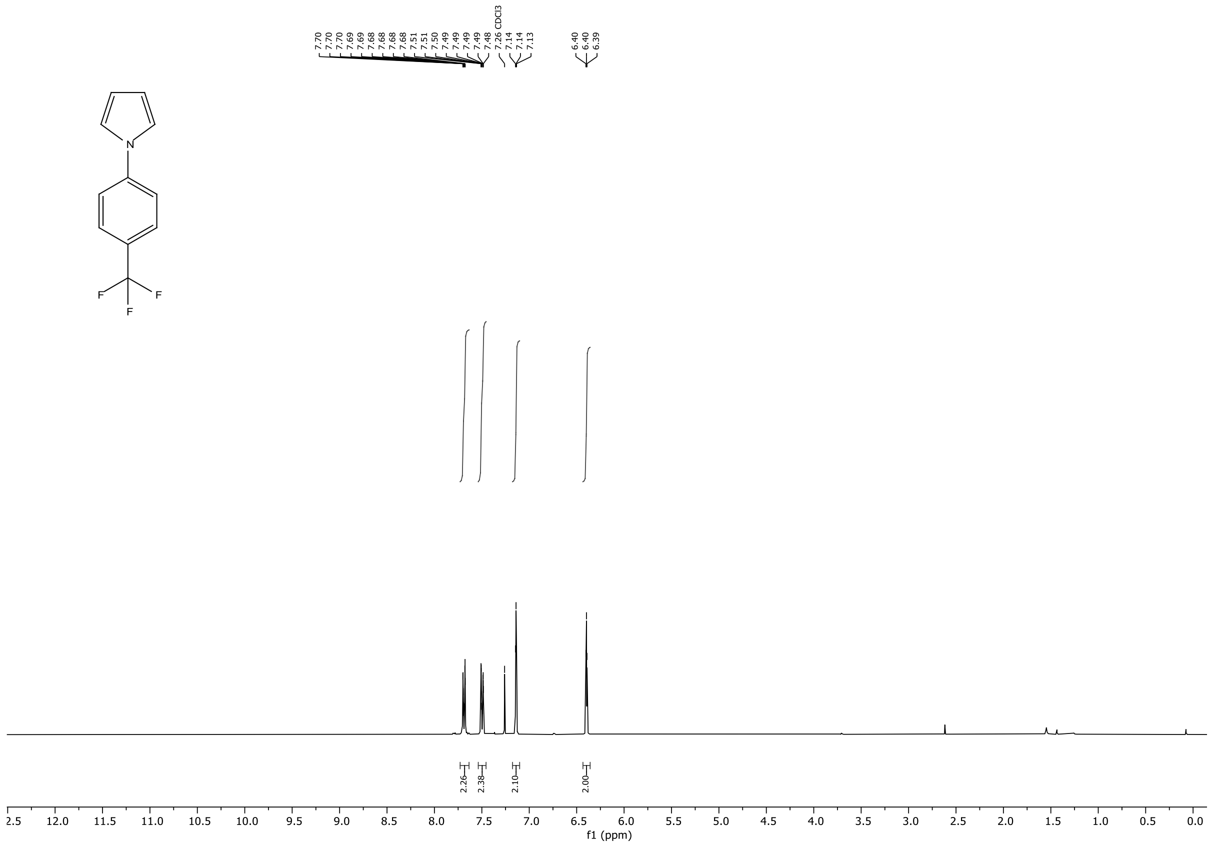

Figure S20. ^{1}H NMR spectrum of compound **10**.

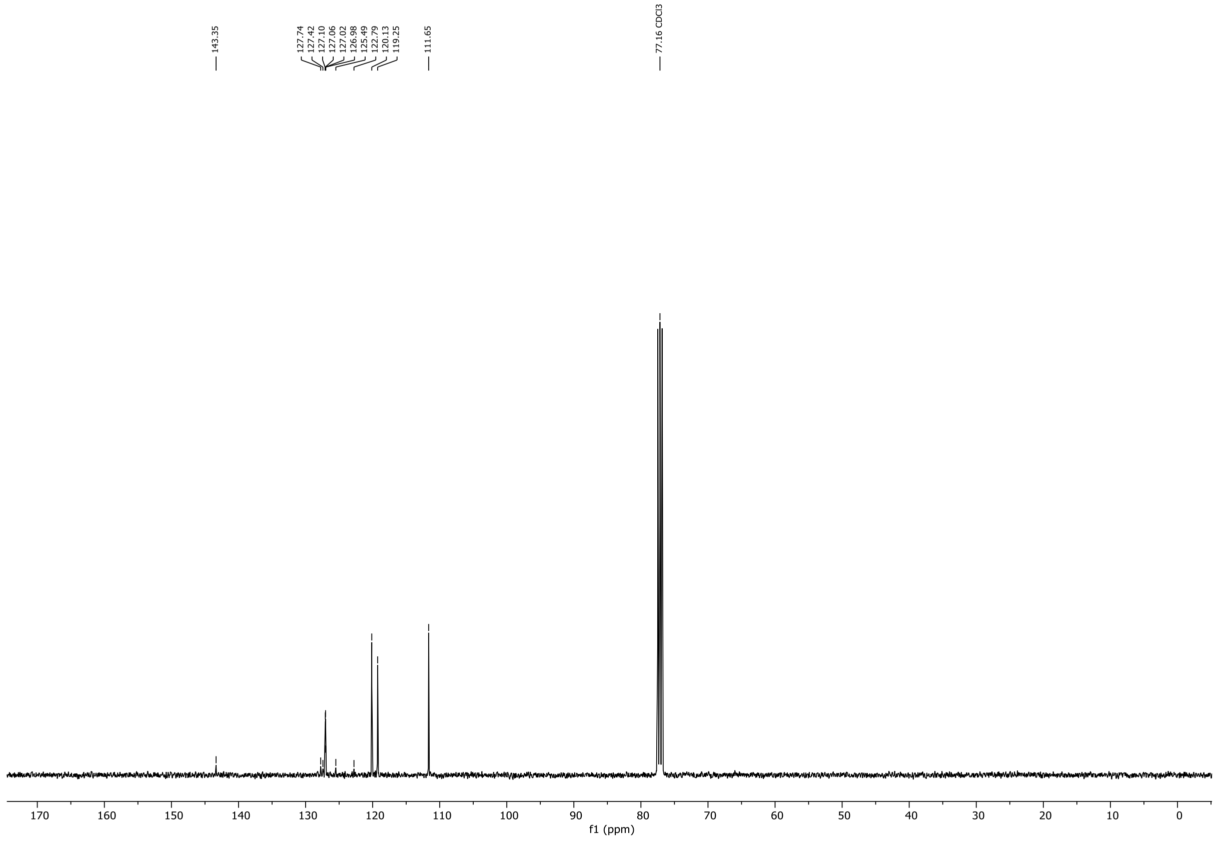

Figure S21. ^{13}C NMR spectrum of compound **10**.

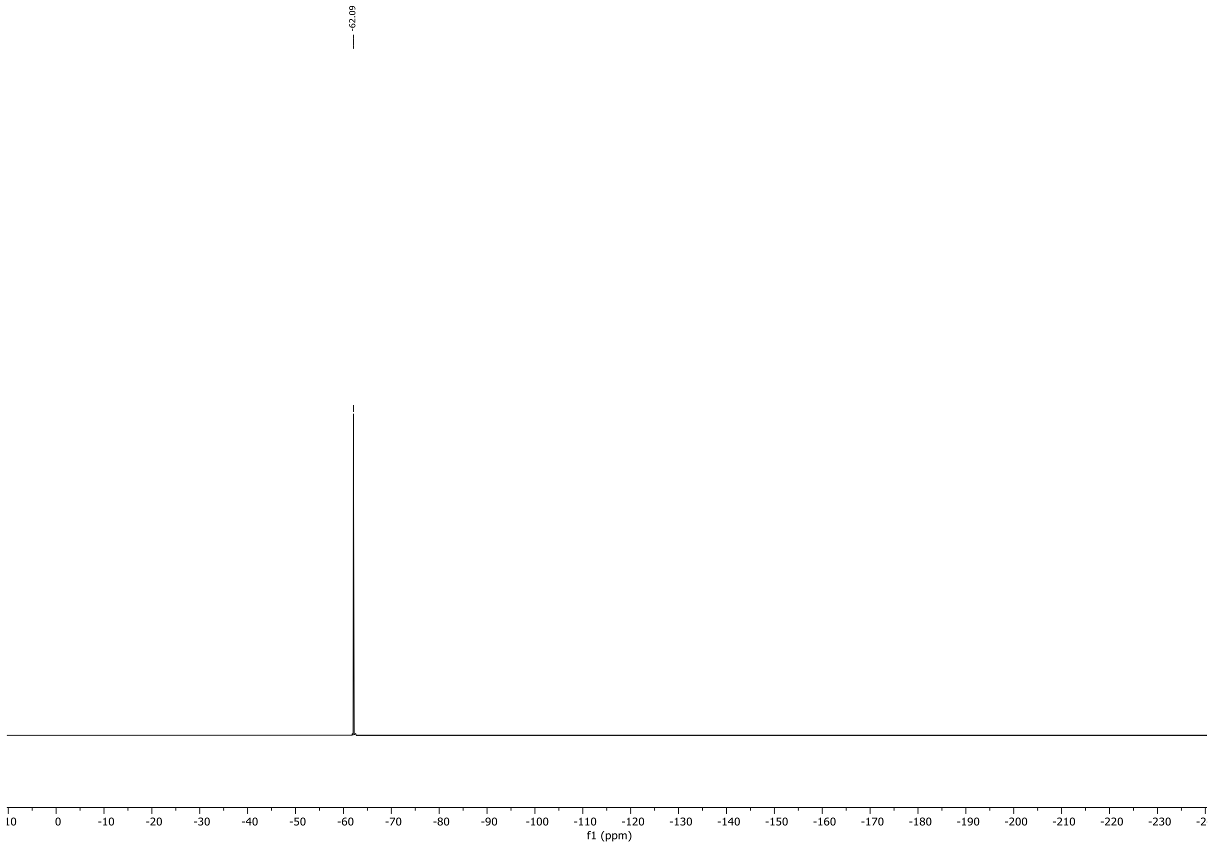

Figure S22. ^{19}F NMR spectrum of compound **10**.

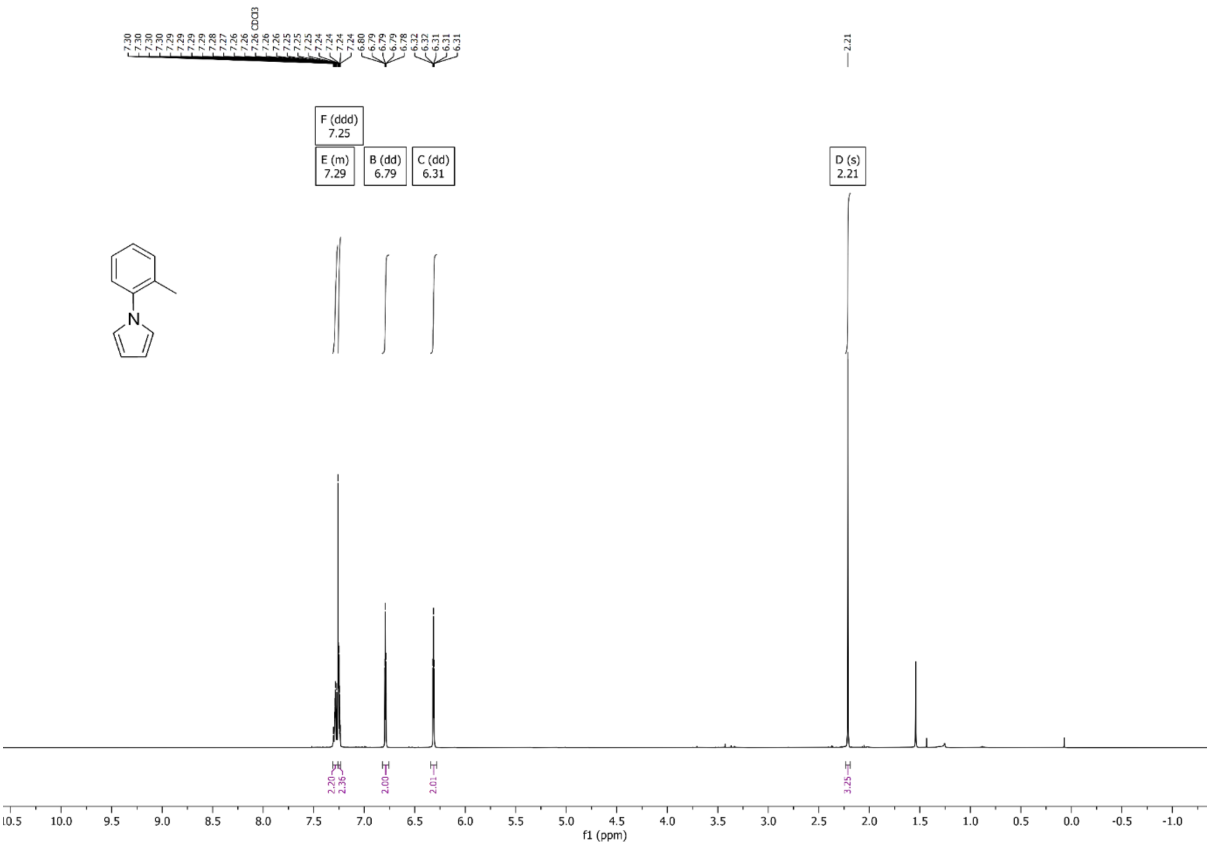

Figure S23. ^{1}H NMR spectrum of compound **11**.

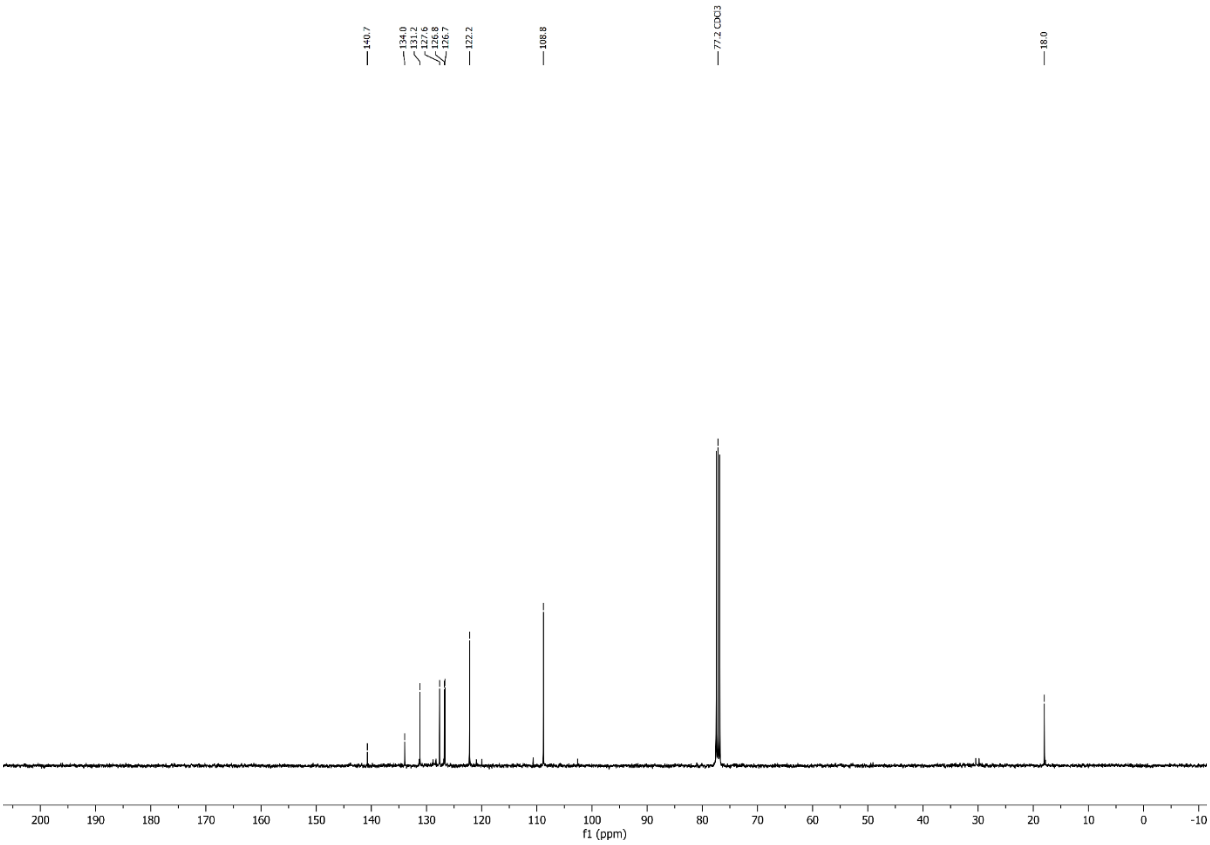

Figure S24. ^{13}C NMR spectrum of compound **11**.

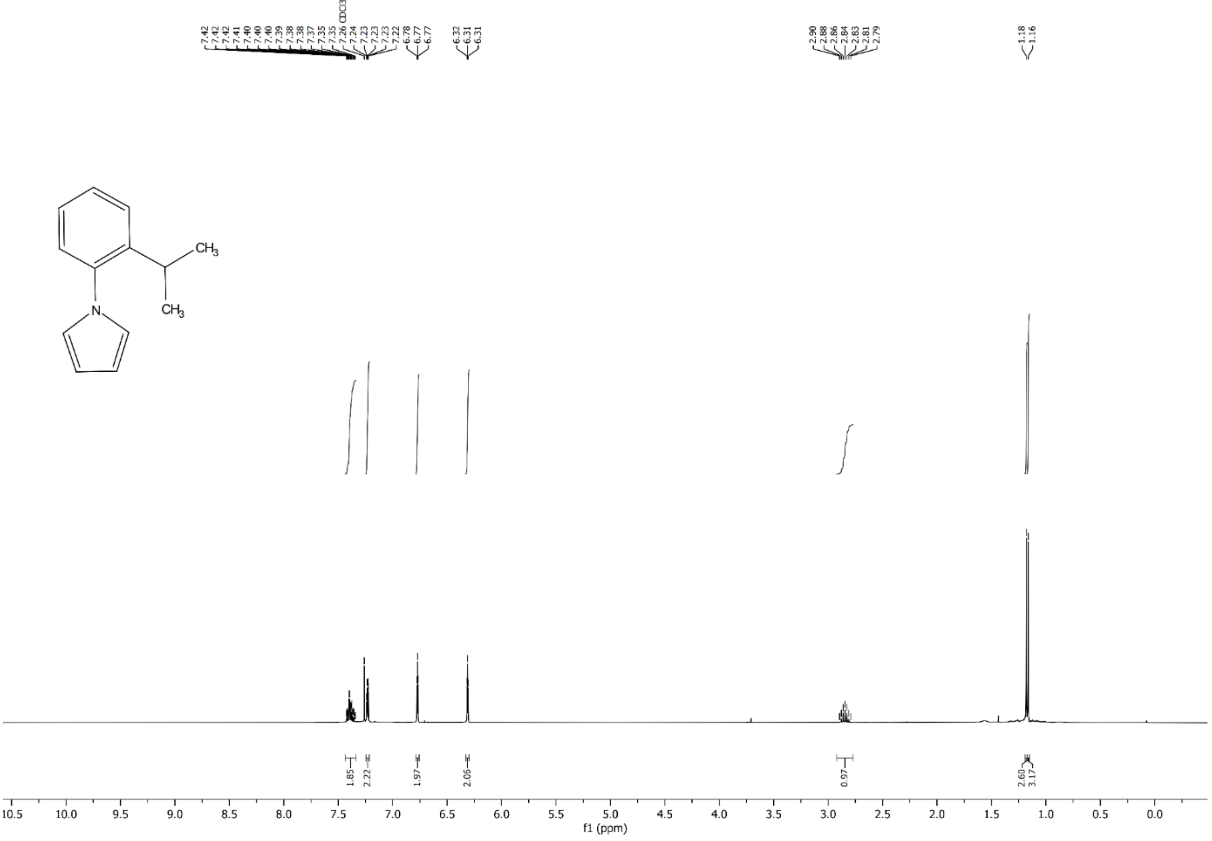

Figure S25. ^{1}H NMR spectrum of compound **12.**

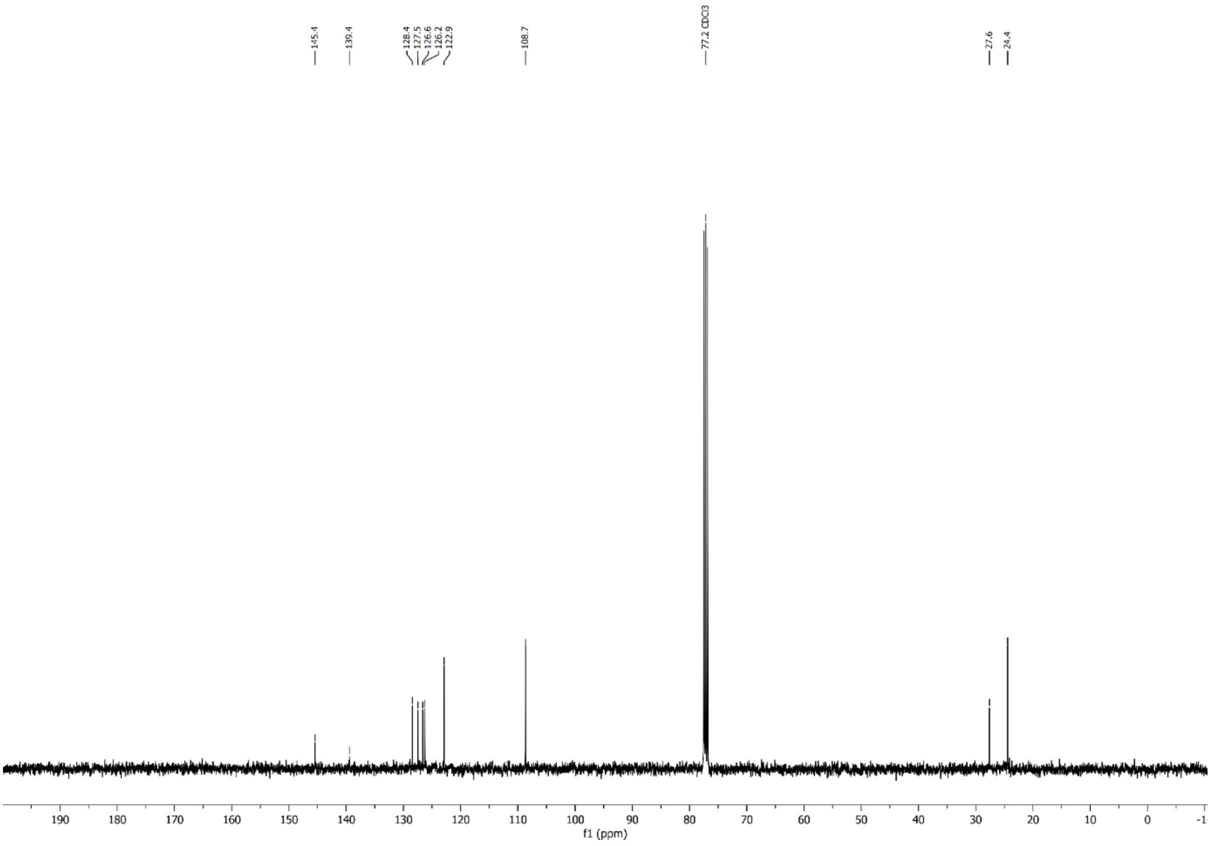

Figure S26. ^{13}C NMR spectrum of compound **12**.

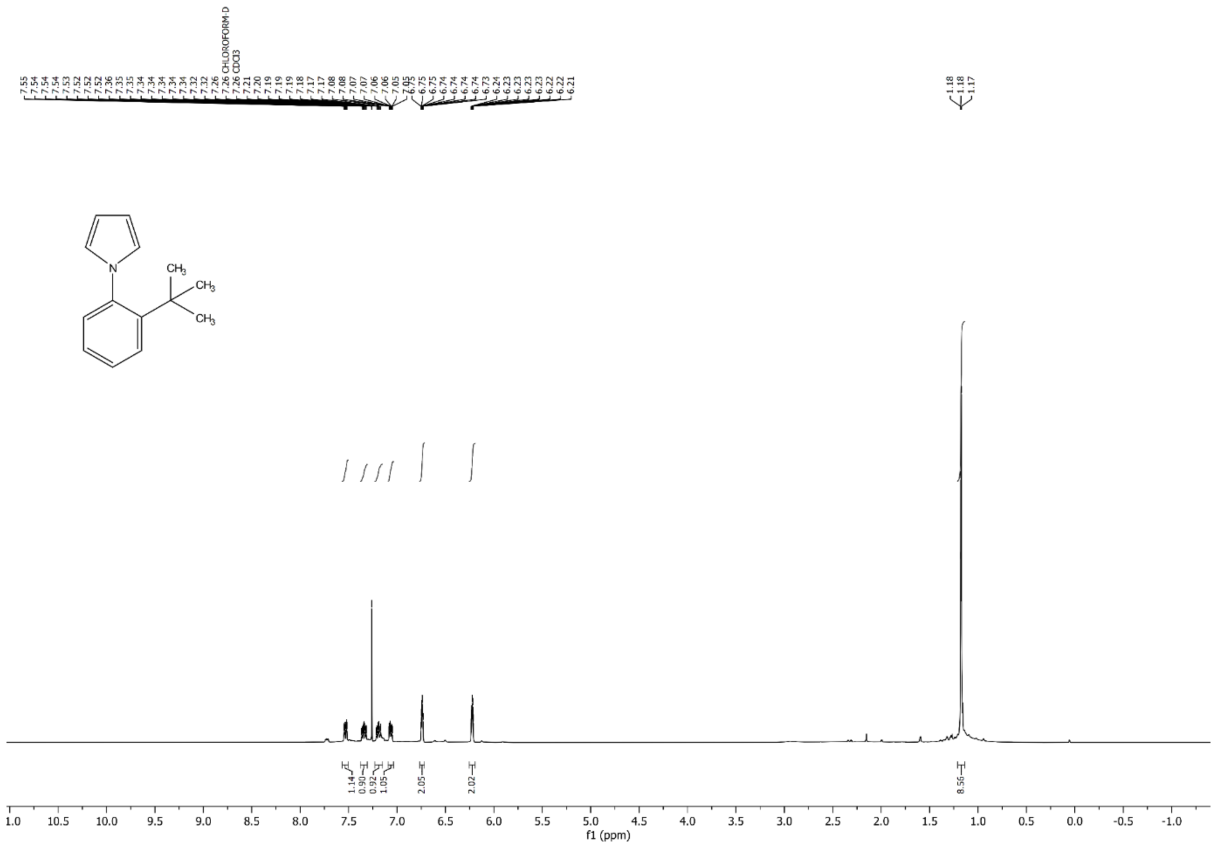

Figure S27. ^{1}H NMR spectrum of compound **13.**

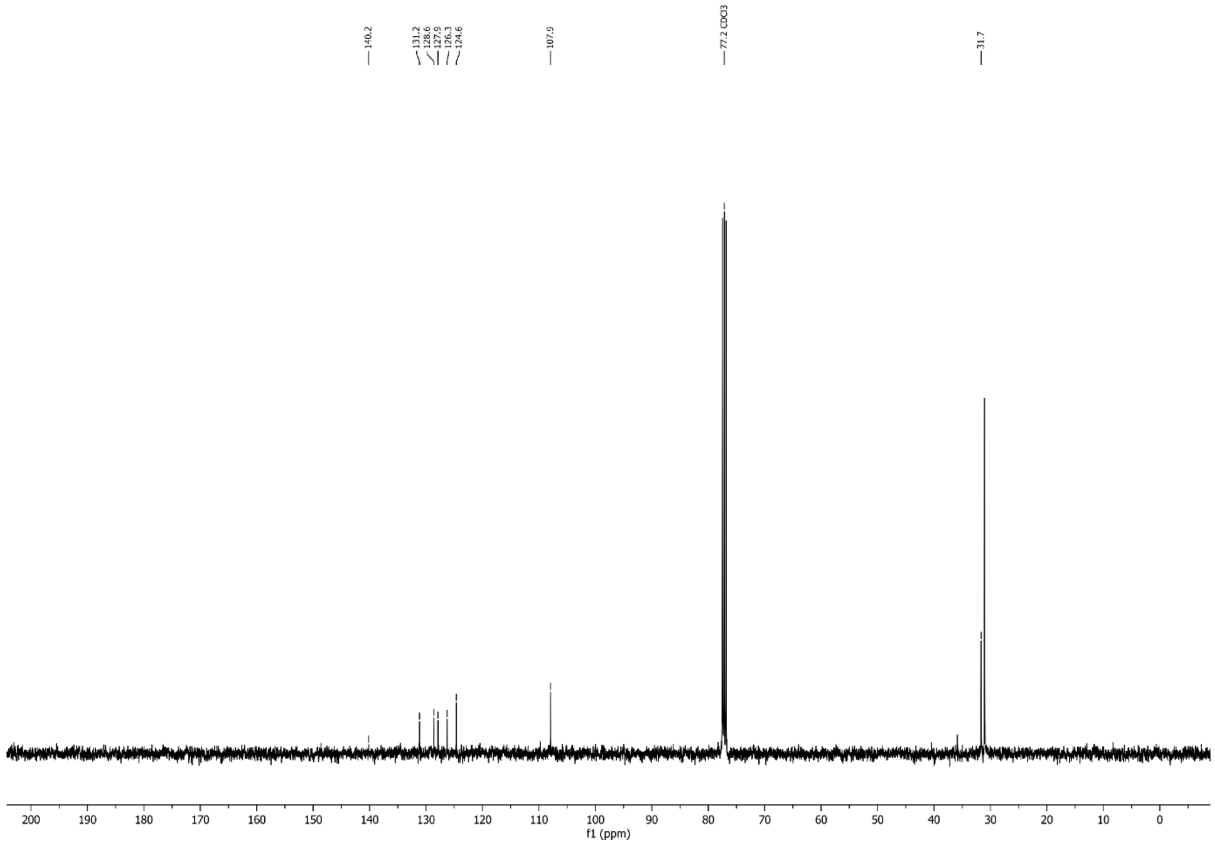

Figure S28. ^{13}C NMR spectrum of compound **13.**

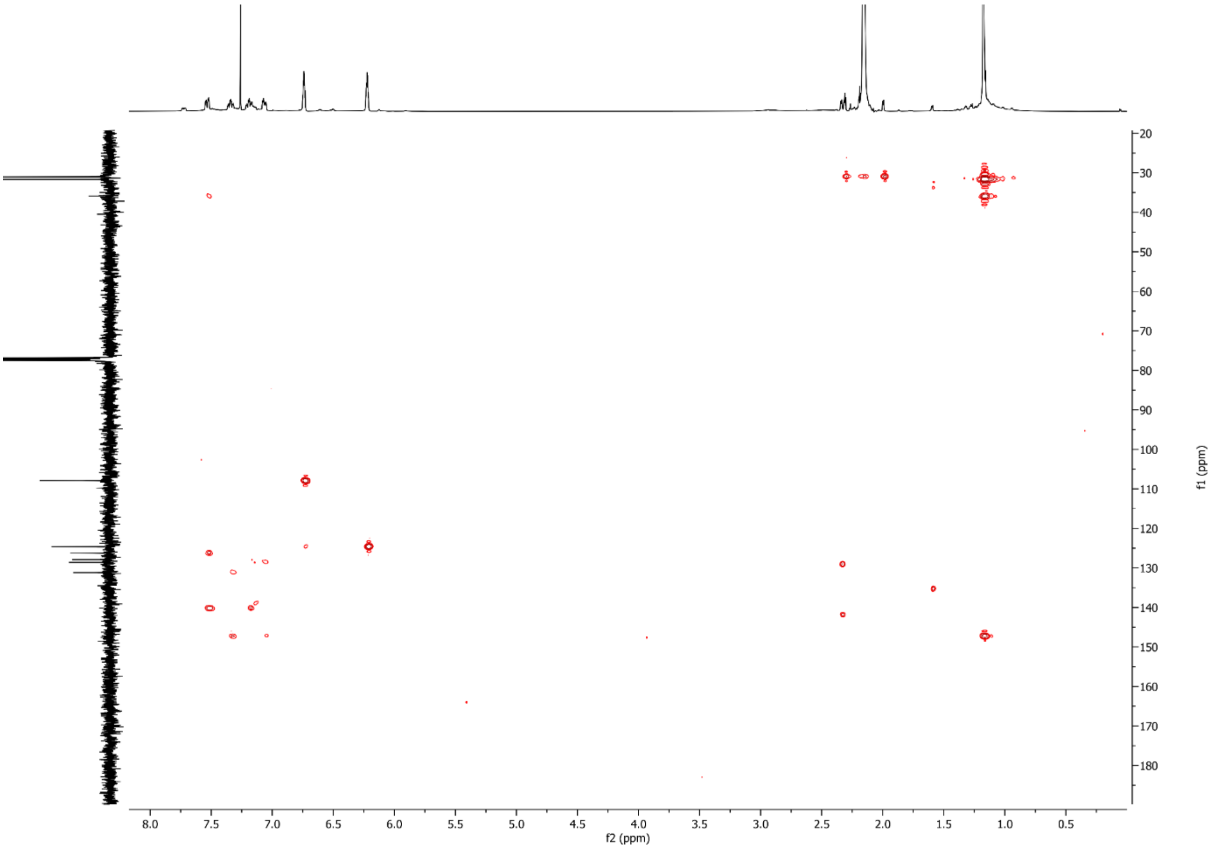

Figure S29. HMBC spectrum of compound **13.**

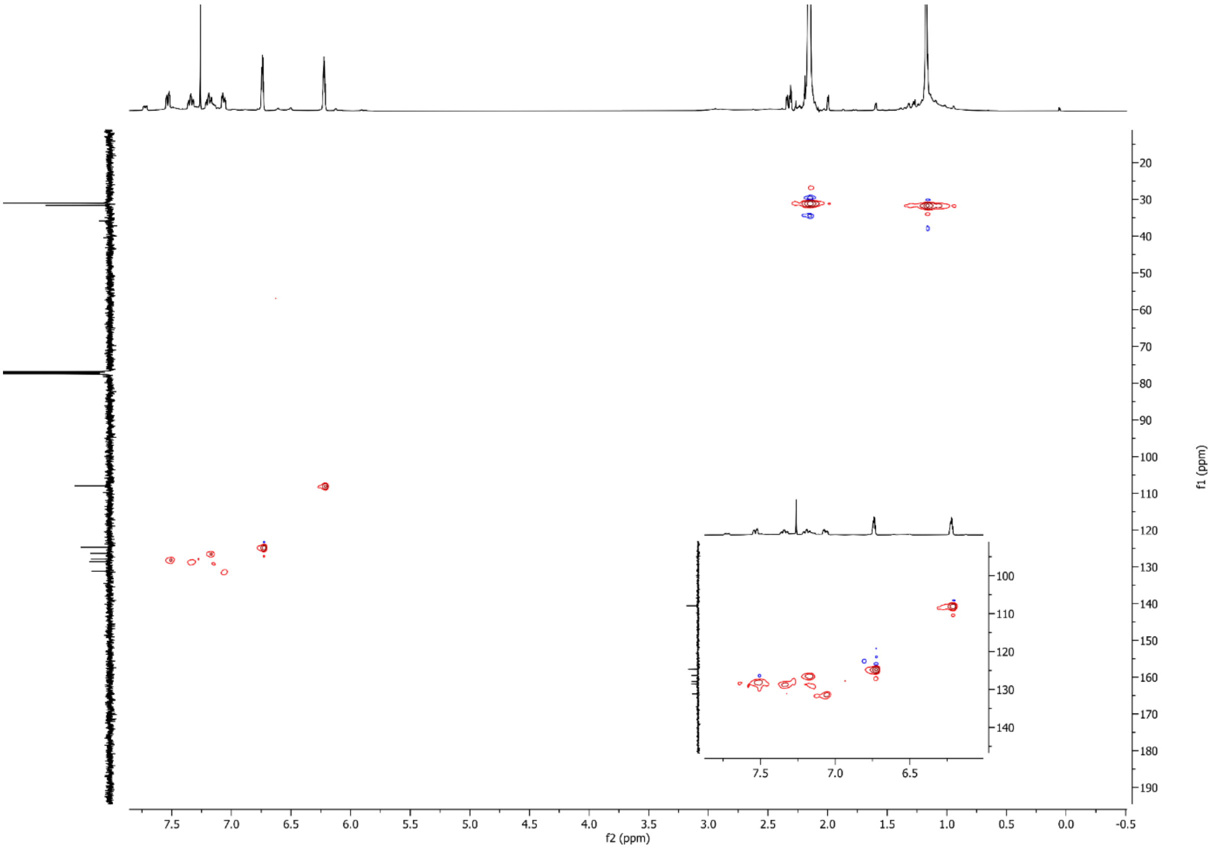

Figure S30. HSQC spectrum of compound **13.**

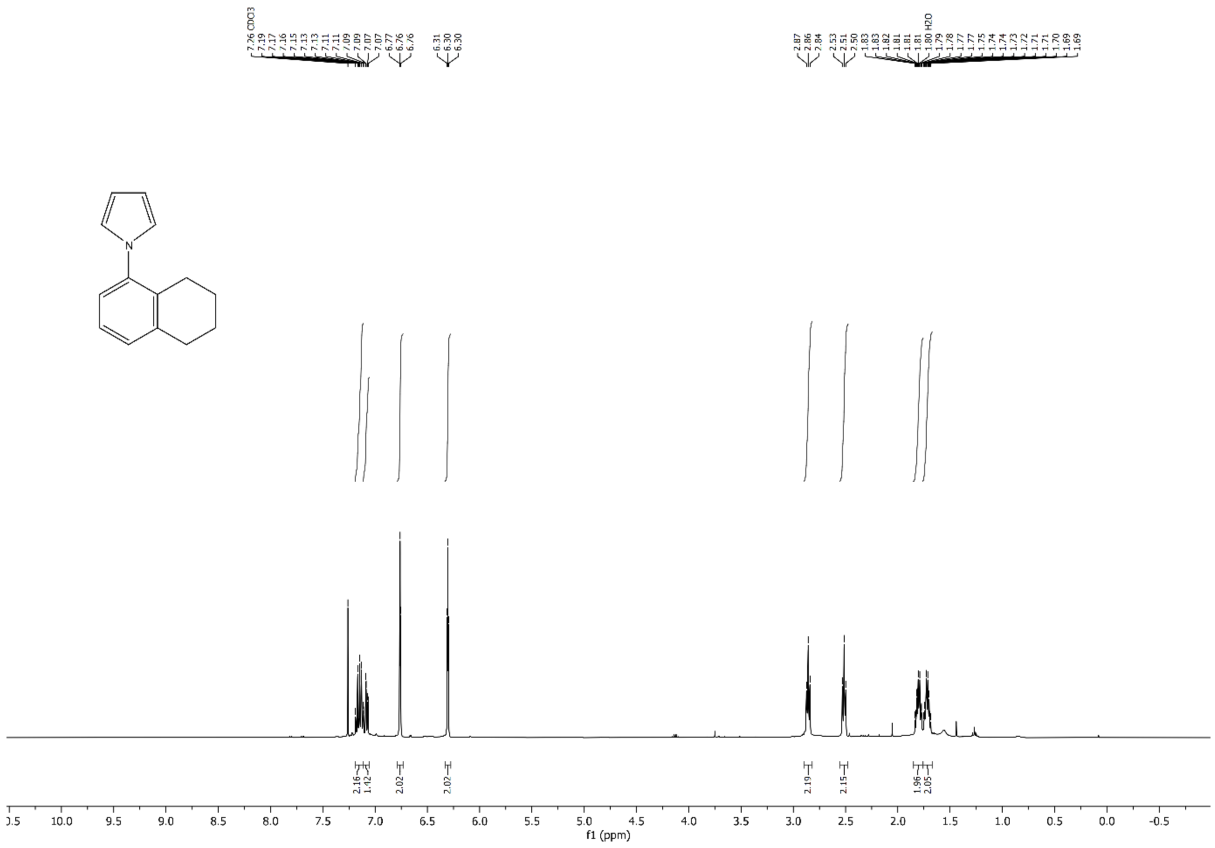

Figure S31. ^{1}H NMR spectrum of compound **14.**

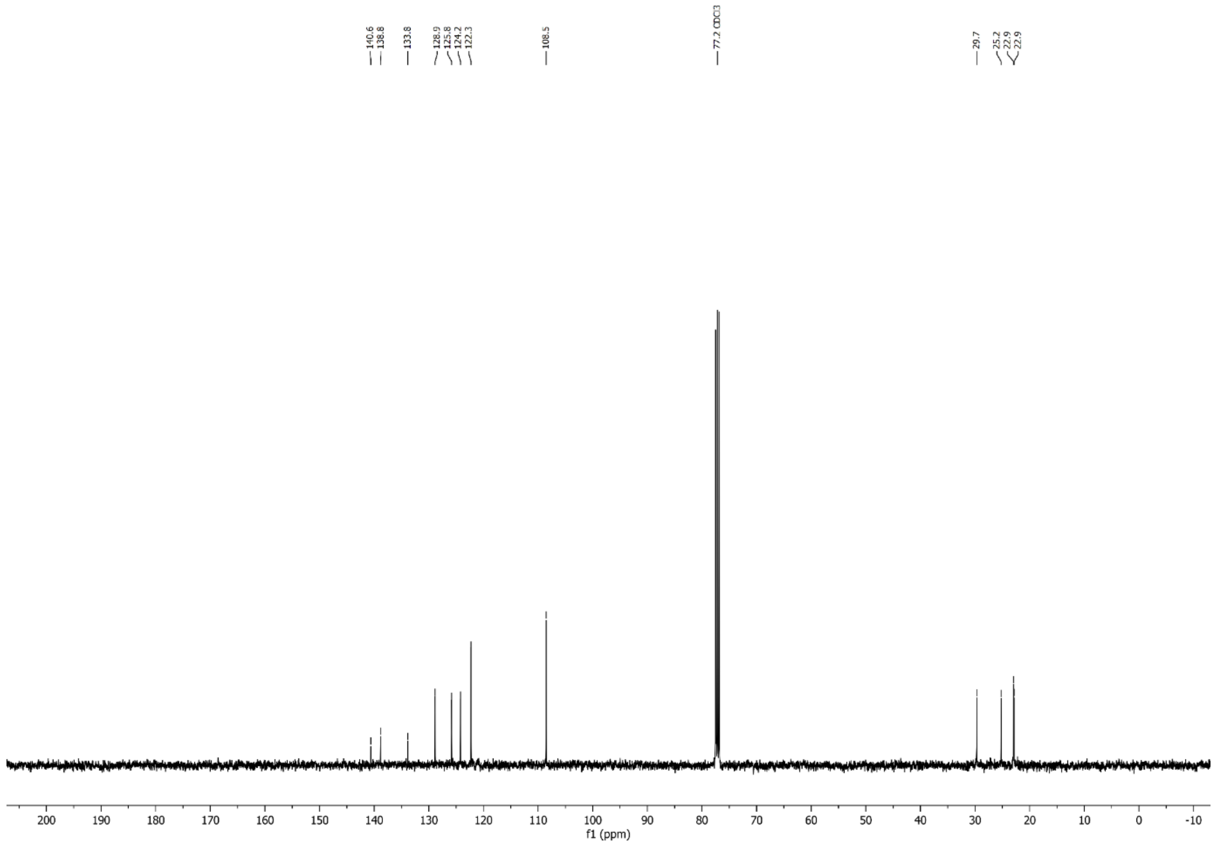

Figure S32. ^{13}C NMR spectrum of compound **14.**

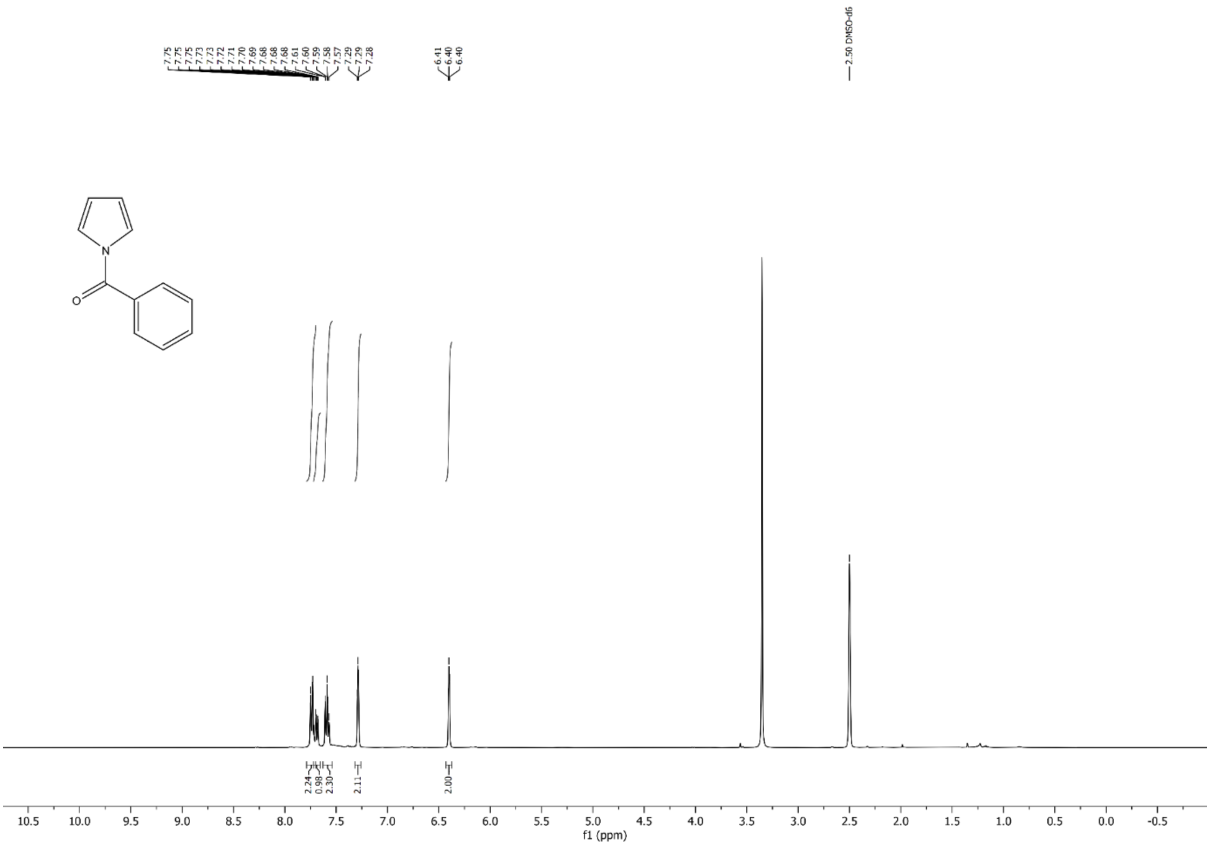

Figure S33. ^{1}H NMR spectrum of compound **15.**

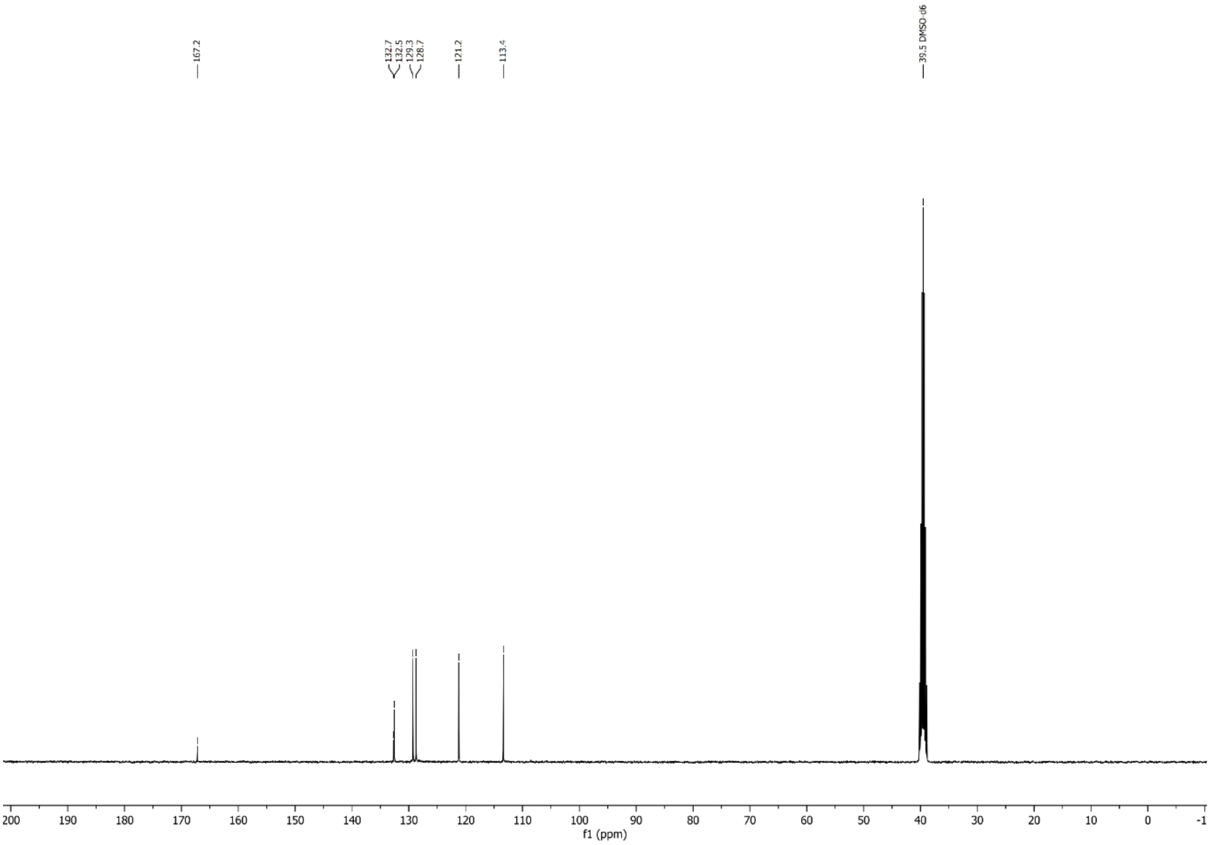

Figure S34. ^{13}C NMR spectrum of compound **15.**

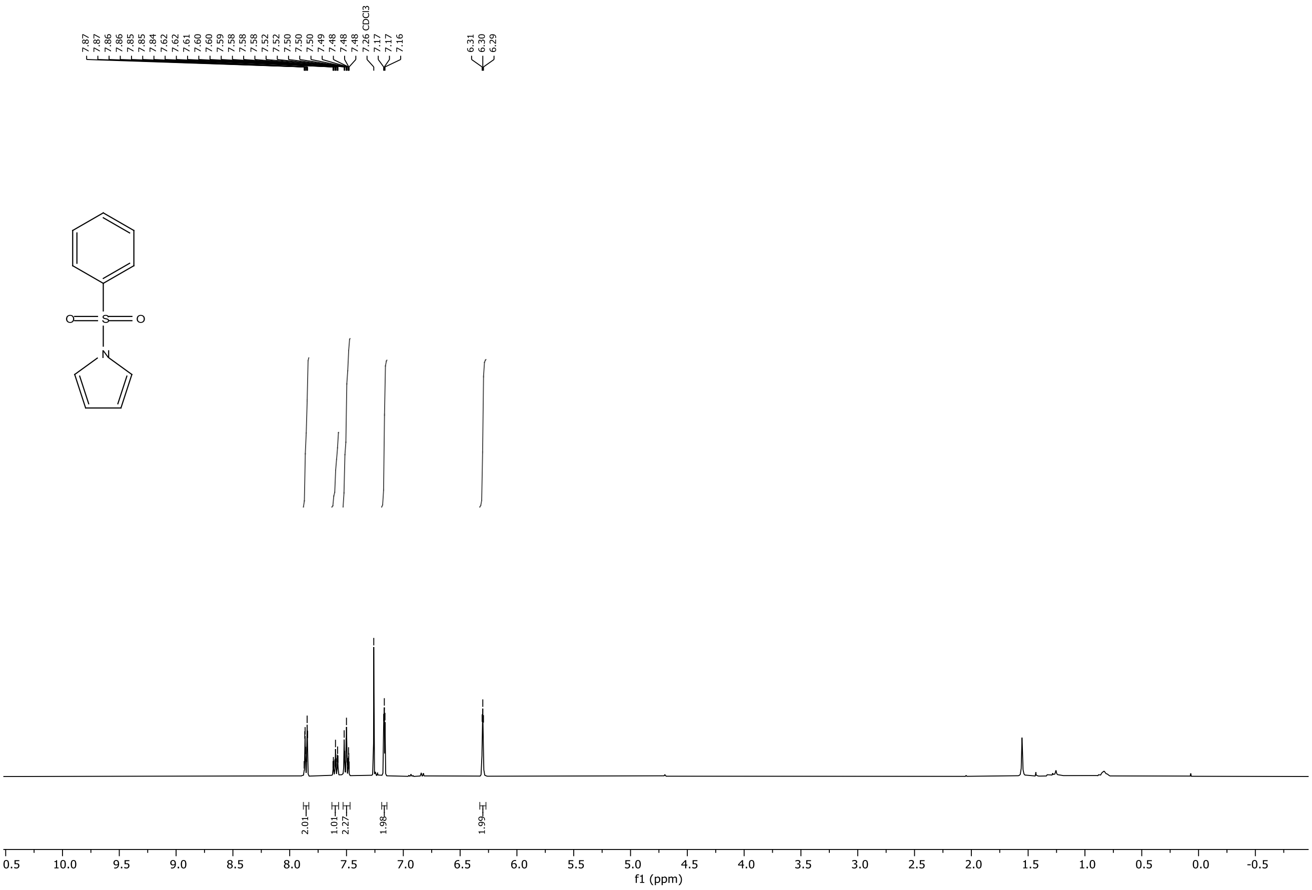

Figure S35. ^{1}H NMR spectrum of compound **16.**

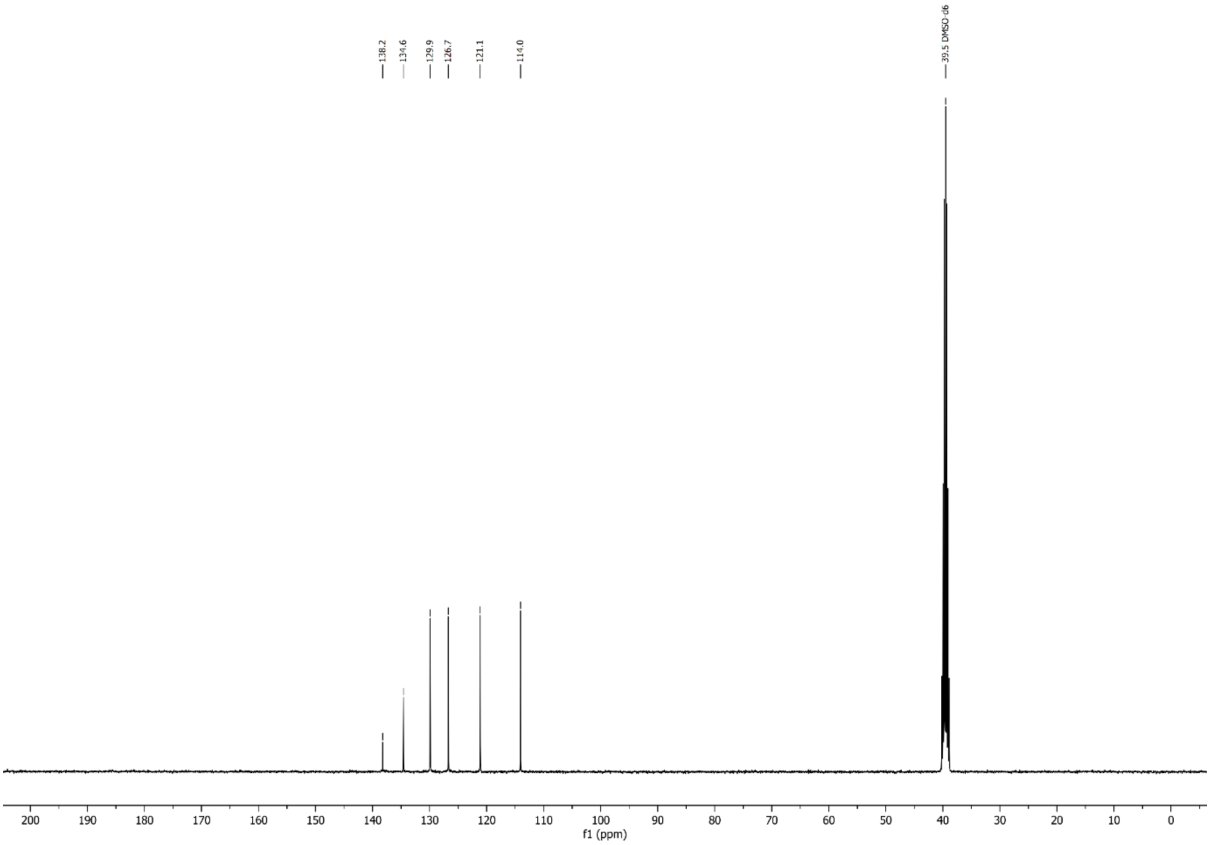

Figure S36. ^{13}C NMR spectrum of compound **16.**

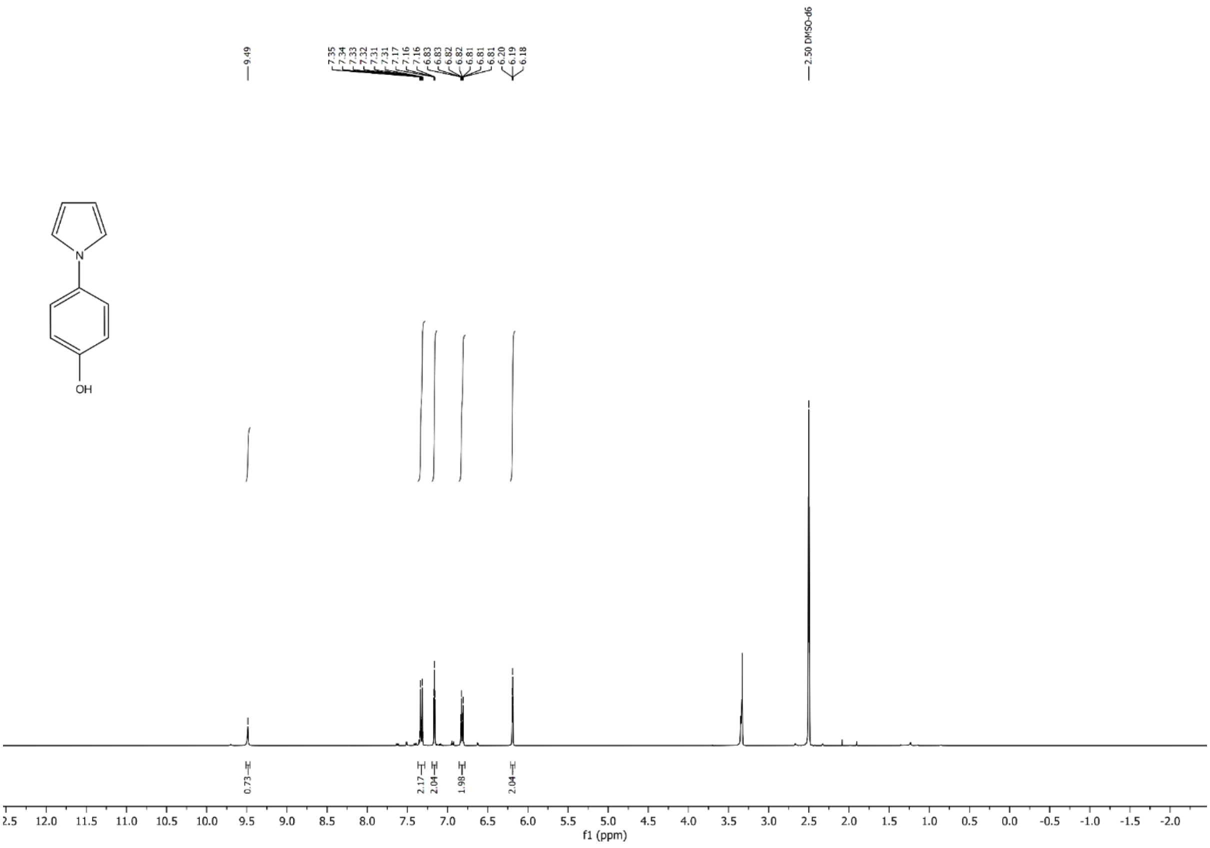

Figure S37. ^{1}H NMR spectrum of compound **17.**

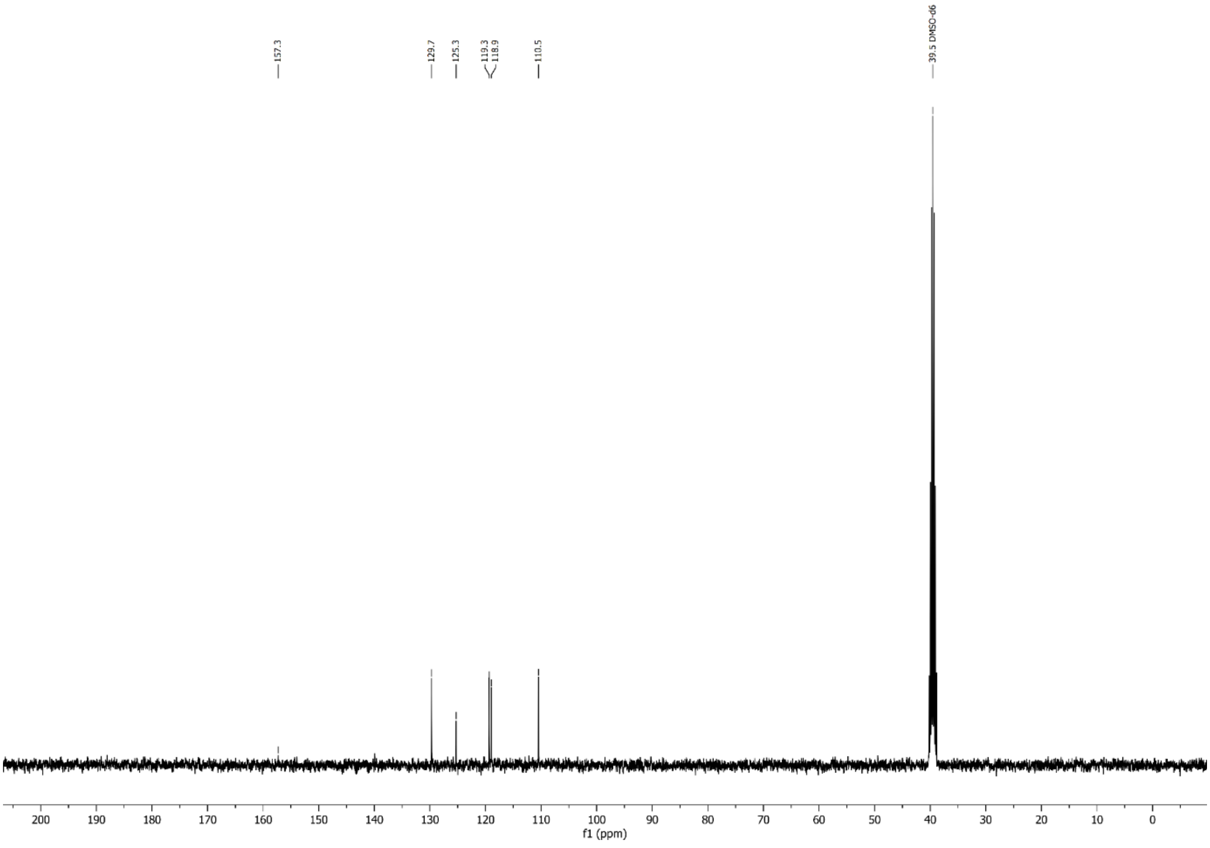

Figure S38. ^{13}C NMR spectrum of compound **17.**

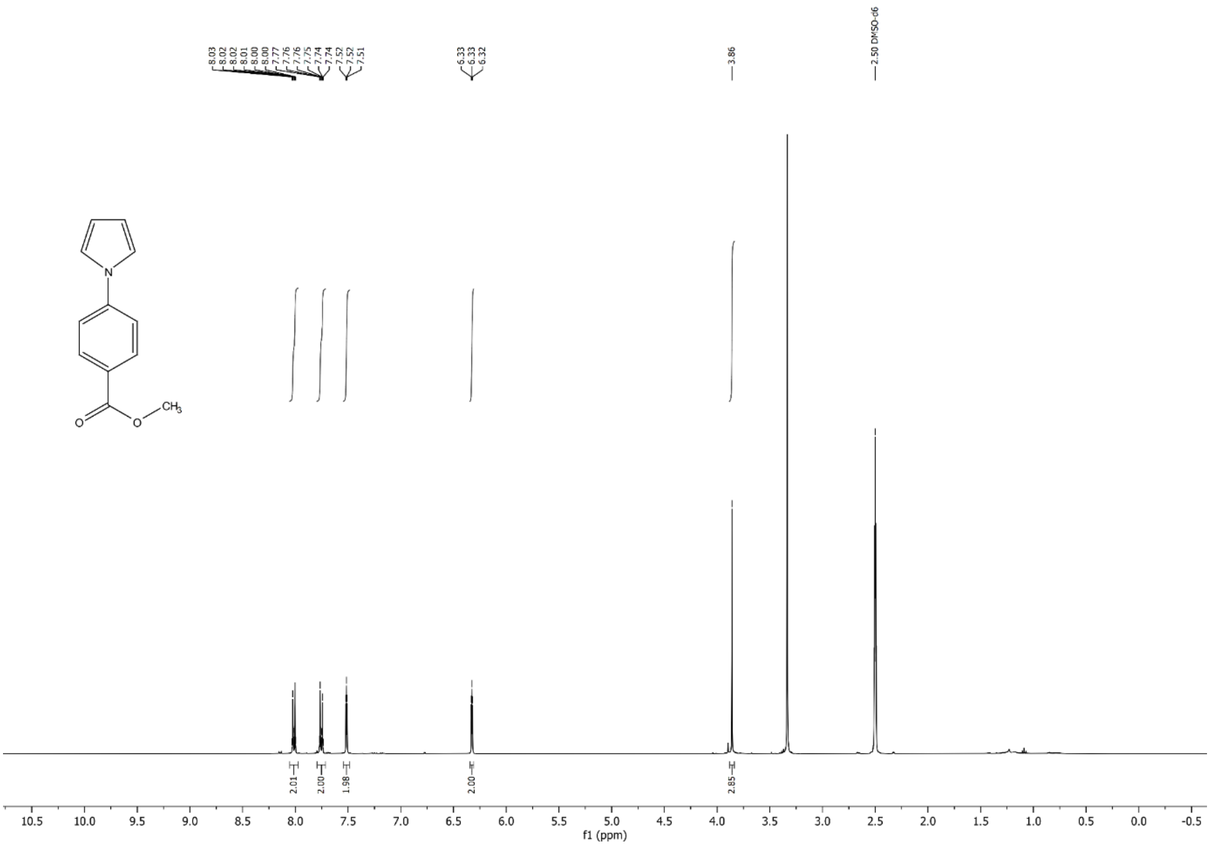

Figure S39. ^{1}H NMR spectrum of compound **18.**

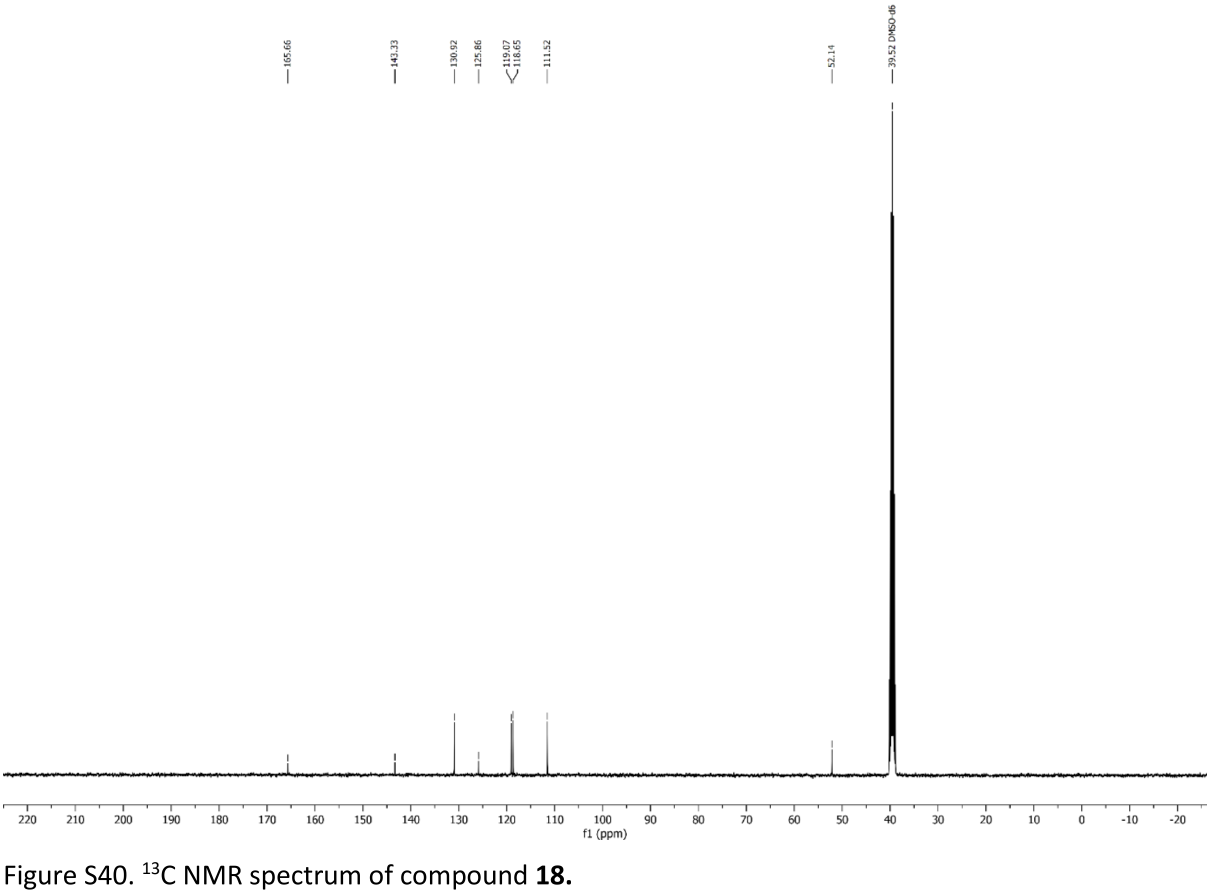

Figure S40. ^{13}C NMR spectrum of compound **18.**

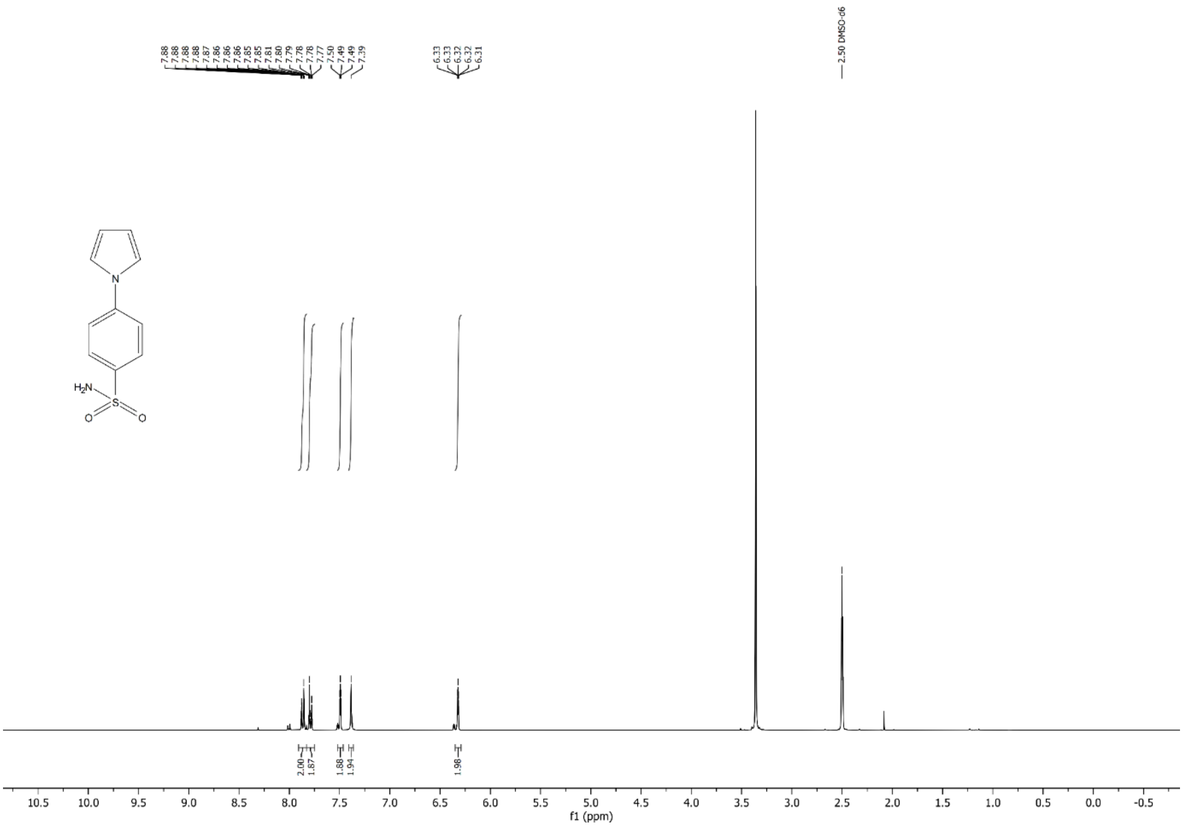

Figure S41. ^{1}H NMR spectrum of compound **19.**

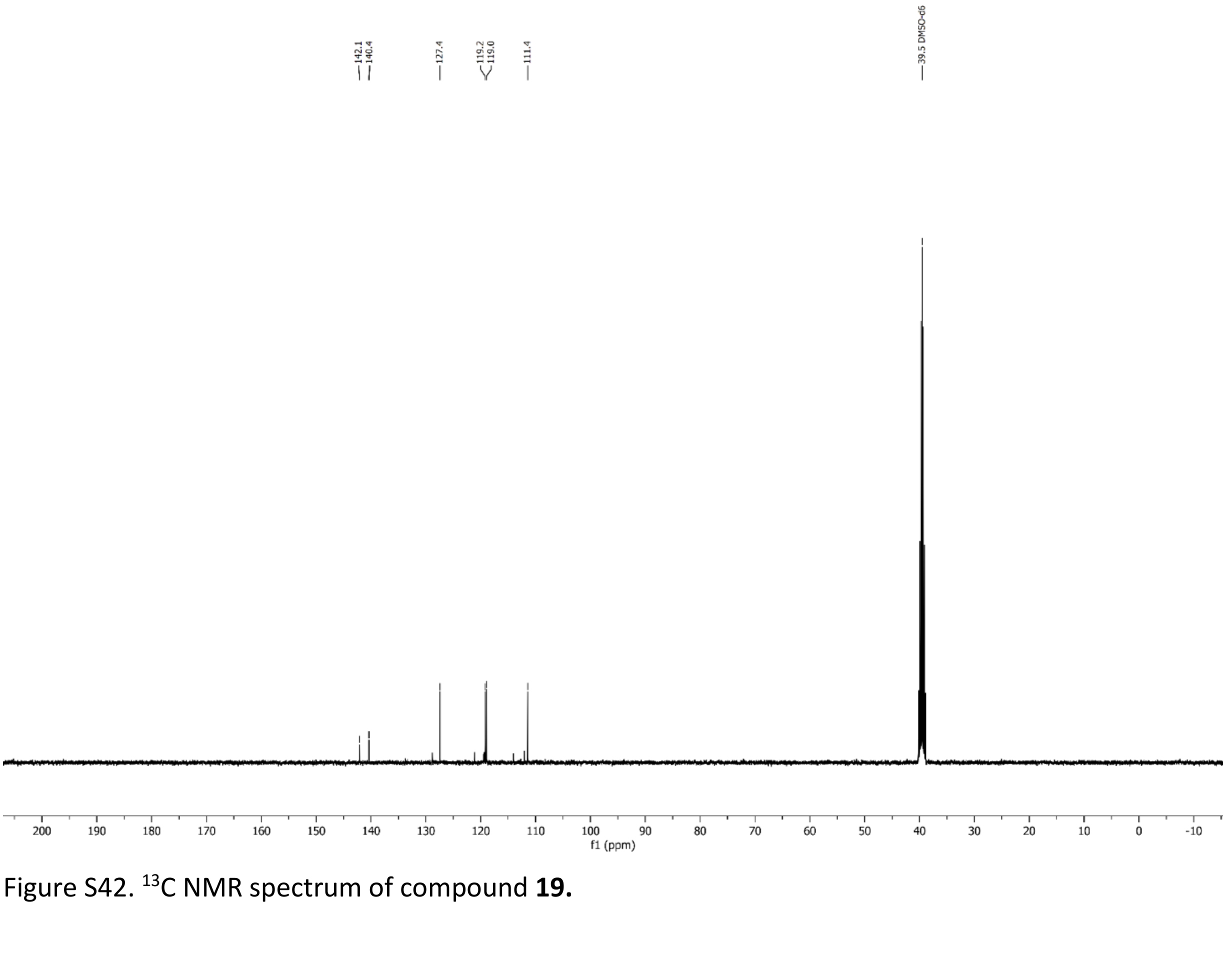

Figure S42. ^{13}C NMR spectrum of compound **19.**

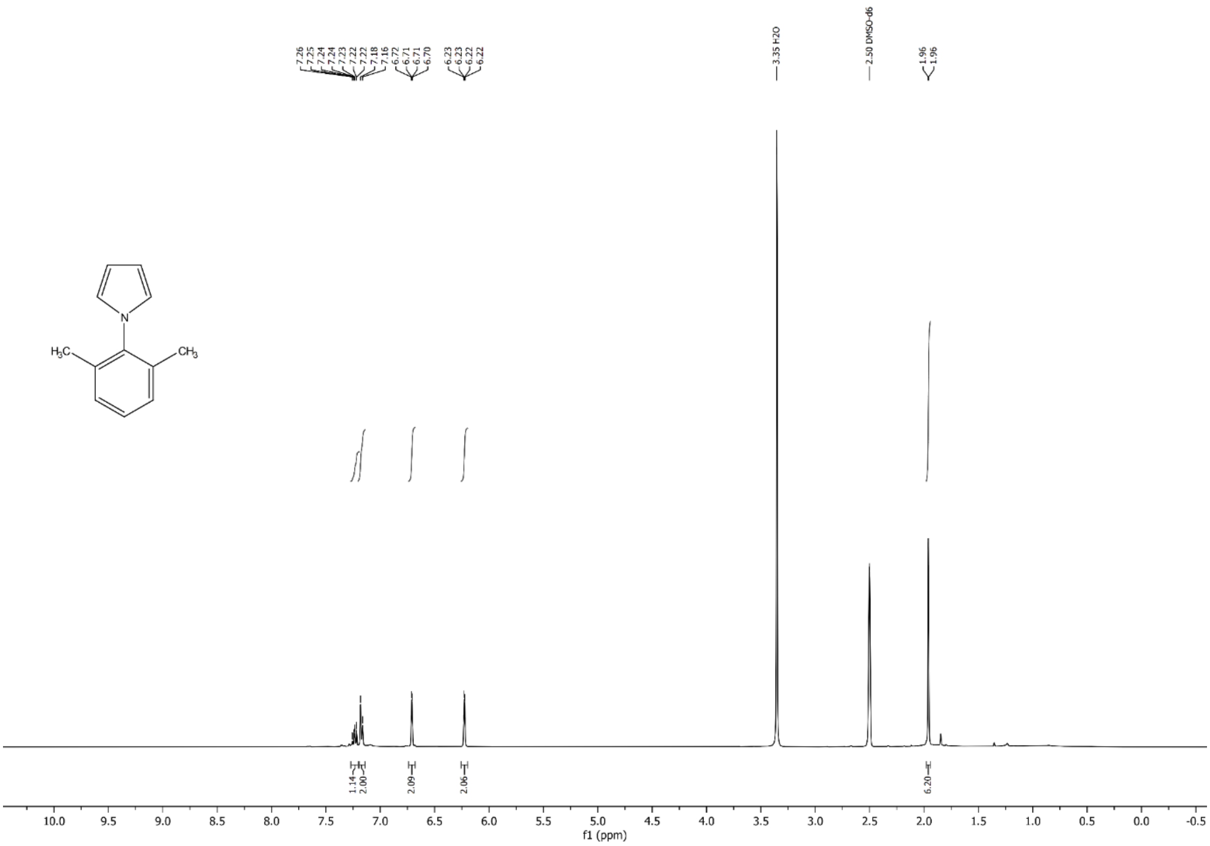

Figure S43. [1] H NMR spectrum of compound **20.**

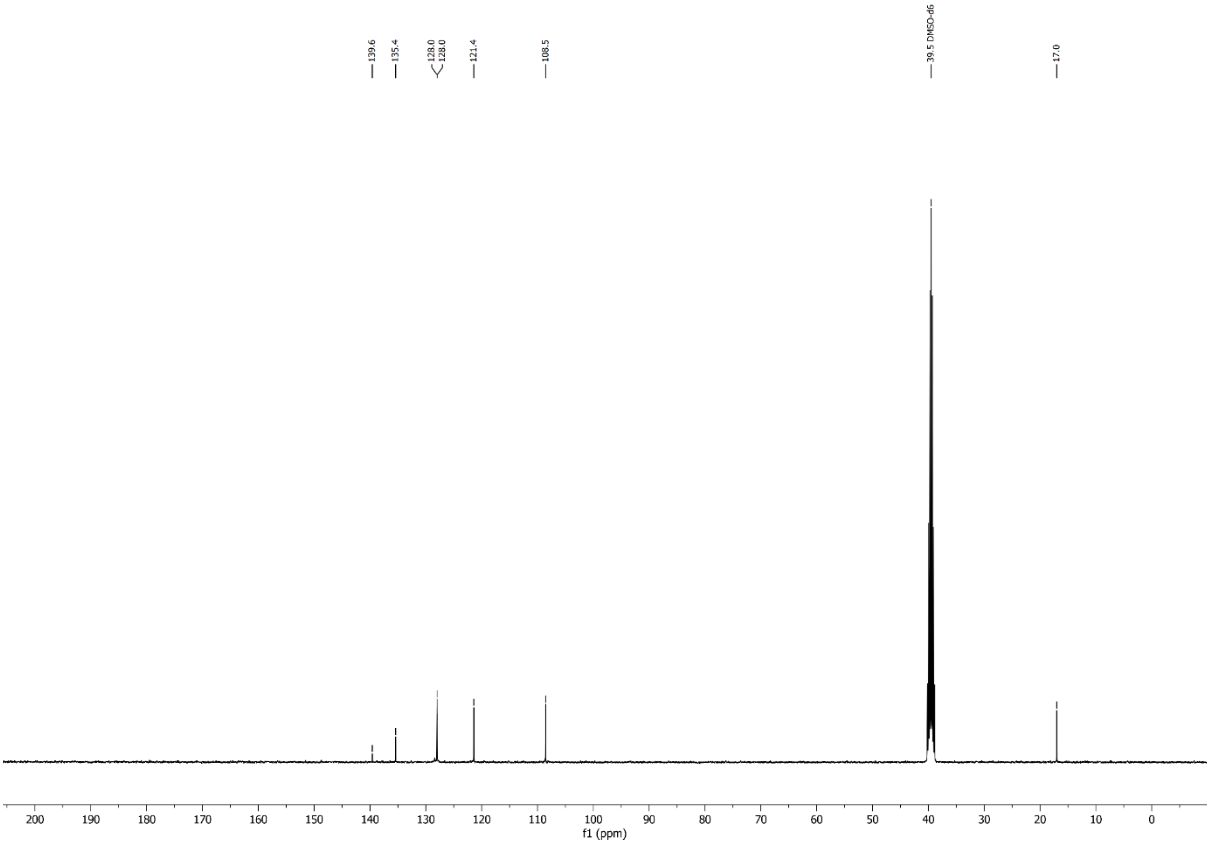

Figure S44. ^{13}C NMR spectrum of compound **20.**

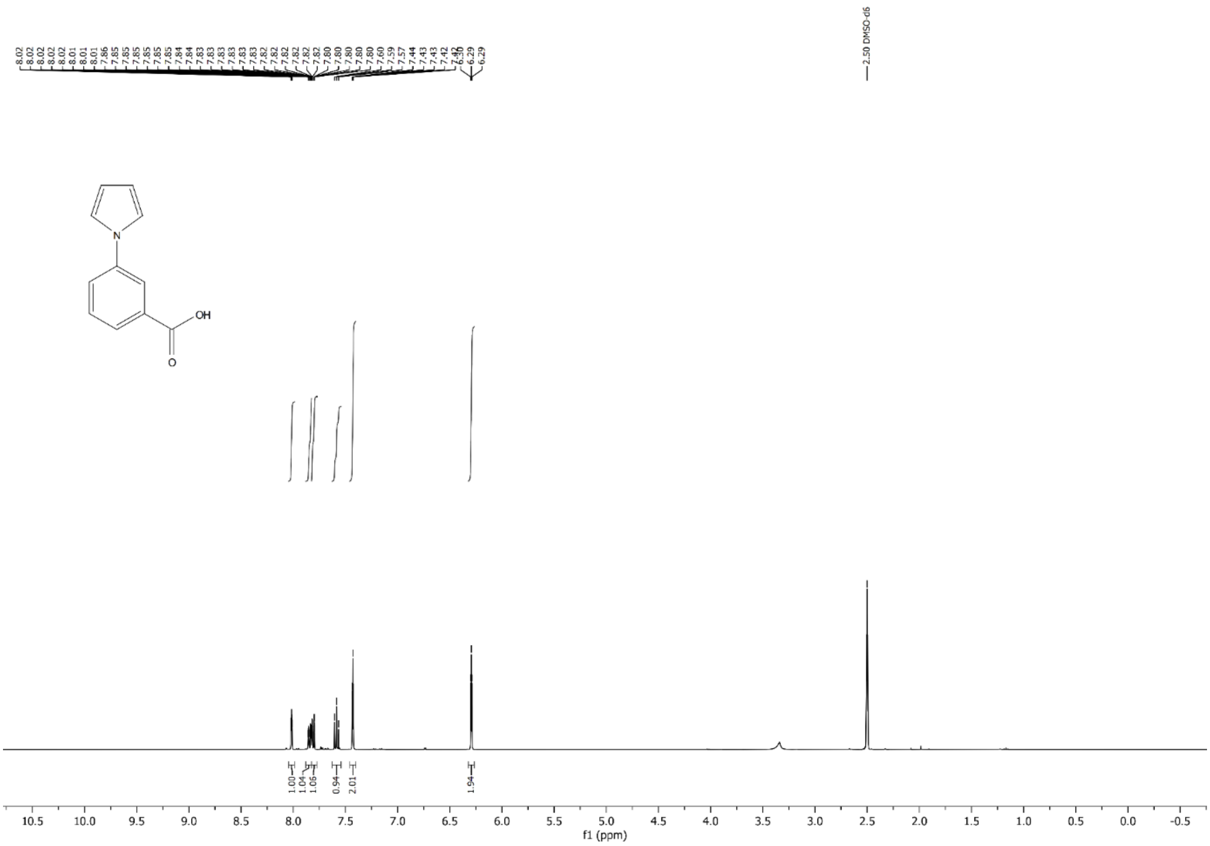

Figure S45. ^{1}H NMR spectrum of compound **21.**

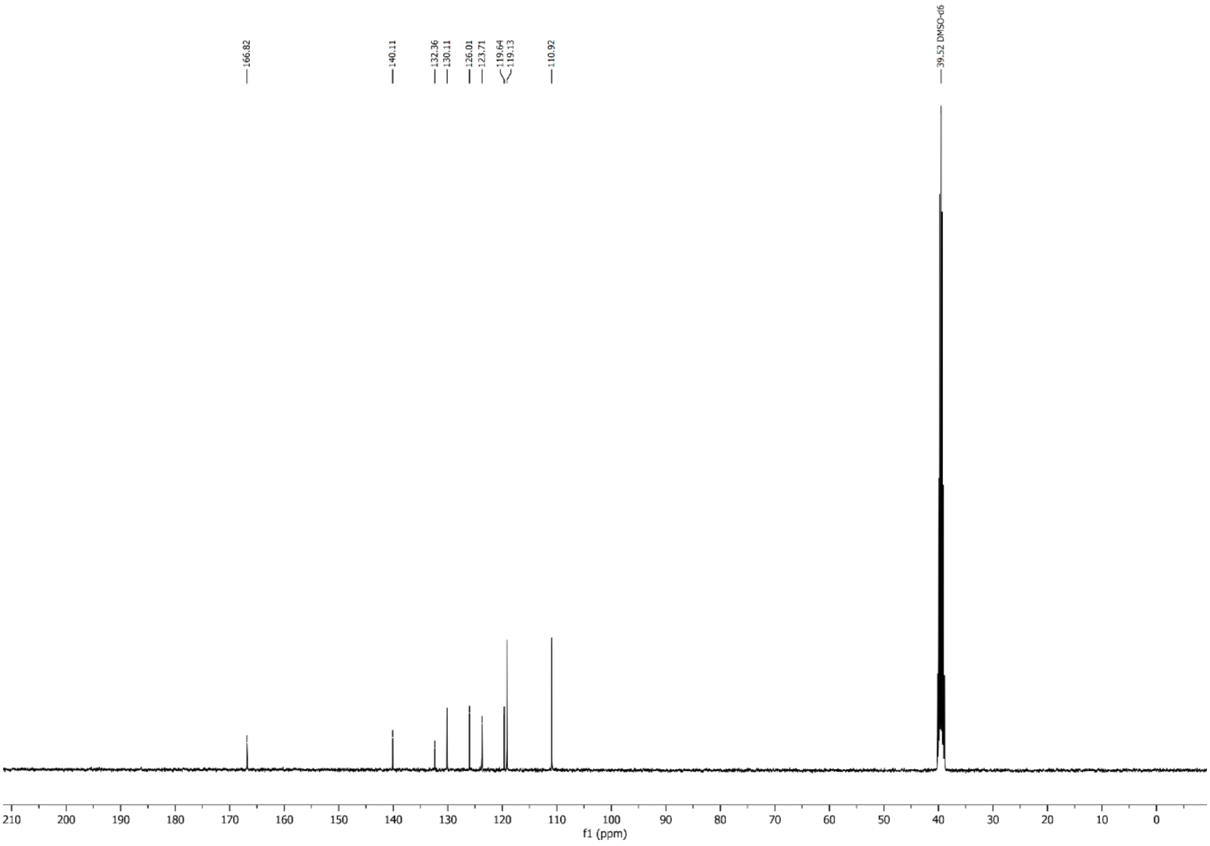

Figure S46. ^{13}C NMR spectrum of compound **21.**

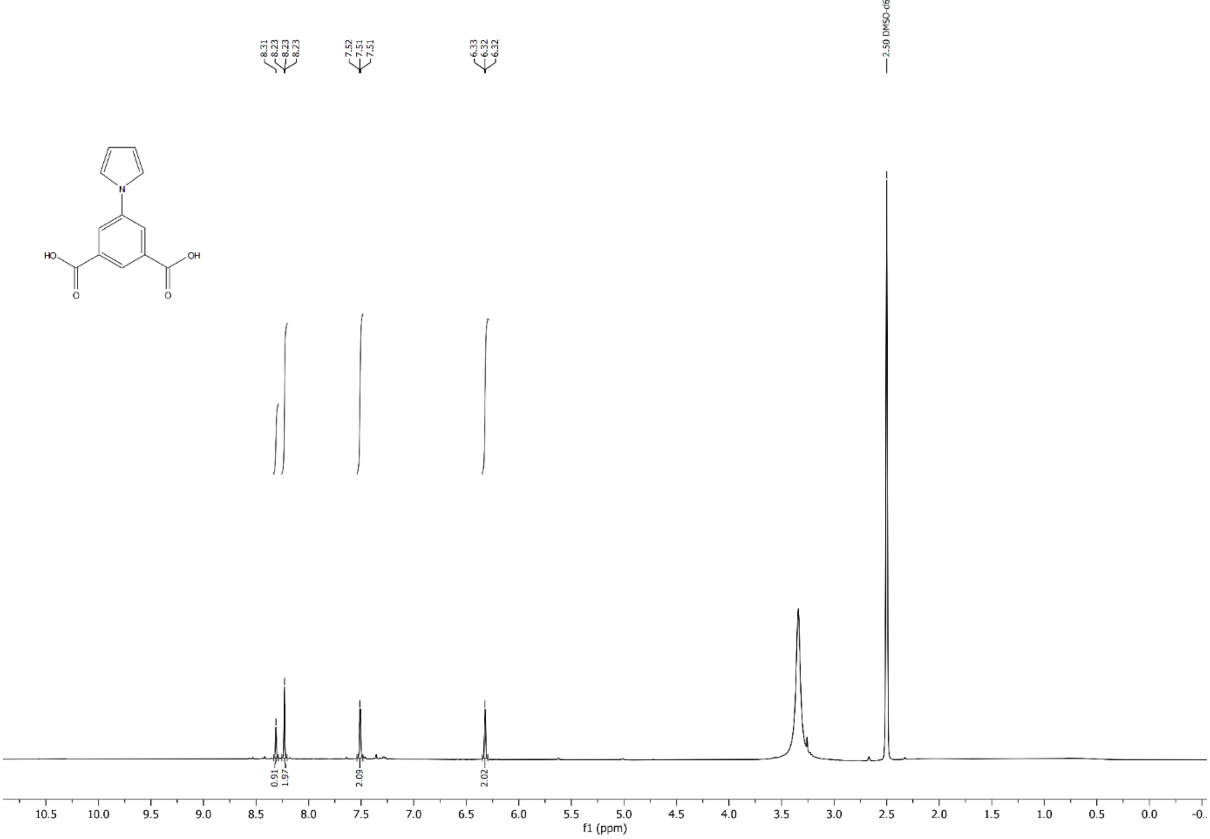

Figure S47. ^{1}H NMR spectrum of compound **22.**

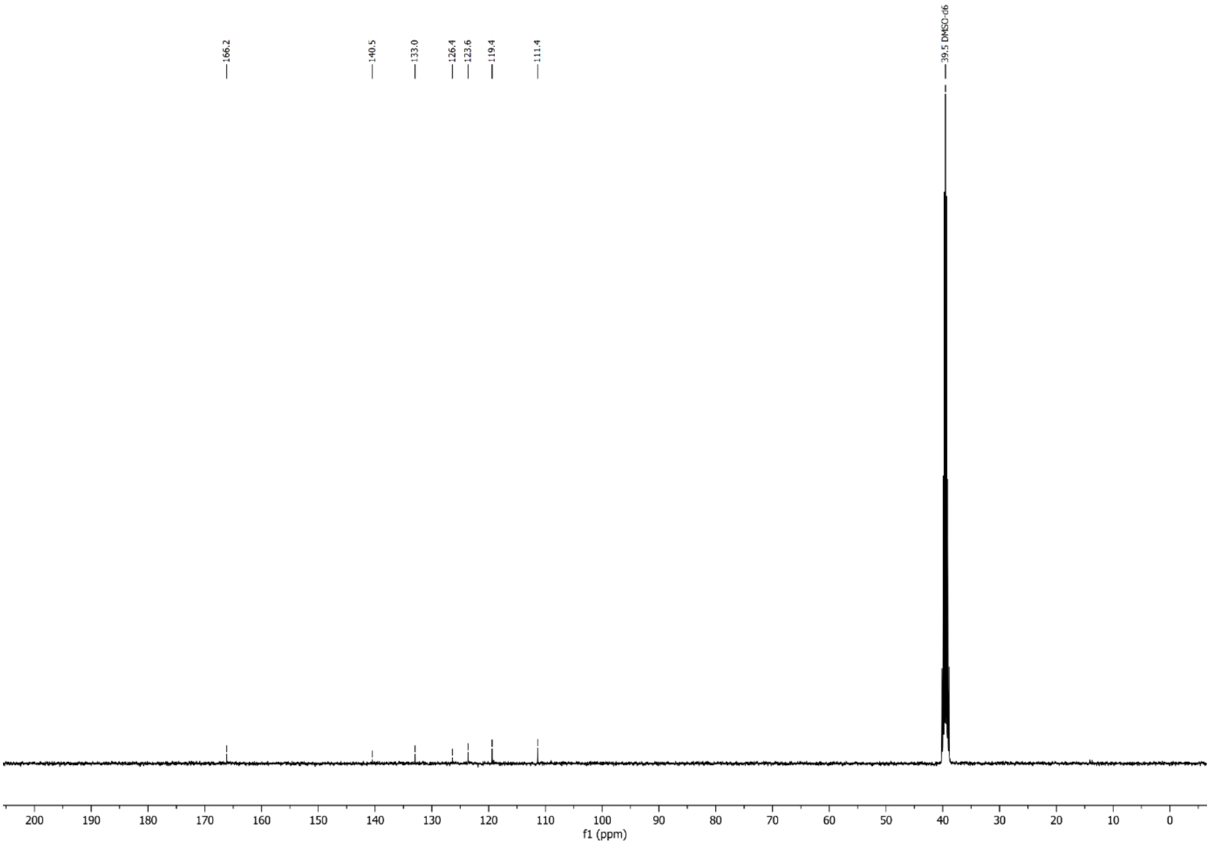

Figure S48. ^{13}C NMR spectrum of compound **22.**

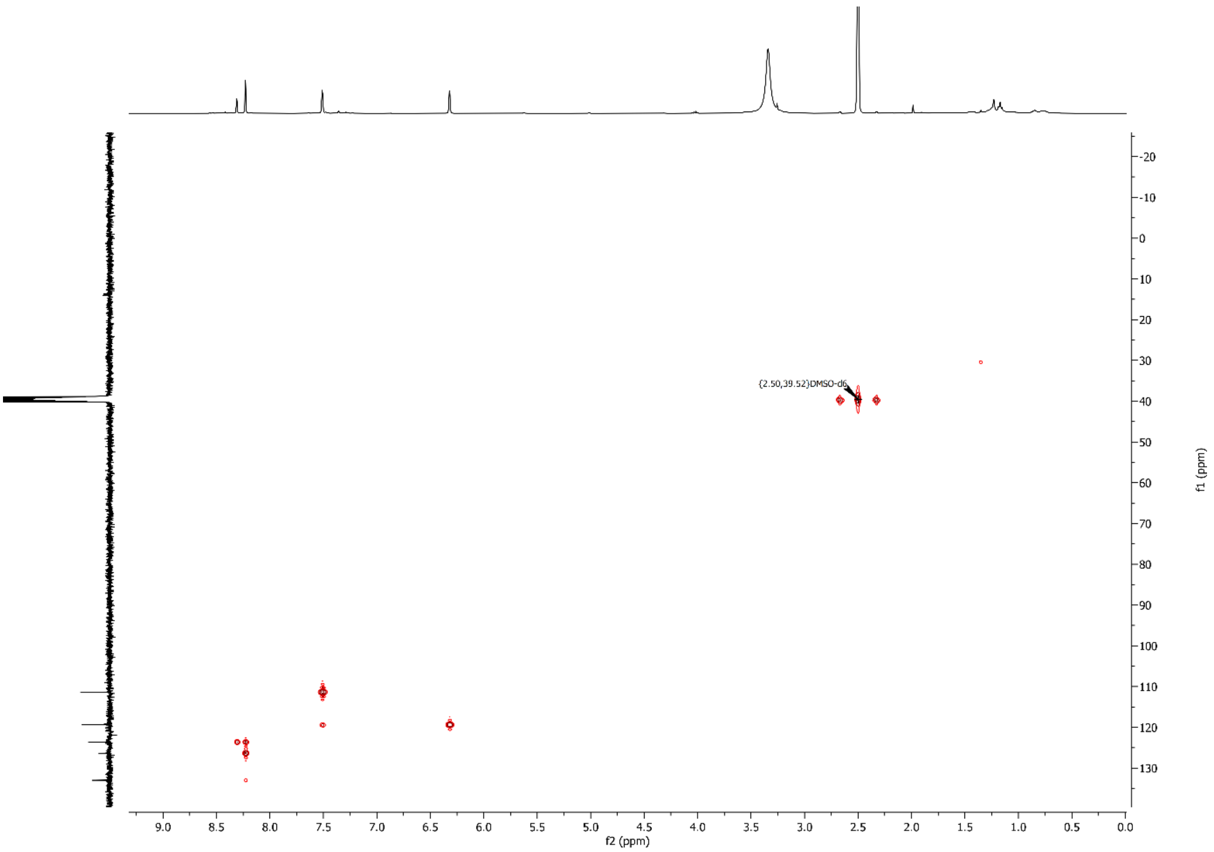

Figure S49. HMBC spectrum of compound **22.**

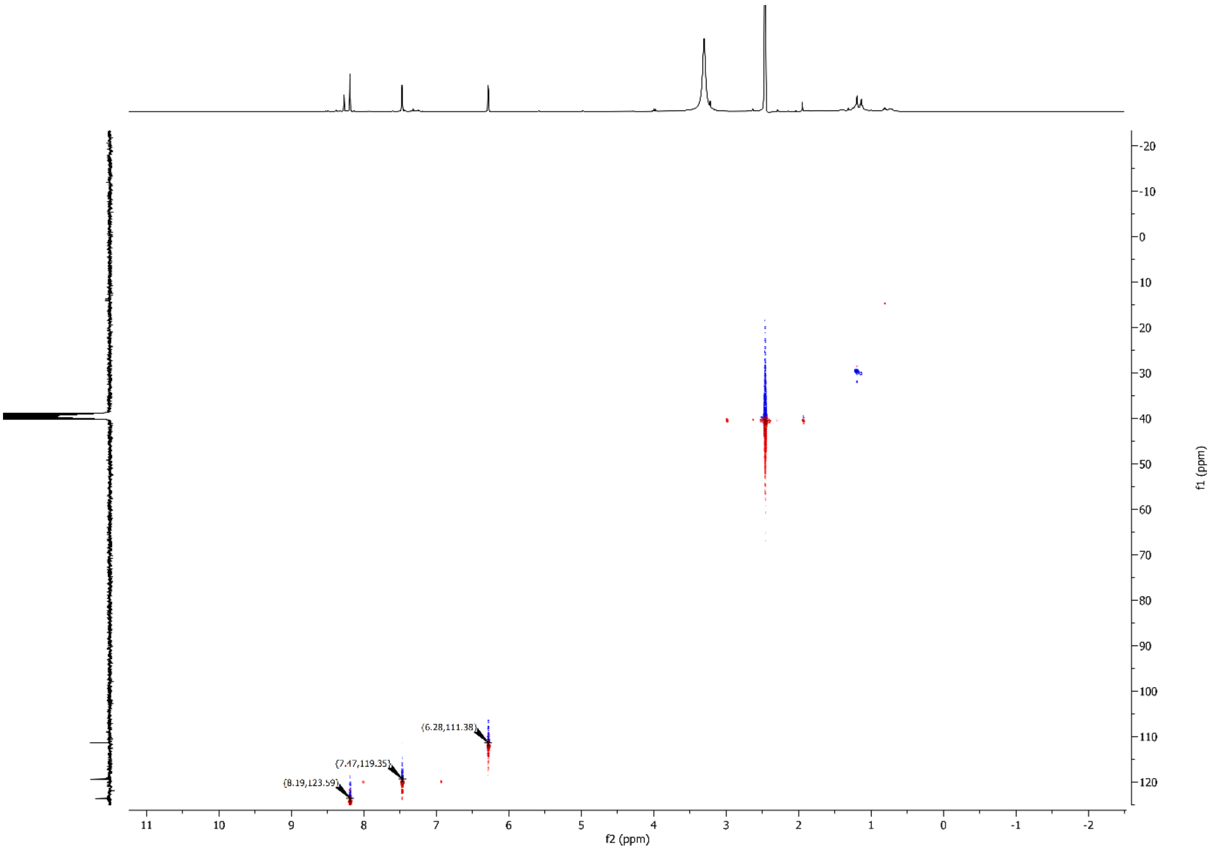

Figure S50. HSQC spectrum of compound **22.**

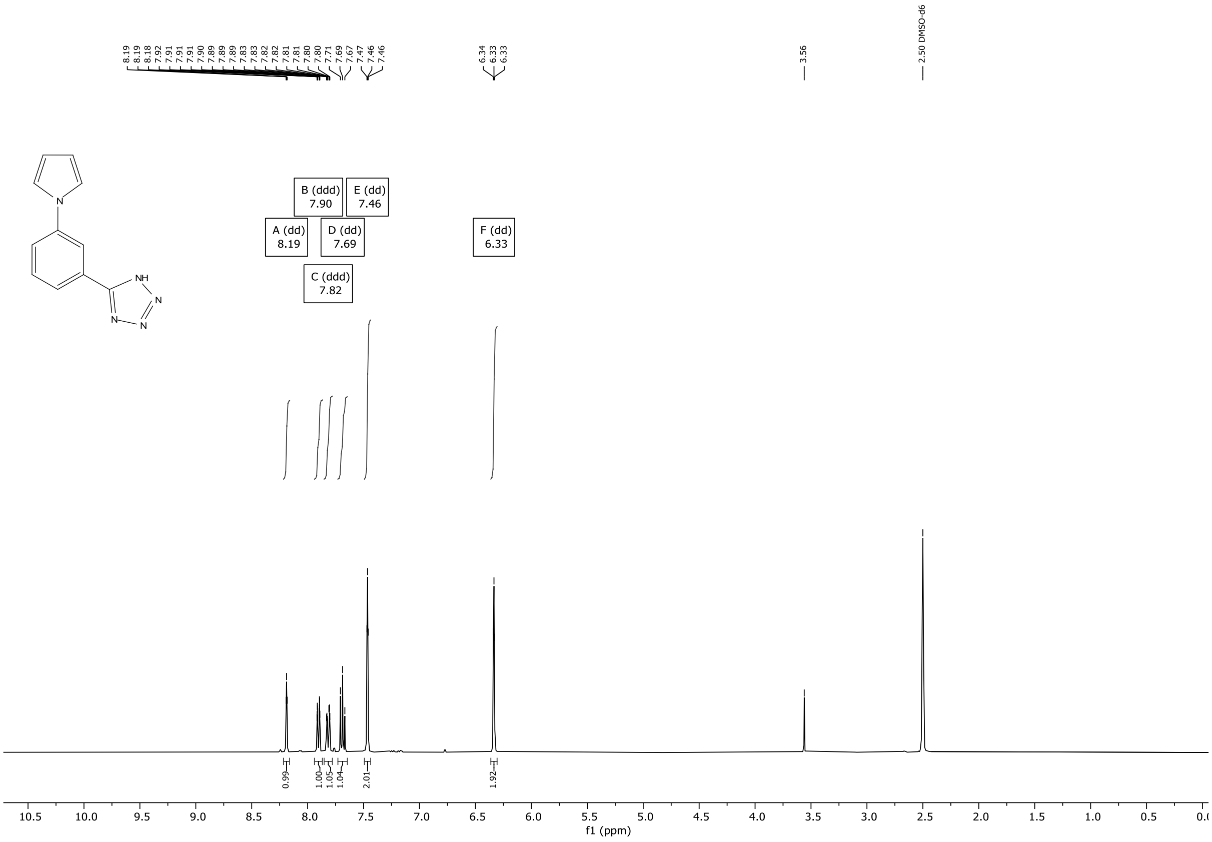

Figure S51. ^{1}H NMR spectrum of compound **23.**

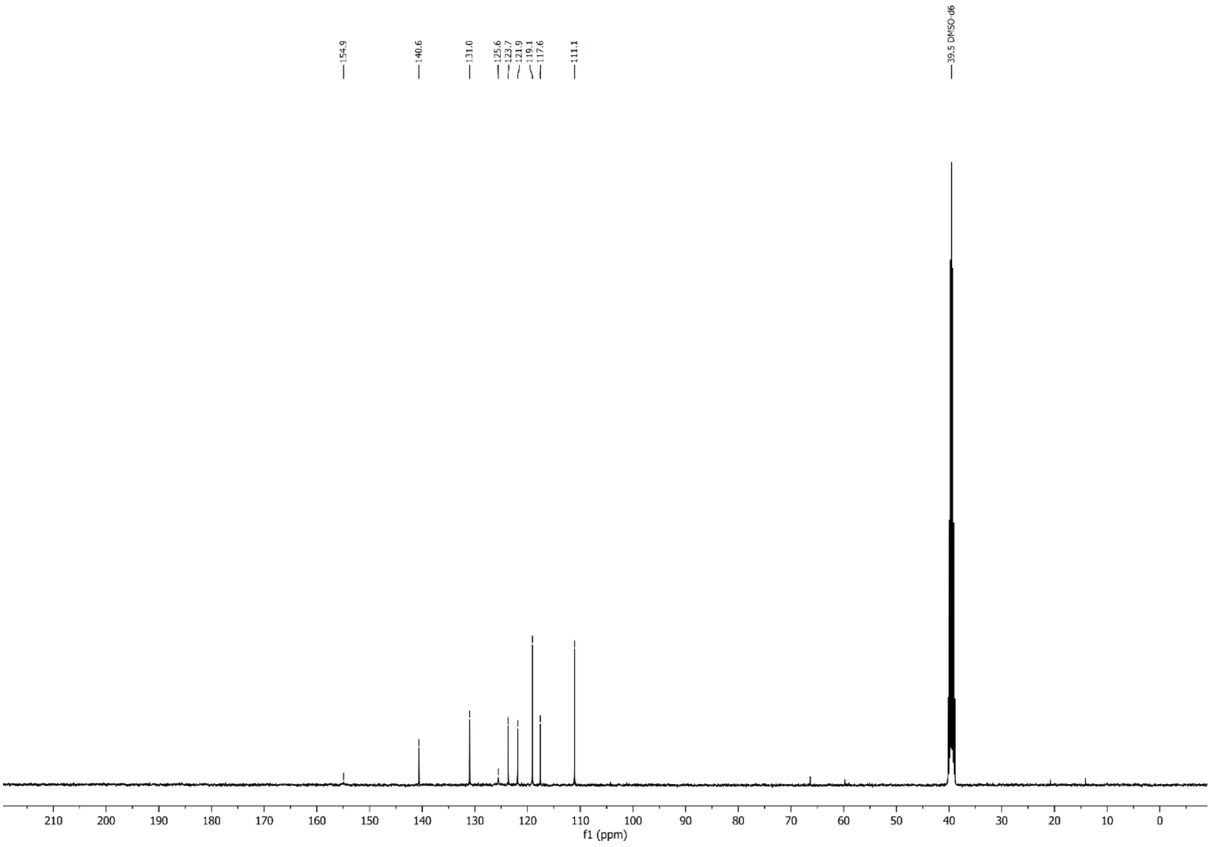

Figure S52. ^{13}C NMR spectrum of compound **23.**

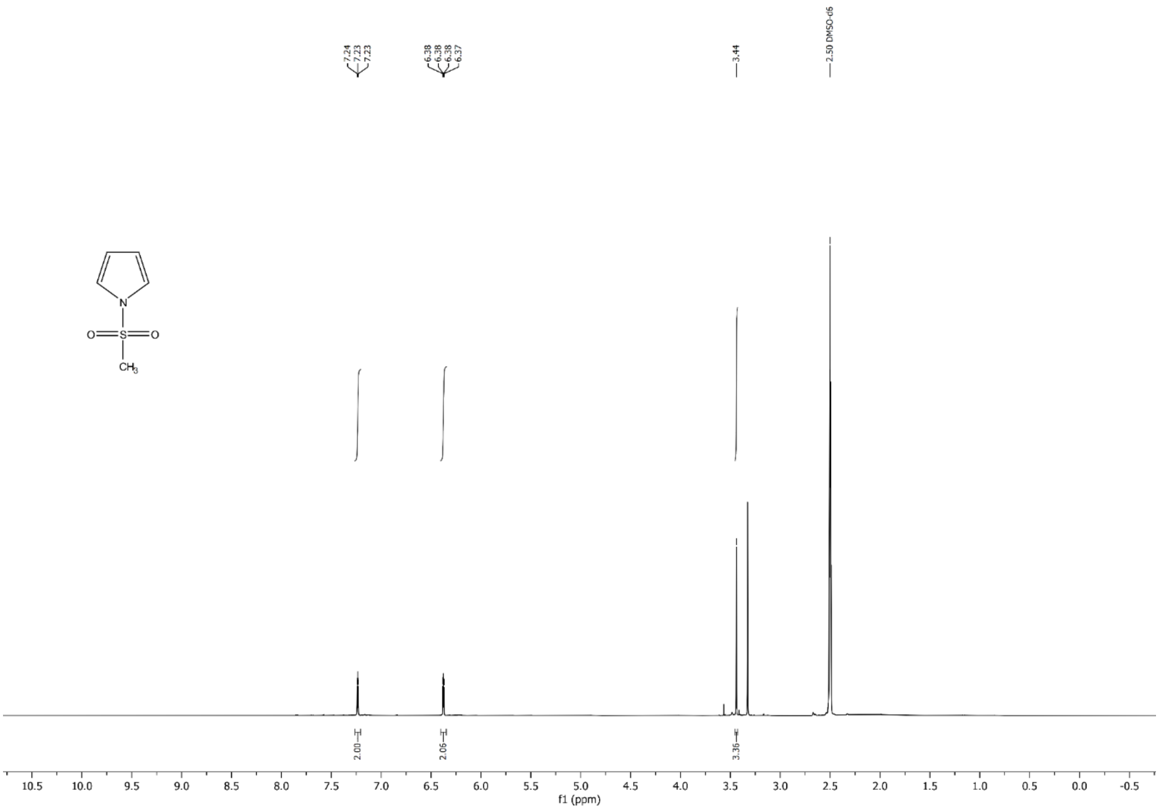

Figure S53. ^{1}H NMR spectrum of compound **24.**

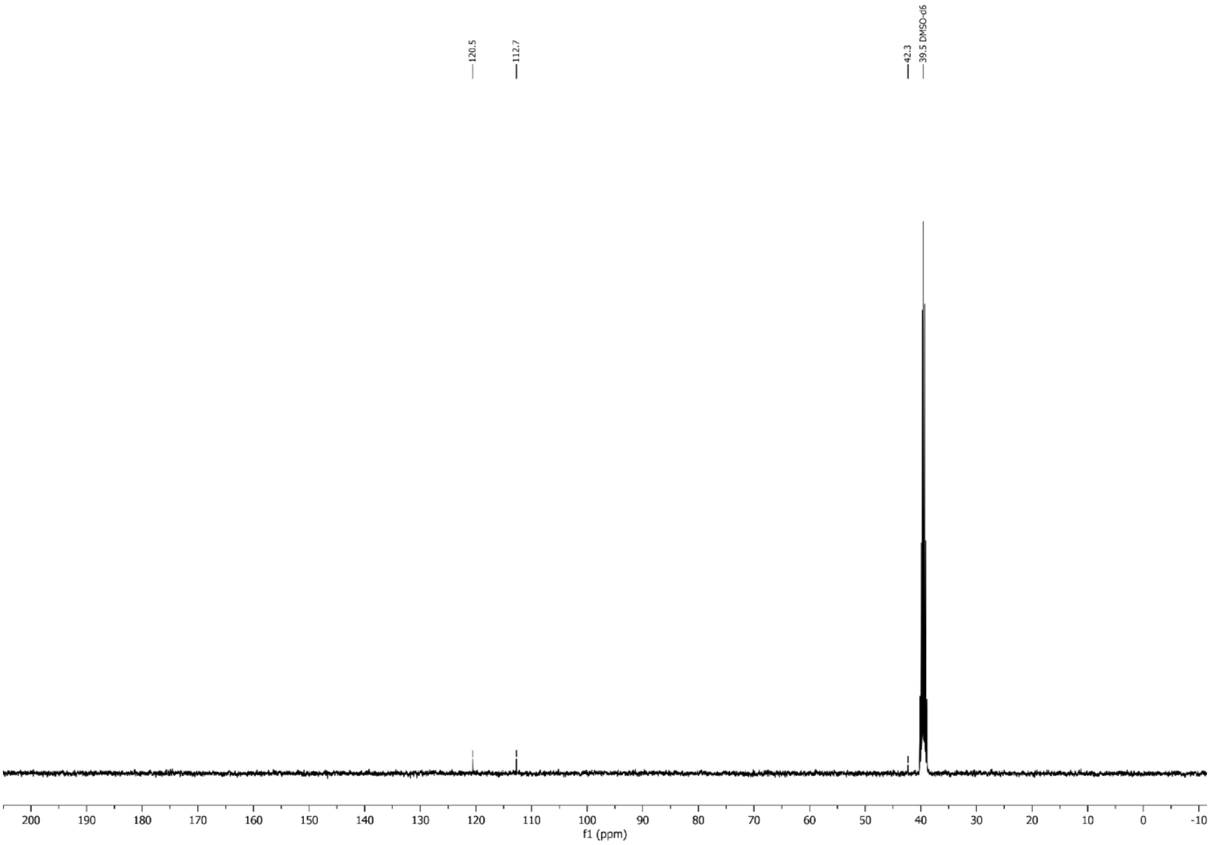

Figure S54. ^{13}C NMR spectrum of compound **24.**

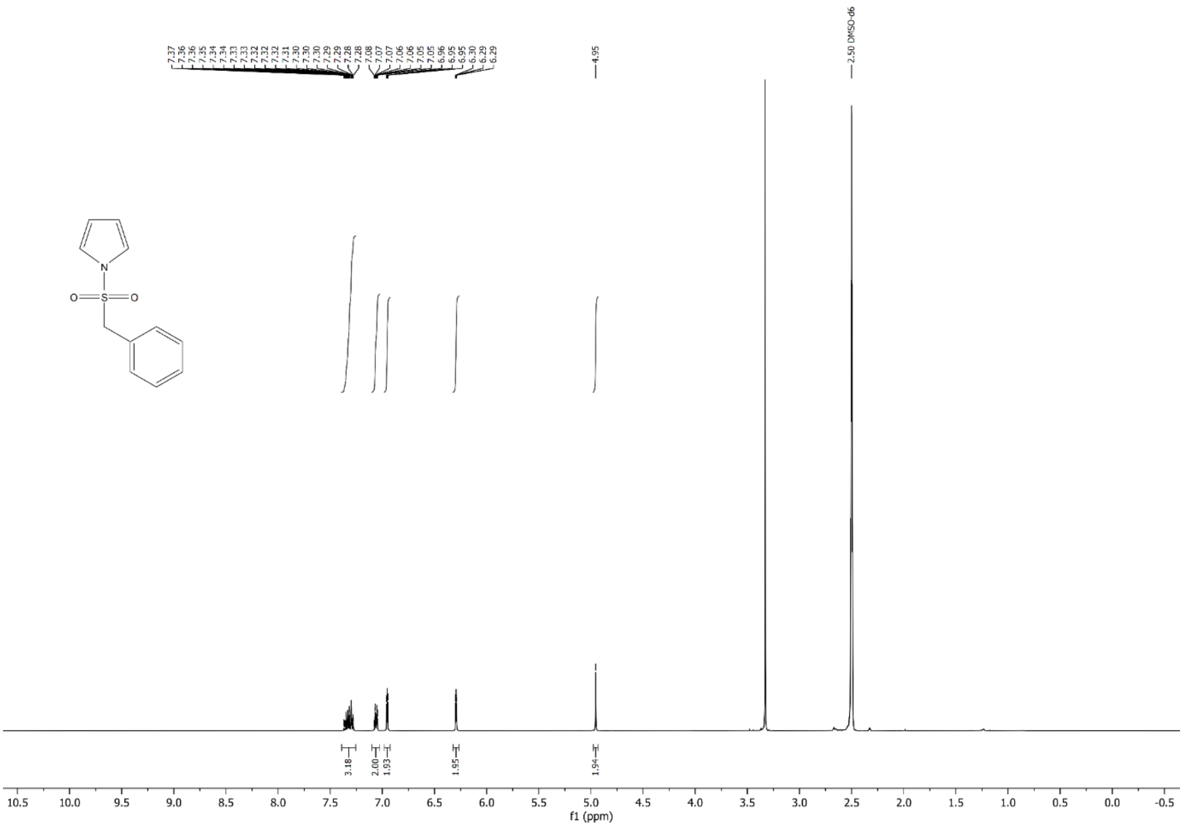

Figure S55. ^{1}H NMR spectrum of compound **25.**

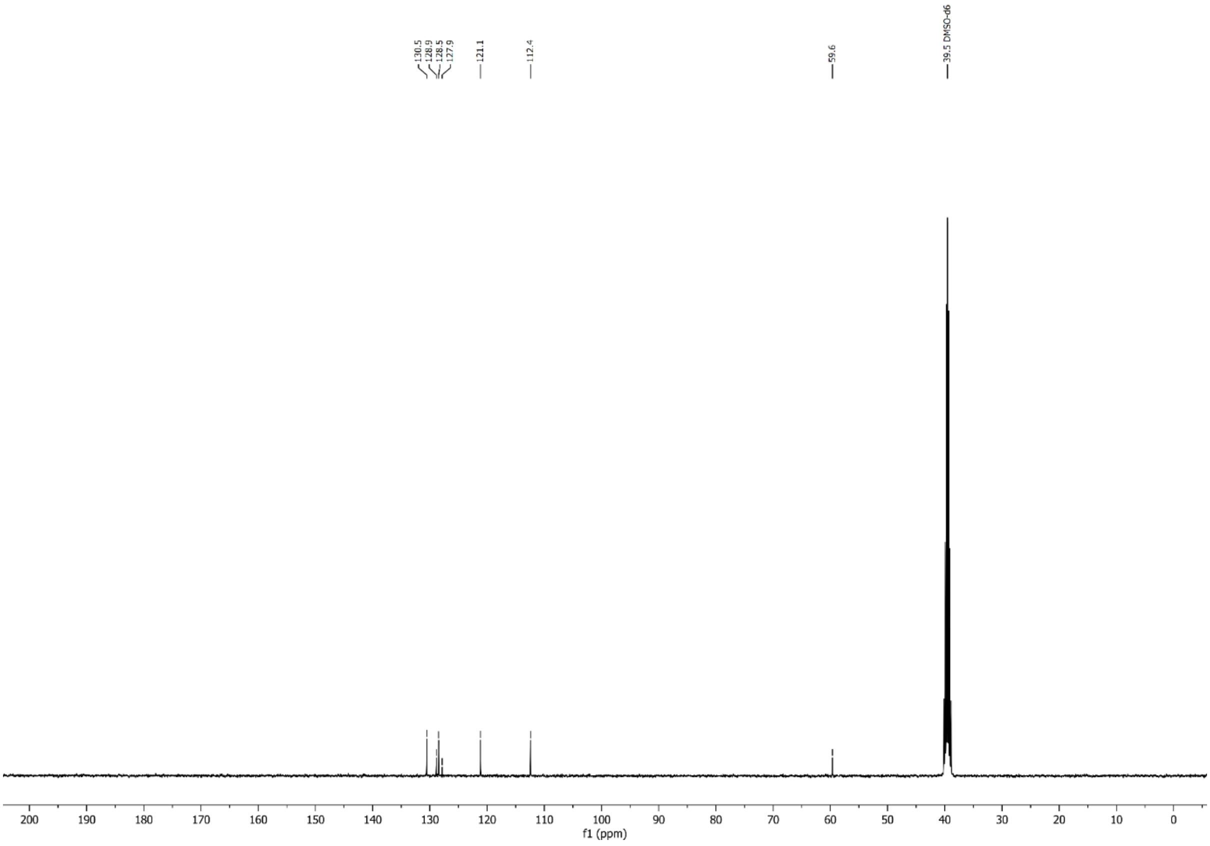

Figure S56. ^{13}C NMR spectrum of compound **25.**

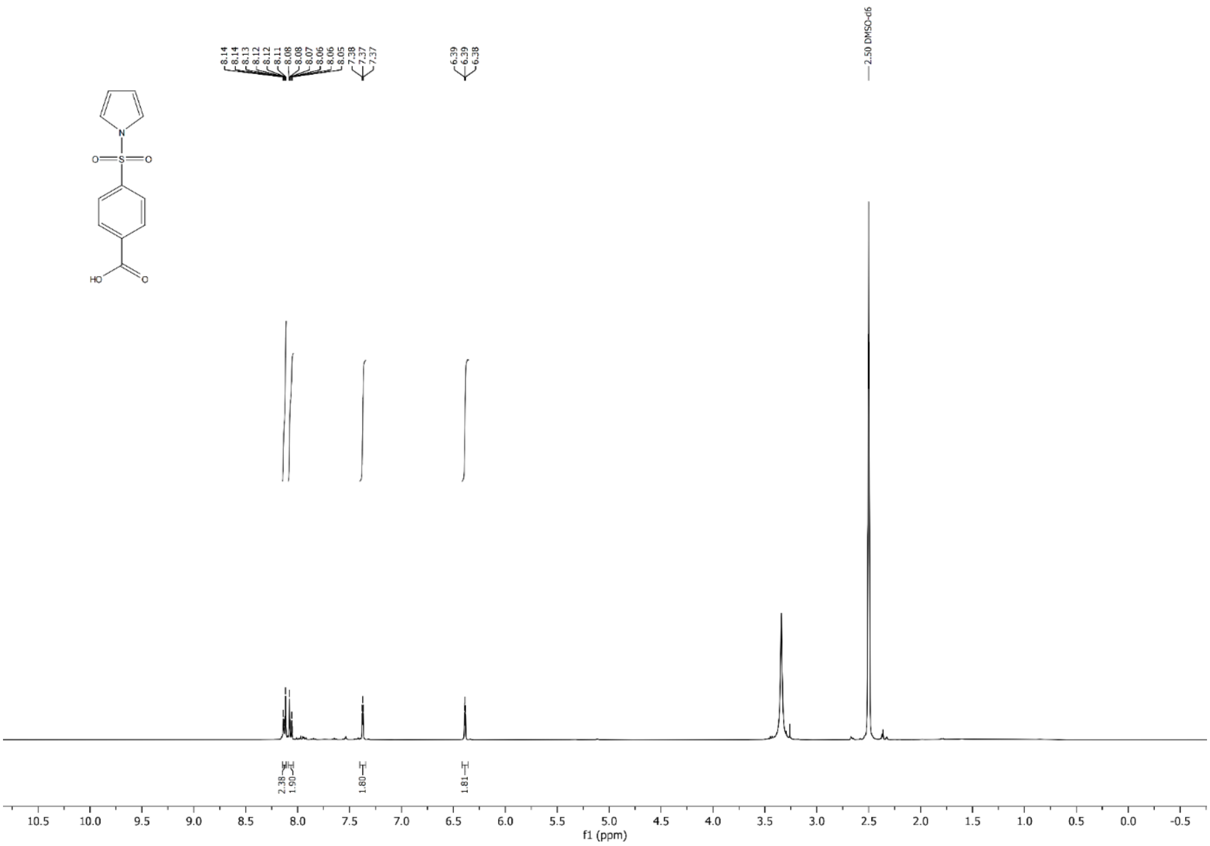

Figure S57. ^{1}H NMR spectrum of compound **26.**

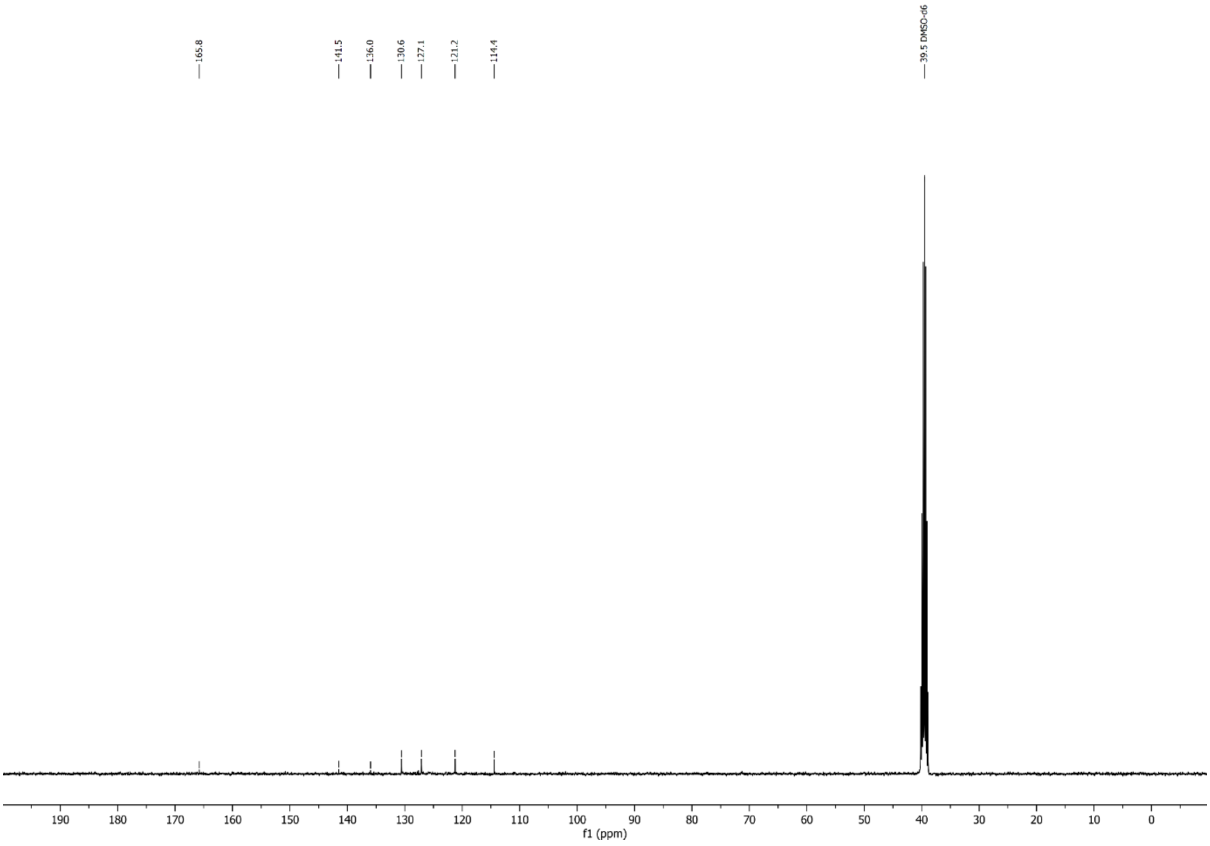

Figure S58. ^{13}C NMR spectrum of compound **26.**

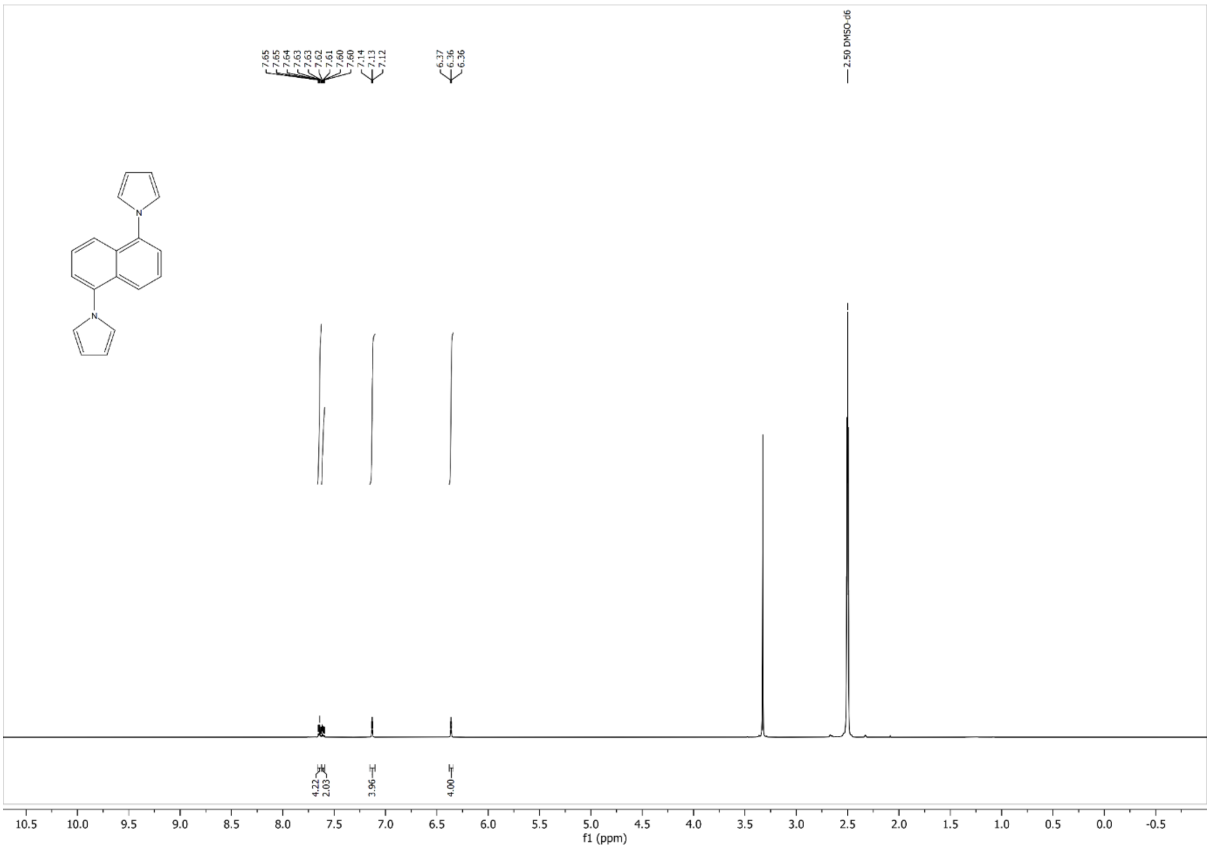

Figure S58. ^{1}H NMR spectrum of compound **27.**

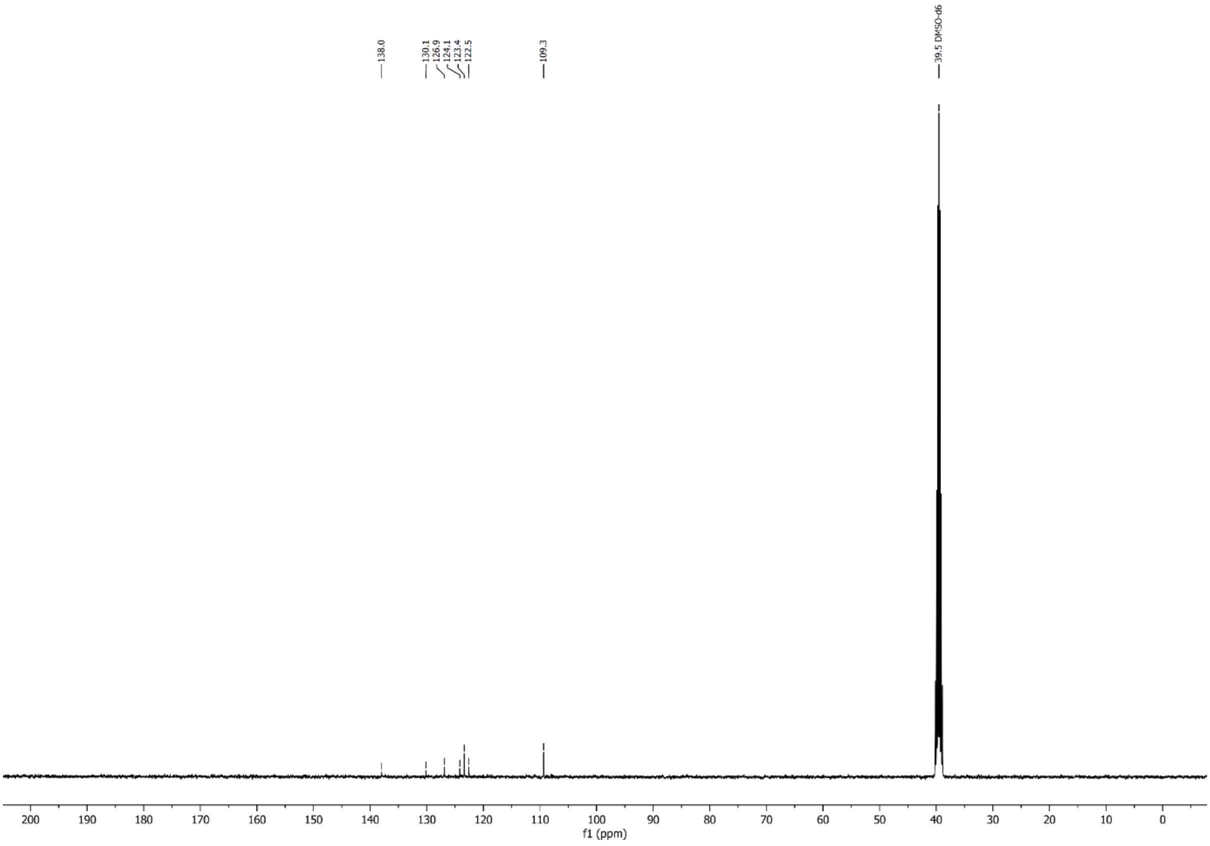

Figure S59. ^{13}C NMR spectrum of compound **27.**

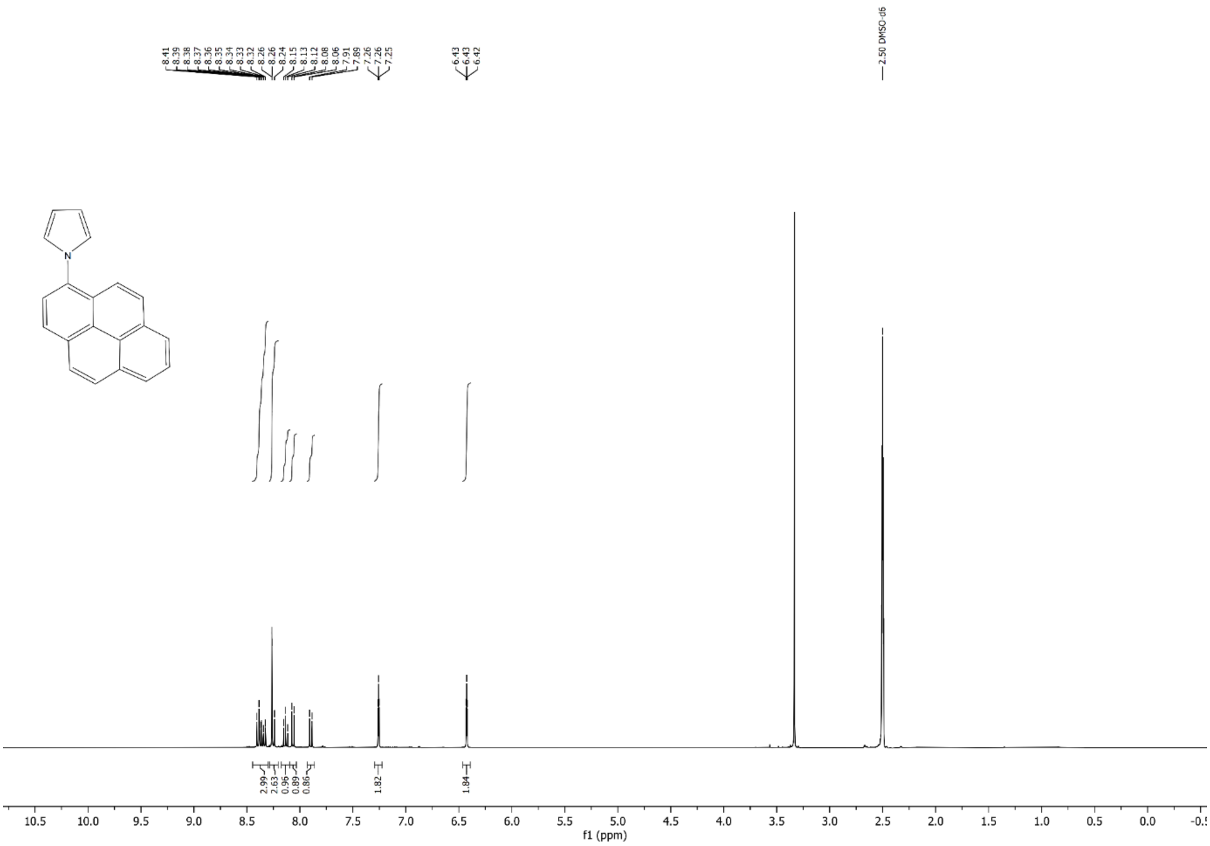

Figure S60. ^{1}H NMR spectrum of compound **28.**

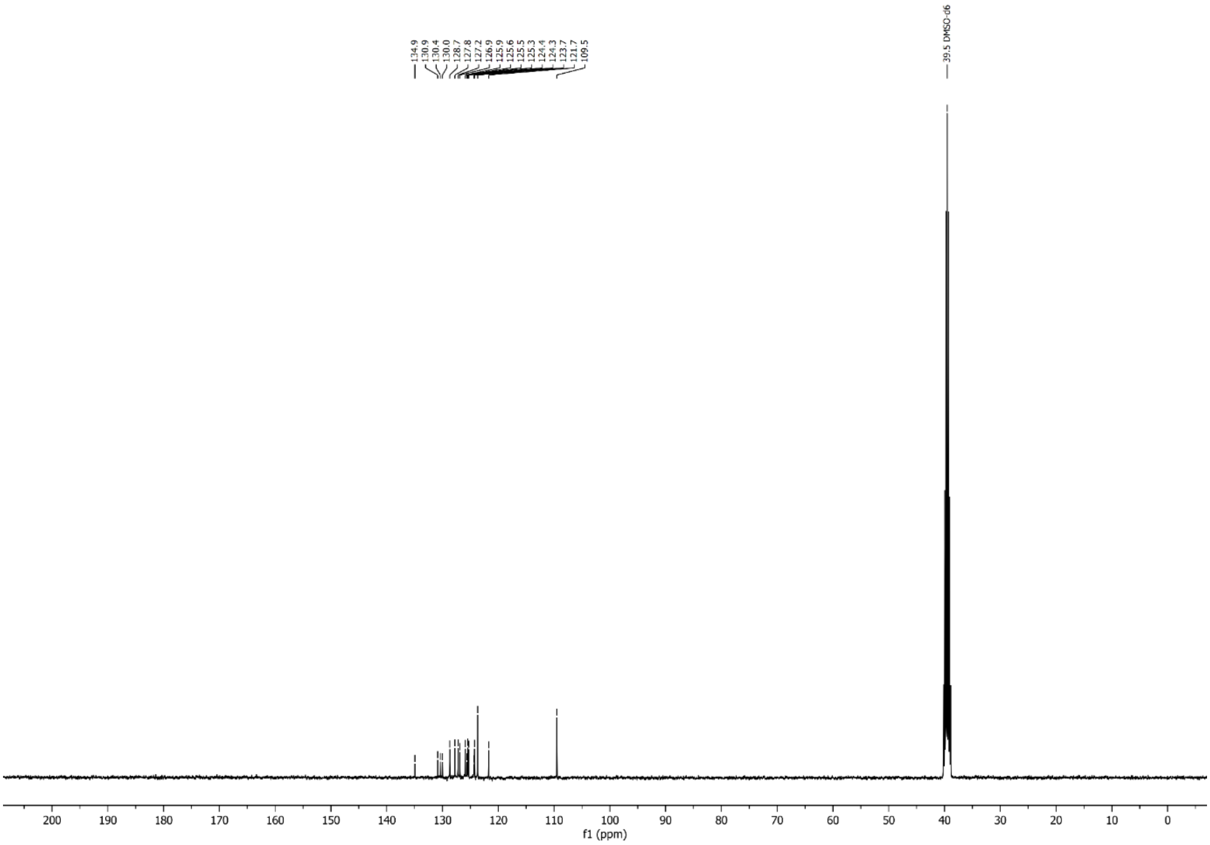

Figure S61. ^{13}C NMR spectrum of compound **28.**

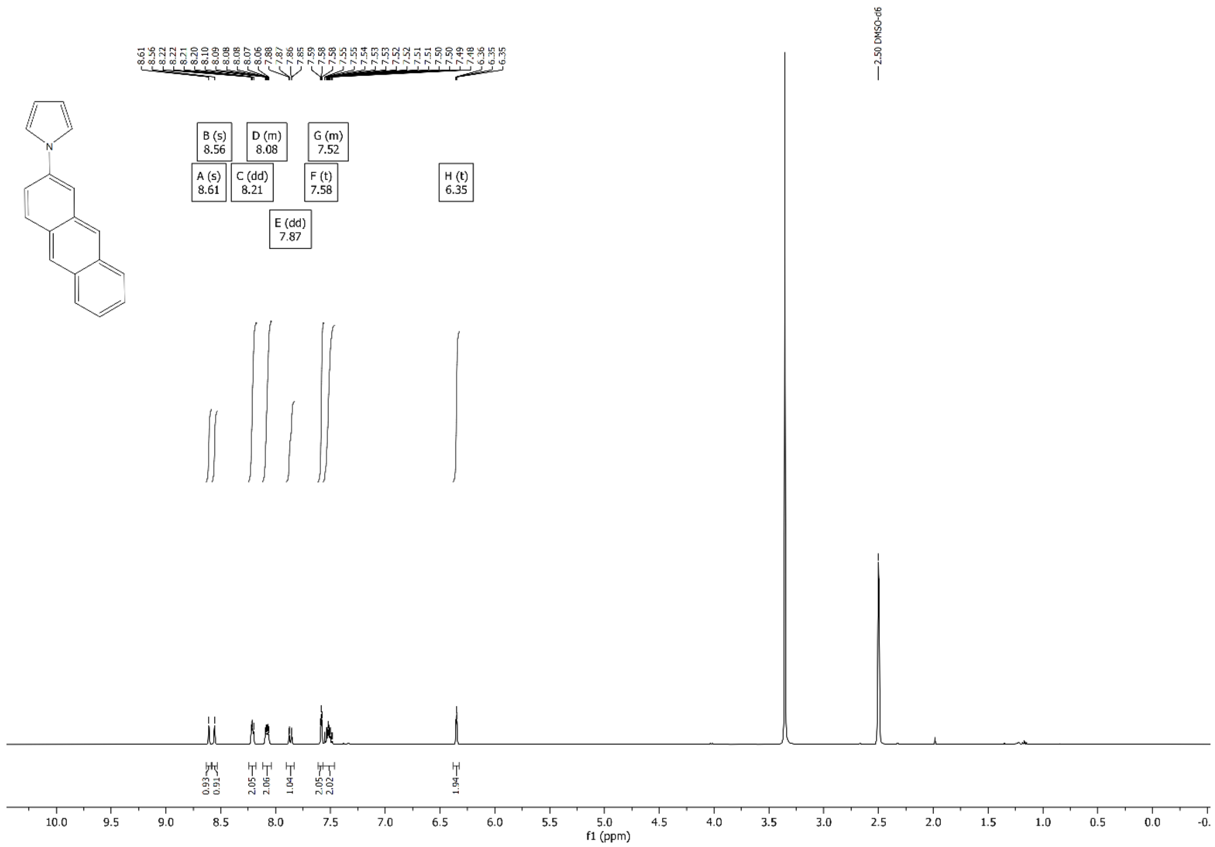

Figure S62. ^{1}H NMR spectrum of compound **29.**

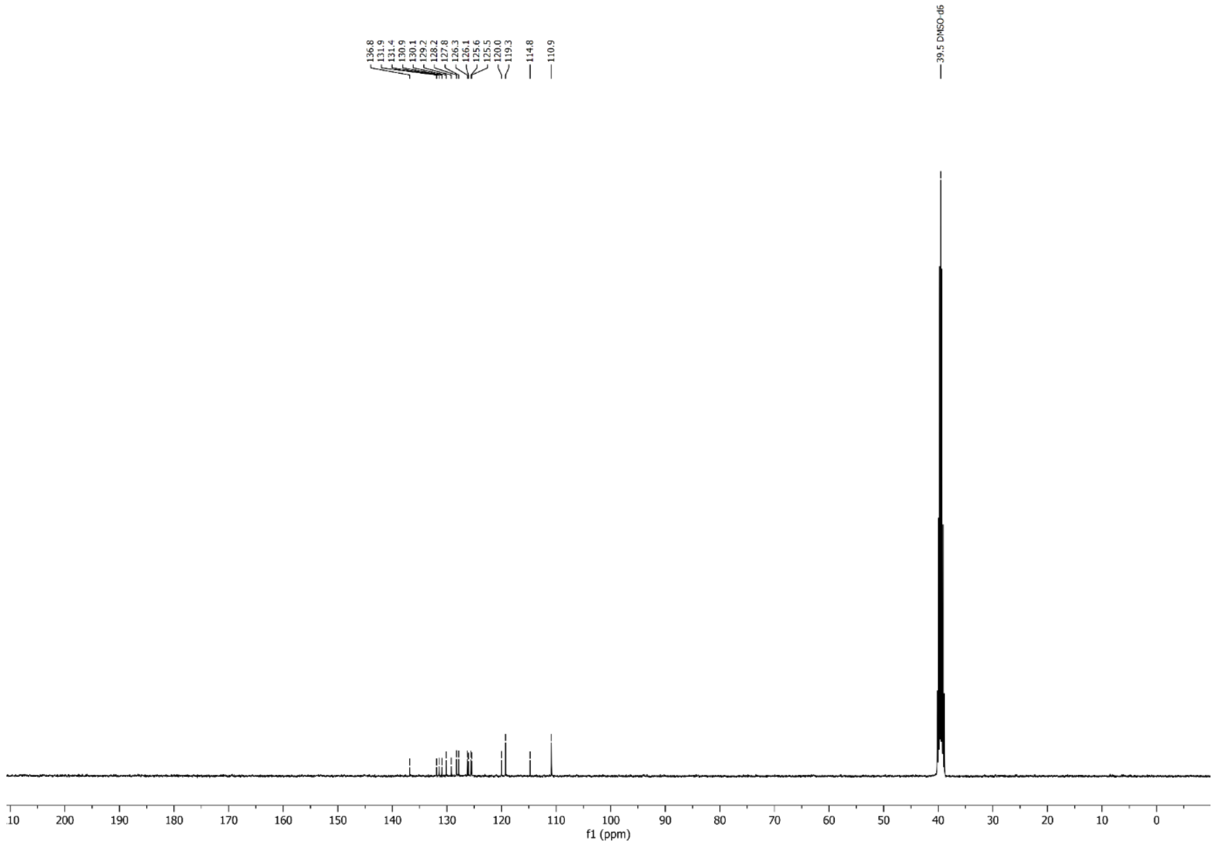

Figure S63. ^{13}C NMR spectrum of compound **29.**

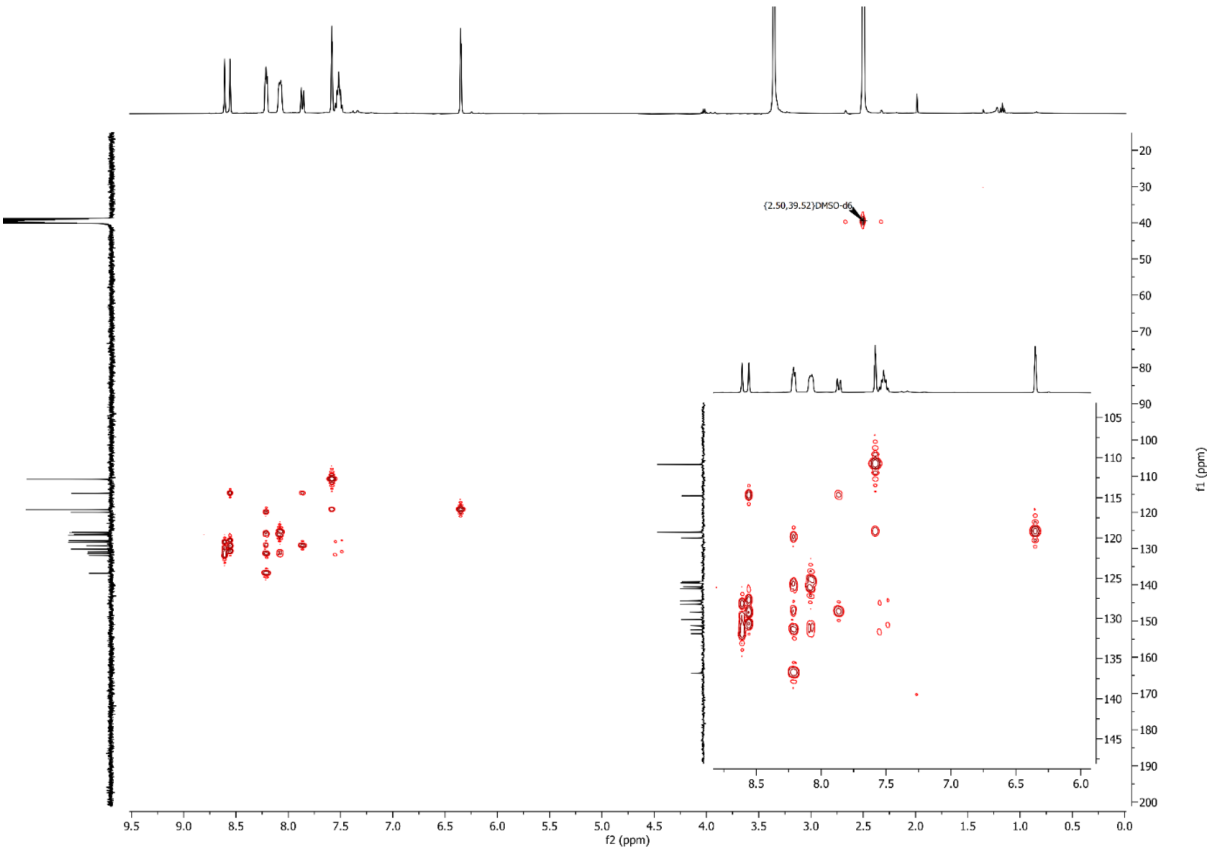

Figure S64. HMBC spectrum of compound **29.**

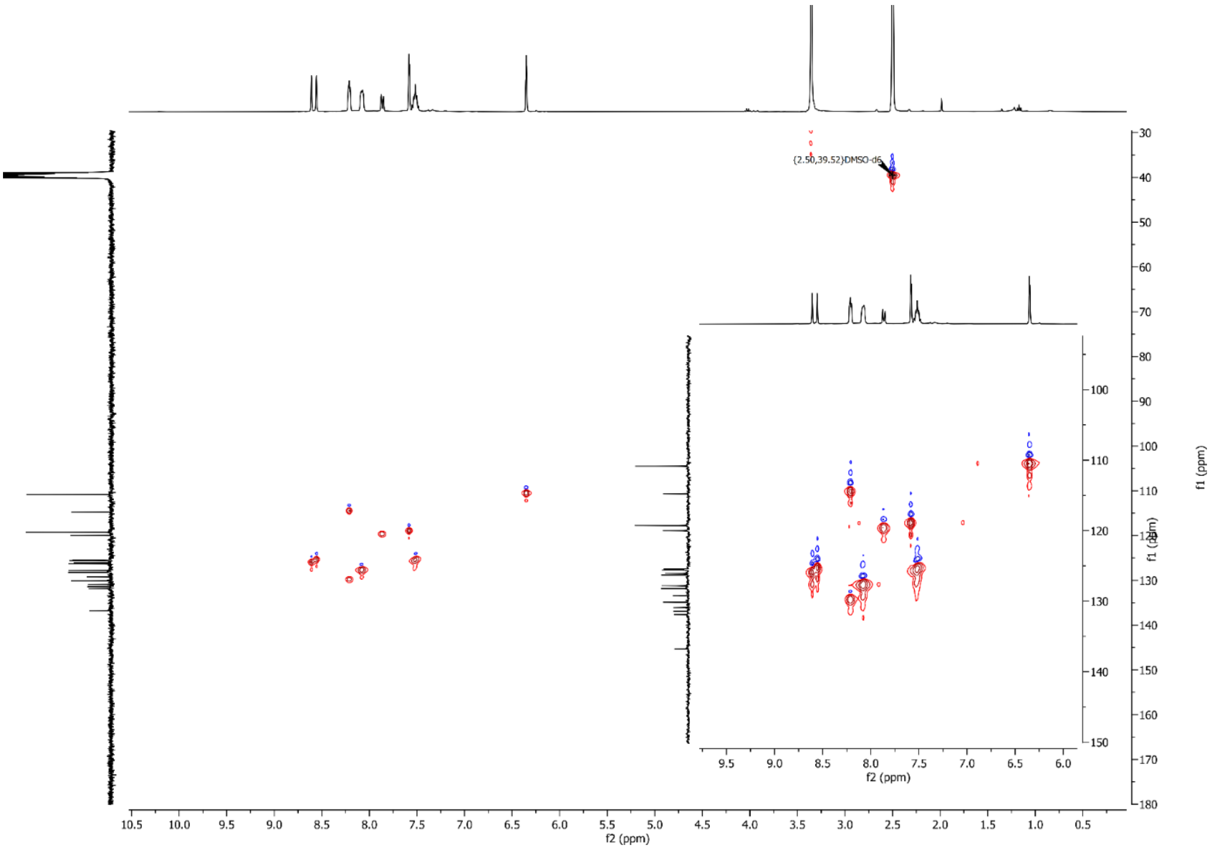

Figure S65. HSQC spectrum of compound **29.**

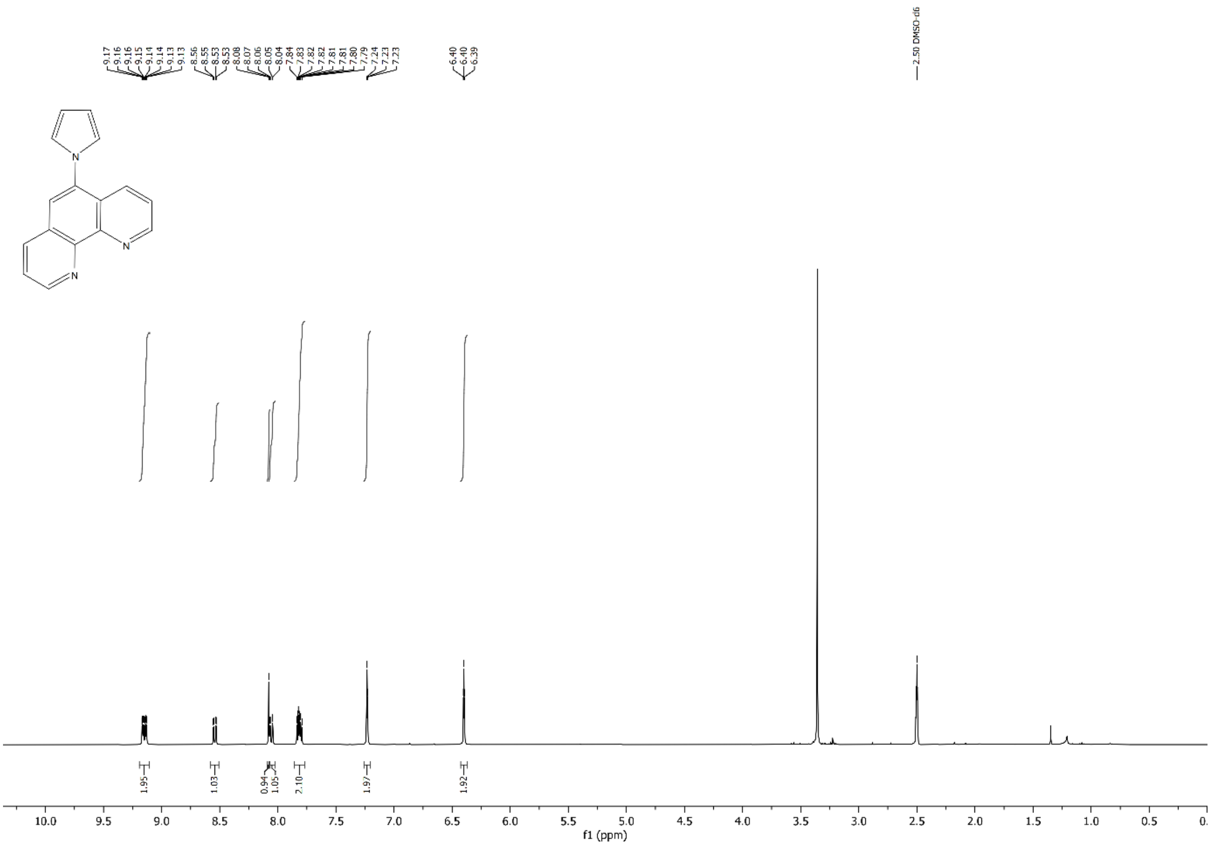

Figure S66. ^{1}H NMR spectrum of compound **30.**

References

(1) Patra, T.; Agasti, S.; Modak, A.; Maiti, D. Nickel-Catalyzed Hydrogenolysis of Unactivated Carbon–Cyano Bonds. *Chem. Commun.* **2013**, *49* (75), 8362–8364.

(2) Chen, H.; Han, J.; Wang, L. Diels–Alder Cycloadditions of N-Arylpyrroles via Aryne Intermediates Using Diaryliodonium Salts. *Beilstein J. Org. Chem. 1423* **2018**, *14* (1), 354–363.

(3) Nandi, D.; Siwal, S.; Mallick, K. A Carbon Nitride Supported Copper Nanoparticle Composite: A Heterogeneous Catalyst for the N-Arylation of Hetero-Aromatic Compounds. *New J. Chem.* **2017**, *41* (8), 3082–3088.

(4) Wu, Y. N.; Fu, M. C.; Shang, R.; Fu, Y. Nickel-Catalyzed Carboxylation of Aryl Iodides with Lithium Formate through Catalytic CO Recycling. *Chem. Commun.* **2020**, *56* (29), 4067–4069.

(5) Cho, E. J.; Senecal, T. D.; Kinzel, T.; Zhang, Y.; Watson, D. A.; Buchwald, S. L. The Palladium-Catalyzed Trifluoromethylation of Aryl Chlorides. *Science (80-.).* **2010**, *328* (5986), 1679–1681.

(6) Wani, R. R.; Chaudhari, H. K.; Takale, B. S. Solvent Free Synthesis of N-Substituted Pyrroles Catalyzed by Calcium Nitrate. *J. Heterocycl. Chem.* **2019**, *56* (4), 1337–1340.

(7) Bie, F.; Liu, X.; Cao, H.; Shi, Y.; Zhou, T.; Szostak, M.; Liu, C. Pd-Catalyzed Double-Decarbonylative Aryl Sulfide Synthesis through Aryl Exchange between Amides and Thioesters. *Org. Lett.* **2021**, *23* (20), 8098–8103.

(8) Wang, Y.; Pan, Z.; Cheng, X. L.; Zhang, K.; Zhang, X.; Qin, Y.; Fan, J.; Yan, T.; Han, T.; Shiu, K. K.; Hau, S. C. K.; Mak, N. K.; Kwong, D. W. J.; Liu, X.; Li, M.; Deng, G.; Zheng, Q.; Lu, J.; Li, D. A Red-Light-Activated Sulfonamide Porphycene for Highly Efficient Photodynamic Therapy against

Hypoxic Tumor. *Eur. J. Med. Chem.* **2021**, *209*, 112867.

(9) Lim, S.; Cho, H.; Jeong, J.; Jang, M.; Kim, H.; Cho, S. H.; Lee, E. Cobalt-Catalyzed Defluorosilylation of Aryl Fluorides via Grignard Reagent Formation. *Org. Lett.* **2020**, *22* (18), 7387–7392.

(10) Feng, Y.; Luo, H.; Yu, F.; Liao, Q.; Lin, L. Sodium-Iodide-Promoted Nickel-Catalyzed C–N Cross-Coupling of Aryl Chlorides and N-Nucleophiles under Visible-Light Irradiation. *Green Chem.* **2023**, *25* (6), 2361–2367.

(11) Llobet, A.; Masllorens, E.; Rodríguez, M.; Roglans, A.; Benet-Buchholz, J. Syntheses, Structures and Redox Properties of New Macrocyclic Triazatriolefinic Pd0 Complexes and Their Polypyrrole Modified Electrodes − Application to Heterogeneous Catalytic Suzuki Cross-Coupling Reactions. *Eur. J. Inorg. Chem.* **2004**, *2004* (8), 1601–1610.

(12) Rohit, K. R.; Meera, G.; Anilkumar, G. A Solvent-Free Manganese(II) -Catalyzed Clauson-Kaas Protocol for the Synthesis of N-Aryl Pyrroles under Microwave Irradiation. *J. Heterocycl. Chem.* **2022**, *59* (1), 194–200.

(13) Liu, K.; Lu, H.; Hou, L.; Qi, Z.; Teixeira, C.; Barbault, F.; Fan, B.-T.; Liu, S.; Jiang, S.; Xie, L. Design, Synthesis, and Biological Evaluation of N-Carboxyphenylpyrrole Derivatives as Potent HIV Fusion Inhibitors Targeting Gp41. *J. Med. Chem.* **2008**, *51* (24), 1601–1610.

(14) Enamine Ltd. EN300-186443.Nmr.Y2020. 2020.

(15) Laha, J. K.; Sharma, S.; Bhimpuria, R. A.; Dayal, N.; Dubey, G.; Bharatam, P. V. Integration of Oxidative Arylation with Sulfonyl Migration: One-Pot Tandem Synthesis of Densely Functionalized (NH)-Pyrroles. *New J. Chem.* **2017**, *41* (17), 8791–8803.

(16) Ozaki, T.; Yorimitsu, H.; Perry, G. J. P. Late-Stage Sulfonic Acid/Sulfonate Formation from Sulfonamides via Sulfonyl Pyrroles. *Tetrahedron* **2022**, *117–118*, 132830.

(17) Rudnitskaya, A.; Borkin, D. A.; Huynh, K.; Török, B.; Stieglitz, K. Rational Design, Synthesis, and Potency of N-Substituted Indoles, Pyrroles, and Triarylpyrazoles as Potential Fructose 1,6-Bisphosphatase Inhibitors. *ChemMedChem* **2010**, *5* (3), 384–389.

(18) Ma, F. P.; Li, P. H.; Li, B. Le; Mo, L. P.; Liu, N.; Kang, H. J.; Liu, Y. N.; Zhang, Z. H. A Recyclable Magnetic Nanoparticles Supported Antimony Catalyst for the Synthesis of N-Substituted Pyrroles in Water. *Appl. Catal. A Gen.* **2013**, *457*, 34–41.

(19) Bandyopadhyay, D.; Mukherjee, S.; Granados, J. C.; Short, J. D.; Banik, B. K. Ultrasound-Assisted Bismuth Nitrate-Induced Green Synthesis of Novel Pyrrole Derivatives and Their Biological Evaluation as Anticancer Agents. *Eur. J. Med. Chem.* **2012**, *50*, 209–215.

(20) Si, Z.; Li, X.; Li, X.; Zhang, H. Synthesis, Photophysical Properties, and Theoretical Studies on Pyrrole-Containing Bromo Re(I) Complex. *J. Organomet. Chem.* **2009**, *694* (23), 3742–3748.
